# The Myb73–GDPD2–GA2ox1 transcriptional regulatory module confers phosphate deficiency tolerance in soybean

**DOI:** 10.1093/plcell/koae041

**Published:** 2024-02-12

**Authors:** Dandan Hu, Ruifan Cui, Ke Wang, Yuming Yang, Ruiyang Wang, Hongqing Zhu, Mengshi He, Yukun Fan, Le Wang, Li Wang, Shanshan Chu, Jinyu Zhang, Shanshan Zhang, Yifei Yang, Xuhao Zhai, Haiyan Lü, Dandan Zhang, Jinshe Wang, Fanjiang Kong, Deyue Yu, Hengyou Zhang, Dan Zhang

**Affiliations:** Collaborative Innovation Center of Henan Grain Crops, College of Agronomy, Henan Agricultural University, Zhengzhou 450002, China; Collaborative Innovation Center of Henan Grain Crops, College of Agronomy, Henan Agricultural University, Zhengzhou 450002, China; Collaborative Innovation Center of Henan Grain Crops, College of Agronomy, Henan Agricultural University, Zhengzhou 450002, China; Collaborative Innovation Center of Henan Grain Crops, College of Agronomy, Henan Agricultural University, Zhengzhou 450002, China; Collaborative Innovation Center of Henan Grain Crops, College of Agronomy, Henan Agricultural University, Zhengzhou 450002, China; Collaborative Innovation Center of Henan Grain Crops, College of Agronomy, Henan Agricultural University, Zhengzhou 450002, China; Collaborative Innovation Center of Henan Grain Crops, College of Agronomy, Henan Agricultural University, Zhengzhou 450002, China; Collaborative Innovation Center of Henan Grain Crops, College of Agronomy, Henan Agricultural University, Zhengzhou 450002, China; State Key Laboratory of Black Soils Conservation and Utilization, Key Laboratory of Soybean Molecular Design Breeding, Northeast Institute of Geography and Agroecology, Chinese Academy of Sciences, Harbin 150081, China; National Center for Soybean Improvement, National Key Laboratory of Crop Genetics and Germplasm Enhancement, Nanjing Agricultural University, Nanjing 210095, China; Collaborative Innovation Center of Henan Grain Crops, College of Agronomy, Henan Agricultural University, Zhengzhou 450002, China; Collaborative Innovation Center of Henan Grain Crops, College of Agronomy, Henan Agricultural University, Zhengzhou 450002, China; Collaborative Innovation Center of Henan Grain Crops, College of Agronomy, Henan Agricultural University, Zhengzhou 450002, China; Collaborative Innovation Center of Henan Grain Crops, College of Agronomy, Henan Agricultural University, Zhengzhou 450002, China; Collaborative Innovation Center of Henan Grain Crops, College of Agronomy, Henan Agricultural University, Zhengzhou 450002, China; Collaborative Innovation Center of Henan Grain Crops, College of Agronomy, Henan Agricultural University, Zhengzhou 450002, China; State Key Laboratory of Agricultural Microbiology, Center of Integrative Biology, College of Life Science and Technology, Huazhong Agricultural University, Wuhan 430070, China; Zhengzhou National Subcenter for Soybean Improvement, Henan Academy of Agricultural Sciences, Zhengzhou 450002, China; School of Life Sciences, Guangzhou University, Guangzhou 510006, China; National Center for Soybean Improvement, National Key Laboratory of Crop Genetics and Germplasm Enhancement, Nanjing Agricultural University, Nanjing 210095, China; State Key Laboratory of Black Soils Conservation and Utilization, Key Laboratory of Soybean Molecular Design Breeding, Northeast Institute of Geography and Agroecology, Chinese Academy of Sciences, Harbin 150081, China; Collaborative Innovation Center of Henan Grain Crops, College of Agronomy, Henan Agricultural University, Zhengzhou 450002, China

## Abstract

Phosphorus is indispensable in agricultural production. An increasing food supply requires more efficient use of phosphate due to limited phosphate resources. However, how crops regulate phosphate efficiency remains largely unknown. Here, we identified a major quantitative trait locus, *qPE19*, that controls 7 low-phosphate (LP)-related traits in soybean (*Glycine max*) through linkage mapping and genome-wide association studies. We identified the gene responsible for *qPE19* as *GLYCEROPHOSPHORYL DIESTER PHOSPHODIESTERASE2* (*GmGDPD2*), and haplotype 5 represents the optimal allele favoring LP tolerance. Overexpression of *GmGDPD2* significantly affects hormone signaling and improves root architecture, phosphate efficiency and yield-related traits; conversely, CRISPR/Cas9-edited plants show decreases in these traits. GmMyb73 negatively regulates *GmGDPD2* by directly binding to its promoter; thus, GmMyb73 negatively regulates LP tolerance. GmGDPD2 physically interacts with GA 2-oxidase 1 (GmGA2ox1) in the plasma membrane, and overexpressing *GmGA2ox1* enhances LP-associated traits, similar to *GmGDPD2* overexpression. Analysis of double mutants for *GmGDPD2* and *GmGA2ox1* demonstrated that GmGDPD2 regulates LP tolerance likely by influencing auxin and gibberellin dose-associated cell division in the root. These results reveal a regulatory module that plays a major role in regulating LP tolerance in soybeans and is expected to be utilized to develop phosphate-efficient varieties to enhance soybean production, particularly in phosphate-deficient soils.

## Teaser

The major quantitative trait locus gene *GmGDPD2* controls root and phosphorus efficiency traits and interacts with Myb73 and GA2ox1 to enhance yield traits.

**IN A NUTSHELL**

**Background:** Phosphorus (P), produced from nonrenewable phosphate rock, is an essential nutrient for plant growth and development. However, ∼40% of the world's arable land is P deficient. Plants have evolved a series of complex strategies that allow them to adapt to P-deficient soils, including phosphate activation, absorption, transport, storage, and reuse. The growth of soybean, a major source of plant-based protein worldwide, requires a large amount of P. Therefore, the availability of P in soils is key to ensuring optimal soybean yields. There is thus an urgent need to develop a genetic solution to improve P uptake and conserve phosphate resources globally.

**Question:** Do soybean plants themselves hold the key to developing new high-yield soybean varieties? Can a deeper understanding of the molecular mechanisms underlying phosphate absorption and utilization in soybean help us develop strategies to improve P uptake and use efficiency in this important crop?

**Findings:** We uncovered a major low-P tolerance gene in soybean, *GmGDPD2*, through genetic methods. Overexpression of this gene significantly promoted root growth and development and increased root uptake of phosphate and yield. By contrast, knocking out this gene inhibited root growth and reduced P absorption, reducing yield. We also identified a *GmGDPD2*-interacting protein, GA2ox1. GmMyb73 inhibited *GmGDPD2* expression by binding to its promoter region. These results allowed us to identify the Myb73–GDPD2–GA2ox1 regulatory module, which helps plants acquire more P from the soil by promoting root growth.

**Next steps:** We will continue examining the Myb73–GDPD2–GA2ox1 module to dissect the detailed mechanism underlying P efficiency. For example, we found that an acid phosphatase gene *GmAPA17* is significantly upregulated in the *gdpd2* mutant, which may be an important gene downstream of the module. More genes regulated by *GmGDPD2* and their regulatory mechanisms remain to be explored.

## Introduction

Phosphorus (P) is an important macronutrient essential for plant growth and development, accounting for approximately 0.2% of plant dry weight (Schachtman et al. 1998). It is estimated that nearly one-third of global cropland exhibits P deficiency, requiring P fertilization to improve crop yield (Bates 1997; Lynch 2011). However, only 15% to 20% of applied P in fertilizers can be taken up by crops, and overconsumption of P fertilizer worldwide has caused an increase in the cost of P fertilizer and farming in addition to the concerns of environmental pollution (Lopez-Arredondo et al. 2014; Dai et al. 2023). Even worse, the sources of P rock are finite and are projected to be exhausted in the next decades (Kauwenbergh et al. 2013), which is a growing concern that needs to be addressed to meet the increasing demand for more food globally (Godfray et al. 2010). Tapping into genetic variation and developing P highly efficient crops that can maximize phosphorate (Pi) acquisition and utilization is crucial for ensuring sustainable agricultural production.

Plants have evolved a series of adaptive responses, including modified root architecture and hormone regulatory effects, genetic changes, and domestic adaptation, to increase Pi acquisition and meet their growth and survival needs (Parry 2018; Wang et al. 2022a). The alterations in root architecture mainly include reduced primary root growth, an increased number of lateral roots, or prolonged lateral roots, which collectively enhance the root surface areas (RA), allowing the plant to absorb Pi more effectively (Talboys et al. 2014; Gu et al. 2016; Bhosale et al. 2018; Giri et al. 2018; Villaecija-Aguilar et al. 2022). The changes in these morphological and physiological traits have been used as representative traits to evaluate the responsiveness of a plant to low-Pi (LP) stress or to identify quantitative trait loci (QTL) or genes using linkage mapping or association analysis (Zhang et al. 2016; Yang et al. 2023). Some recent studies have also reported proteins that regulate root development through hormone-associated pathways in response to LP stress, such as KARRIKIN INSENSITIVE2 (AtKAI2) and F-box protein MORE AXILLIARY BRANCHING2 (AtMAX2) (Villaecija-Aguilar et al. 2022), bHLH transcription factors ROOT HAIR DEFECTIVE SIX-LIKE2 (AtRSL2) and AtRSL4 (Vijayakumar et al. 2016), and auxin influx carrier (AtAUX1) and OsAUX1 (Bhosale et al. 2018; Giri et al. 2018). Of these, AUX1 mediates auxin remobilization from the root tip to root hair cells under LP stress and triggers the expression of *AUXIN RESPONSE FACTOR19* (*AtARF19*), *AtRSL2*, and *AtRSL4* to promote hair cell elongation. Another auxin protein, PIN-LIKES7 (AtPILS7), an auxin efflux carrier, was identified by a genome-wide association study (GWAS) of Pi concentration in Arabidopsis (*Arabidopsis thaliana*) plants (Yi et al. 2021). These studies indicate the important role of hormones such as auxin, as well as the ethylene pathway (Roldan et al. 2013; Song and Liu 2015), in regulating LP responsiveness. Few studies reported the involvement of gibberellic acid (GA) in LP tolerance. Devaiah et al. (2009) showed that overexpression of LP-responsive *MYB62* caused GA-deficient phenotypes in *Arabidopsis* that could be rescued by exogenous GA application (Devaiah et al. 2009), while the components bridging *MYB62* and GA pathways have not been identified. On the other hand, overexpression of the Pi signaling gene *PHOSPHATE STARVATION RESPONSE REGULATOR3-A1* (*TaPHR3-A1*) greatly increased yield-related traits in bread wheat (*Triticum aestivum*) and rice (*Oryza sativa*) (Zheng et al. 2020), demonstrating the potential of Pi tolerance genes for crop improvement.

Soybean (*Glycine max*) is the most cultivated oilseed crop (Anderson et al. 2019) and is a primary source of both high-quality protein and vegetable oil. It accounts for approximately 68% and 27% of world plant meal and oil production, respectively, for human and animal consumption worldwide (https://fas.usda.gov/data/oilseeds-world-markets-and-trade). Soybean growth and development require abundant Pi from soil; therefore, Pi deficiency is more problematic than other nutrient deficiencies in constraining soybean yield production (Gowin 1997; Abdelrahman et al. 2018). Pi deficiency may cause an increase in the root-to-shoot growth ratio and severe flower and pod abscission rates (Zhang et al. 2010), affect the development of root nodules (Le Roux et al. 2014), and decrease photosynthesis, yield, and quality in soybean (Li et al. 2016; Liu 2021). Given the increasing demand for plant-based protein globally (FAO 2021), the development of Pi-efficient soybean varieties is crucial for sustaining or enhancing soybean production, particularly in Pi-deficient soils.

Soybean cultivars differ greatly in LP responsiveness (Lu et al. 2018; Wang et al. 2019; Yang et al. 2020b; Wang et al. 2022b), and multiple QTL have been identified using a variety of LP-associated traits, such as root-related traits, biomass, organic acid content, Pi content, and efficiency, as well as photosynthesis-related traits (Zhang et al. 2014; Monnot et al. 2021; Yi et al. 2021; Chien et al. 2022; Schneider et al. 2022; Xu et al. 2023; Yang et al. 2023). However, rare genes for the major QTL have been cloned (Song et al. 2014; Zhang et al. 2014; Yang et al. 2023); thus, the underlying mechanism remains largely unknown. The current knowledge about Pi deficiency tolerance in soybean has been gained from the characterization of Pi deficiency-responsive genes that were mainly identified in differential expression analysis or reverse genetics-based homologous cloning; the genes identified include those encoding Pi transporters [*PHOSPHATE TRANSPORTER 1;4* (*GmPT4*), *GmPT5*, *GmPT7*] (Qin et al. 2012; Chen et al. 2019; Guo et al. 2022), purple acid phosphatase [*PURPLE ACID PHOSPHATASE 1* (*GmPAP1*), *GmPAP4*, *GmPAP7*, *GmPAP14*, *GmPAP17*, *GmPAP21*, *GmPAP33*, *GmPAP35*] (Bhadouria and Giri 2022; Xu et al. 2022), transcription factors [*PHOSPHATE STARVATION RESPONSE REGULATOR 25* (*GmPHR25*), *GmWRKY6*, *GmWRKY46*] (Xue et al. 2017; Liu et al. 2022; Wang et al. 2023b), or ethylene regulators [*ETHYLENE RESPONSE FACTOR 1* (*GmERF1*), *ETHYLENE-OVERPRODUCTION PROTEIN 1* (*GmETO1*)] (Zhang et al. 2020; Wang et al. 2023b). The results from these studies were similar to many findings in the model plant species *Arabidopsis* and other crops, such as rice and maize (Lopez-Arredondo et al. 2014), suggesting that root system architecture modification might be a conserved mechanism between soybean and other plant species. Despite progress, how some soybean plants exhibit LP tolerance is still largely unclear. It is essential to uncover key genes controlling LP tolerance in soybean, which would enhance our understanding of the main mechanism and allow us to utilize these genes for soybean improvement.

The GWAS has been indicated to be an effective approach in identifying genes associated with complex traits, including those related to LP tolerance (Zhang et al. 2014; Monnot et al. 2021; Yi et al. 2021; Chien et al. 2022; Yang et al. 2023). With this approach, *AtPILS7* was found to contain key variants that can distinguish LP tolerance accessions from sensitive accessions (Yi et al. 2021). *PHOSPHATE TRANSPORTER 1;1* (*AtPHT1;1*) was identified as a key determinant of Pi acquisition in *Arabidopsis* nature accessions (Chien et al. 2022), and 2 LP-associated genes, *ACID PHOSPHATASE 1* (*GmACP1*) (Zhang et al. 2014) and *PHOSPHATE TRANSPORTER TRAFFIC FACILITATOR 1* (G*mPHF1*) (Guo et al. 2022), in soybean were identified, demonstrating the usefulness of this approach to reveal key genes or variants related to Pi efficiency. It is estimated that there are approximately 80 QTLs that are associated with LP tolerance in soybean (https://soybase.org). Therefore, it is necessary to perform a genome-wide search of major QTL genes in soybeans to gain deep knowledge of LP tolerance and identify favorable alleles to facilitate soybean improvement.

In this study, we identified a major QTL on chromosome 19 that was simultaneously associated with 7 LP-induced root morphological traits and Pi efficiency traits across multiple environments through QTL mapping and GWAS. G *GLYCEROPHOSPHORYL DIESTER PHOSPHODIESTERASE2* was identified as the gene underlying the QTL. We further experimentally confirmed the important roles of GmGDPD2 in enhancing LP tolerance and yield traits in soybean. GmGDPD2 physically interacts with GmGA2ox1, while it is regulated by the LP tolerance negative transcription factor GmMyb73. This systematic study revealed a large effect regulatory module, Myb73–GDPD2–GA2ox1, that could be used to design Pi-efficient soybean through marker-assisted breeding or genetic engineering strategies.

## Results

### Identification of a major QTL controlling multiple LP-related traits

**Figure 1.**
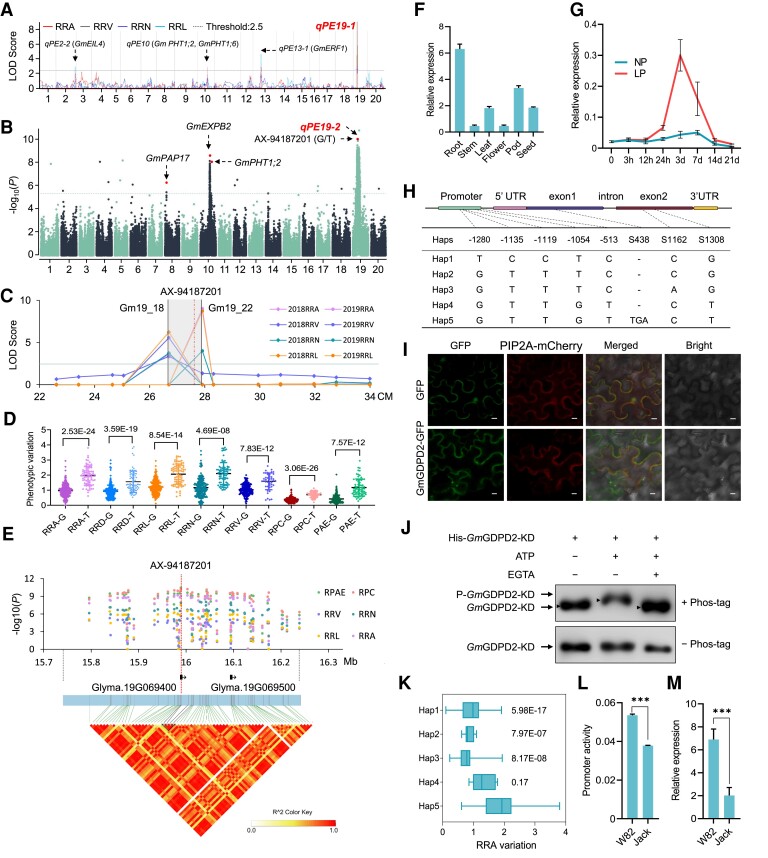
Identification of major QTL *qPE19* and the underlying gene. **A)** Significant QTLs for 4 low P (LP) related root traits identified by QTL mapping based on DW RIL population. PE, phosphate efficiency; LOD score, logarithm of the odds score; RRL, relative root length (RRL); RRA, relative root area; RRN, relative number of hairy roots; RRV, relative root volume. **B)** Significant QTLs for LP-related root trait RRA identified by GWAS analyses and their candidate genes. **C)** Co-localization between *qPE19* and overlapped QTLs controlling 4 LP-related root traits as determined by linkage mapping. **D)** Phenotypic comparisons for 7 LP related root and P efficiency traits between 2 groups divided by the lead SNP AX-94187201 (G/T). RRD, relative root diameter; RPC, relative P concentration; RPAE, relative P absorption efficiency. **E)** The linkage disequilibrium of the genomic region surrounding the lead SNP (AX-94187201) of *qPE19-2* and the 2 genes located in this region. **F)** Expression pattern of Glyma.19G069400 for Williams82 (W82) in 6 tissues under NP condition. **G)** Expression levels of Glyma.19G069400 for W82 in roots after NP and LP treatments for 21 d. **H)** Haplotype analysis of *GmGDPD2.***I)** Subcellular localization of *Gm*GDPD2-GFP fusion protein in *N. benthamiana* leaf cells. Empty GFP was used as a control. PIP2A-mCherry was co-expressed as the PM (plasma membrane) maker. Bar = 10 *μ*m. **J)** In vitro autophosphorylation assays of recombinant proteins His-GmGDPD2-CD (GmGDPD2-CD, 246–564 aa). The phosphorylation reaction was initiated by addition of ATP and inhibited by the Ca^2+^ chelator ethyleneglycoltetraacetic acid (EGTA). Phosphorylation level was detected by immunoblotting using His antibody (1:5000). The Phos-tag SDS-PAGE system was used in subsequent GmGDPD2 phosphorylation assays. **K)** The RRA trait for 5 haplotypes of *GmGDPD2*. Center line, median; box limits, upper and lower quartiles; whiskers, minimum and maximum values. **L)** Promoter activity for Hap5pro and Hap1pro from W82 and Jack, respectively, as determined by luciferase reporter assay. **M)** Expression level comparison of *GmGDPD2* between W82 and Jack by RT-qPCR. Trait values of images F, G, L and M are shown as the mean ± SD, and each line contains 3 individuals. Pairwise multiple comparison test is equivalent to multiple individual *t*-tests between all pairs of groups. ***, *P* < 0.001.

We carried out linkage mapping for 4 LP-related root traits using the DW recombination inbred line (RIL) population (Kong et al. 2018), namely, relative root surface area (RRA), relative root length (RRL), relative number of root tips (RRN), and relative root volume (RRV), and found that these traits exhibited significant genetic variation (Supplementary Fig. S1A; Table S1). The analysis identified 7 LP-related QTL (Fig. 1A; Supplementary Table S2), of which *qPE2-2*, *qPE10*, and *qPE13* were close to the previously identified P efficiency genes *ETHYLENE-INSENSITIVE 3-LIKE 1 PROTEIN* (*GmEIL4*) (Yang et al. 2023), *GmPHT1* (Song et al. 2014; Guo et al. 2022), and *GmERF1* (Wang et al. 2023b), respectively. The QTL *qPE19*-*1* on chromosome 19 (6322262–15891973 bp, based on the Wm82.a4 reference genome) was a QTL that was identified for all 4 LP-related root traits in 2 environments (2018, 2019) and explained 9.04% to 24.06% of the phenotypic variation, demonstrating that it has large effects on the traits.

We next conducted GWASs for the aforementioned 4 LP-related root traits, relative root diameter (RRD), and 2 Pi-associated physiological traits including relative Pi concentration (RPC) and relative Pi absorption efficiency (RPAE), in a diverse collection of 367 (246 *G. max* and 121 *G. soja*) soybean accessions that were collected from 6 ecological regions of soybean cultivation with varying Pi concentrations in soil (http://www.geodata.cn/index.html). All 7 LP-related traits in the panel displayed considerable variation with a nearly normal distribution (Supplementary Fig. S1B; Table S3), indicating that these traits are ideal for GWAS analysis. In addition, these traits showed varying levels of intercorrelation, with RPC-RRA (*r* = 0.95) and RPAE-RRA (*r* = 0.75) pairs exhibiting the top 2 correlations (Supplementary Table S4), suggesting that the LP-related traits are in markedly close genetic relationships and might share a genetic basis in common.

In total, 9 QTL that were significantly associated with the 7 LP-related traits were identified through GWAS (Fig. 1B, Supplementary Fig. S2; Table S5), and the significant SNPs on chromosomes 8, 10, 14, and 17 were physically close to the previously identified P efficiency genes *GmWRKY46*, *GmEXPB2* (*β-EXPANSIN*) and *GmPHT1;2*, *GmETO1*, and *GmEXLB1* (*β-EXPANSIN-LIKE B1*) (Guo et al. 2011, 2022; Kong et al. 2019; Liu et al. 2020, 2022). Importantly, a major QTL, *qPE19*-*2*, was significantly associated with all 7 LP-related traits, with *P* values ranging from 3.73 × 10^−11^ to 2.42 × 10^−6^, and explained 13.3% to 43.2% of the phenotypic variation (Supplementary Table S5). The SNP AX-94187201 (chr19: 15,989,213, based on the Wm82.a4 genome) was among the top SNPs of *qPE19*-*2* (Supplementary Table S5), and it was located within the QTL interval of *qPE19*-*1* that was detected by the RIL population (Fig. 1C), indicating that QTL *qPE19*-*1* and *qPE19*-*2*, as revealed by linkage mapping and GWAS, were likely the same locus associated with the LP-related traits. AX-94187201 was significant for 7 LP-related traits, and 2 AX-94187201-derived haplotypes (G/T) could distinguish LP-tolerant and LP-sensitive accessions in the association panel. Briefly, accessions carrying allele T were, on average, 63% to 180% more abundant in the 7 LP-related traits than those carrying allele G (*P* = 4.7 × 10^−8^–7.8 × 10^−26^) (Fig. 1D), indicating that AX-94187201 is highly associated with *qPE19*-*2* in controlling these traits.

### GmGDPD2 controls qPE19

Considering that the LD (decay to 0.2) for our 367 soybean accessions was 226.51 kb (Supplementary Fig. S2H), we scanned a 500-kb interval centered around the SNP AX-94187201 and identified 2 predicted genes (Glyma.19G069400 and Glyma.19G069500) (Fig. 1E; Supplementary Fig. S3). RT-qPCR and RNA-Seq data (https://soyatlas.venanciogroup.uenf.br/) consistently showed that *Glyma.19G069400* was expressed in all examined tissues (root, stem, leaf, flower, pod, and seed) of the soybean cultivar W82, with predominant expression in roots (Fig. 1F, Supplementary Fig. S4A). A time-course examination demonstrated that *Glyma.19G069400* was highly induced in LP-treated roots, with the maximum difference seen at 3 d, compared with those under NP conditions (Fig. 1G). In contrast, *Glyma.19G069500* was expressed at quite low levels in all of the investigated tissues (Supplementary Fig. S4, B and C), and the expression of Glyma.19G069500 in NP and LP conditions was undetectable; it was not inducible by LP conditions. High expression of Glyma.19G069400 in roots was further validated via a GUS histochemical assay with the 2.0-kb promoter fragment to drive the GUS gene, where the transgenic hairy roots were dark blue and blue under LP and NP conditions, respectively (Supplementary Fig. S5). Importantly, we found that AX-94187201 (S1308) is located within the coding DNA sequence (CDS) of Glyma.19G069400 (Fig. 1H). These results strongly indicated that Glyma.19G069400 is the most promising candidate gene of *qPE19*.

Glyma.19G069400 encodes a 599-amino acid glycerophosphoryl diester phosphodiesterase (GDPD) carrying a wall-associated receptor kinase galacturonan-binding domain (24–133) at the N-terminus and a Pkinase_Tyr domain (287–556) close to the C-terminus (Supplementary Fig. S6). It was located in the plasmatic membrane (Fig. 1I). Glyma.19G069400 has 48.9% identity with *Arabidopsis GDPDL2* (Cheng et al. 2011); therefore, we designated it *GmGDPD2*. The His-tagged Pkinase_Tyr domain of GmGDPD2 (His-GmGDPD2-KD) was expressed in *E. coli* BL21 (DE3) and purified by affinity purification with Ni-NTA resin for kinase activity analysis. The recombinant protein was confirmed by western blot using an anti-His monoclonal antibody combined with Coomassie blue staining (Supplementary Fig. S7). The addition of the Ca^2+^ chelator ethyleneglycoltetraacetic acid (EGTA) to the phosphorylation system can significantly reduce the upper band of GmGDPD2-KD, and P-GmGDPD2-KD represents a phosphorylated form of GmGDPD2-KD. This result proved that GmGDPD2 has kinase autophosphorylation (Fig. 1J).

### Natural variation in ***GmGDPD2*** is associated with LP-related traits

To determine the variation in *GmGDPD2* responsible for LP adaptation, we analyzed the allelic variation in the panel, including 1500 bp upstream of the start codon (ATG), CDS, and 200-bp 3′ untranslated region (UTR). A total of 8 variants (minor allelic frequency >5%, Fig. 1H) were identified, including 5 SNPs in the promoter region, 1 indel (insertion and deletion) (S438), and 2 nonsynonymous SNPs (S1162 and S1308) in the CDS. The 8 variants classified the panel into 5 haplotypes (Hap). Statistically, Hap5-carrying accessions had significantly higher RRAs than those carrying Hap1–Hap4 (Fig. 1K). For example, accessions with Hap5 had RRA ratio values that were 82.2% to 87.5% higher than those carrying Hap1 (*P* = 5.98 × 10^−17^), Hap2 (*P* = 7.97 × 10^−7^), Hap3 (*P* = 8.17 × 10^−8^), and Hap4 (*P* = 0.17) (Fig. 1K). In particular, the indel at 438 (TGA/-) can be used to distinguish between high-RRA Hap5 from relatively low-RRA Hap1–Hap4, making it one of the key variants. We therefore designated Hap5 as the optimal haplotype of *GmGDPD2* that can enhance LP tolerance and Pi-use efficiency. We re-sequenced the *GmGDPD2* gene in 9 soybean accessions with 6 Hap5 and 3 Hap1 and found that the 3-bp deletion (TGA at S438) and the SNP (A/C at 1313) was linked with in the CDS region (Supplementary Fig. S8). The TGA deletion in Hap1 led to a deletion of Asp (D), and the SNP variation in Hap1 changed the Ile (I) to Leu (L) (Supplementary Fig. S9). Structure prediction of Hap1 and Hap 5 indicated that the 2 variations may greatly change the protein structure of GmGDPD2 (Supplementary Fig. S10). We developed KASP (Kompetitive Allele Specific PCR) markers based on the TGA deletion (S438) and confirmed that the indel could effectively distinguish Hap5 from other Haps in a panel of 45 lines that were randomly selected from the 367 soybean accessions. Interestingly, we further examined 34 soybean cultivars from the Huang-Huai-Hai Area of China and identified 6 of them harboring Hap5, which indicates they are highly Pi-efficient cultivars (Supplementary Table S6).

In addition, we asked whether the variation occurring in the promoter region affects gene expression. The luciferase (LUC) reporter assay showed 1.42-fold higher (*P* = 8.34 × 10^−5^) promoter activity for Hap5pro from W82 (LP tolerant) than Hap1pro from Jack (LP sensitive) (Fig. 1L). In support of this result, the expression of *GmGDPD2* was 3.41 times higher in W82 than in Jack after LP treatment for 3 d (Fig. 1M). These results further support that *GmGDPD2* is highly associated with LP tolerance and that its expression may contribute to high LP tolerance in Hap5 accessions.

### GmGDPD2 promotes root growth and Pi uptake

**Figure 2.**
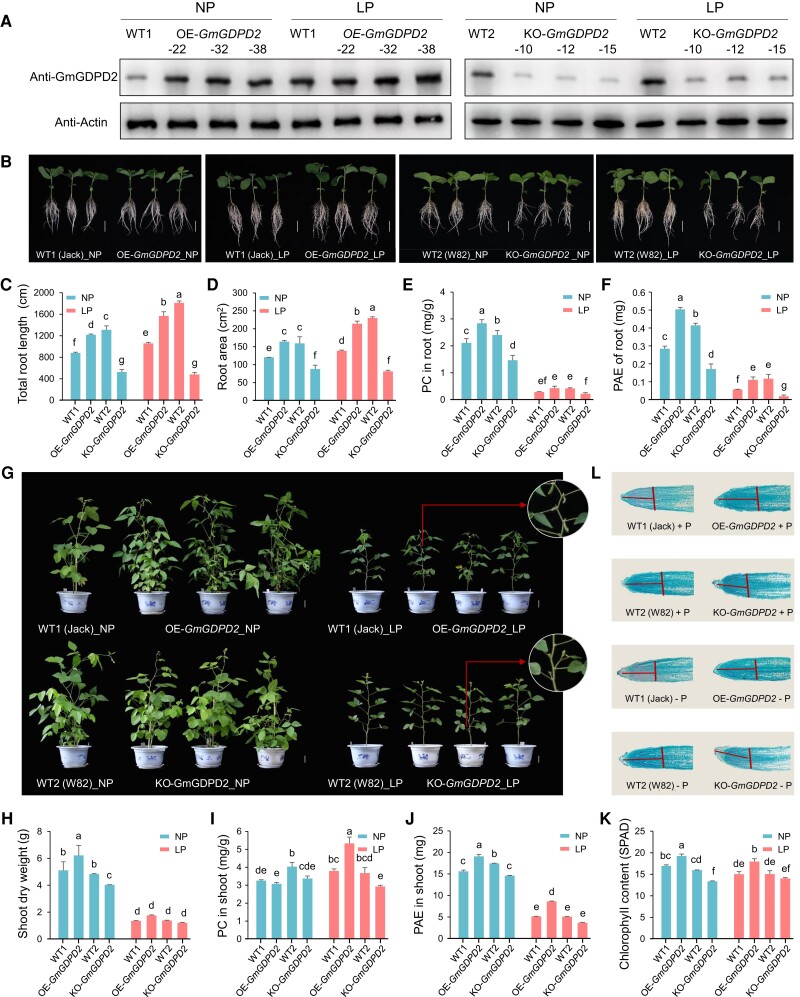
Comparison between *GmGDPD2* overexpression or knockout roots with corresponding wild types. **A)** Protein accumulation of GmGDPD2 between GmGDPD2 overexpression or knockout roots with corresponding wild types under NP and LP conditions for 7 d. Three plants were selected for protein extraction. **B–F)** Root traits between the *GmGDPD2* overexpression and knockout plants with the wild types (WT1, Jack) and (WT2, W82) under NP or LP conditions for 7 d. LP, low P supply (5 *μ*M, Pi); NP, normal P supply (500 *μ*M, Pi). Bars = 5 cm. PC, Pi concentration; PAE, Pi absorption efficiency. **G–K)** Shoot traits between the *GmGDPD2* overexpression and knockout plants with the wild types (WT1) and (WT2) under NP (+P) or LP (−P) conditions in plot for 45 d. **L)** Micrographs of longitudinally sectioned transgenic and wild-type roots under NP or LP conditions for 7 d. The phenotypic statistics of *GmGDPD2* overexpression and knockout plants were based on 3 different overexpression lines and 3 different knockout lines, respectively, and the phenotype of each line for each treatment was evaluated with 3 plants. Bars = 5 cm. Trait values of images C–F and H–K are shown as the mean ± SD. Means with different letters are significantly different (one-way ANOVA, Duncan, *P* ≤ 0.05).

To investigate the biological effects of GmGDPD2 on LP tolerance, we generated 3 *GmGDPD2* (Hap5) overexpression (OE-*GmGDPD2*) lines in the Hap1 background (Jack, LP sensitive; wild-type 1, WT1) (Supplementary Fig. S11A) and 3 *GmGDPD2* knockout (KO-*GmGDPD2*) lines in the Hap5 background (W82, wild-type 2, WT2) (Supplementary Fig. S11B). The protein expression levels for 3 OE-*GmGDPD2* roots were higher than those of WT1 roots under both NP and LP conditions; in contrast, 3 KO-*GmGDPD2* lines showed lower protein expression levels than WT2 under both NP and LP conditions (Fig. 2A). Moreover, the protein expression levels for both WT1 and OE-*GmGDPD2* lines were higher in the LP condition than that in the NP condition (Fig. 2A), which indicated that GmGDPD2 is induced in the LP condition. The phenotypes were evaluated in hydroponic culture with either NP or LP supply (Fig. 2). We observed that W82 (WT2) plants had significantly higher values for root architecture traits (RL and RA) and Pi efficiency-related traits (PC and PAE) than Jack plants (WT1) regardless of Pi supply at the seedling stage (Fig. 2B) and the reproductive stage (Fig. 2G), supporting the haplotype analysis that Hap5-carrying plants outperformed in these traits relative to Hap1-carrying accessions. Specifically, lines overexpressing *GmGDPD2* were superior in these root traits with 35.2% to 55.1% higher values than WT1 under NP or LP conditions (Fig. 2, C and D, Supplementary Fig. S12, A and B). The enlarged root system also greatly enhanced PC by 35.2% (*P* = 7.75 × 10^−4^) and PAE by 50% (*P* = 1.96 × 10^−3^) in OE-*GmGDPD2* roots compared with WT1 roots under NP conditions, and the increases were greater with 64.3% (*P* = 2.15 × 10^−3^) for PC and 92.3% (*P* = 9.52 × 10^−4^) for root PAE under LP conditions (Fig. 2, E and F, Supplementary Fig. S12, C and D). In contrast, the knockout of *GmGDPD2* results in 35.3% to 59.9% reductions in these root architecture traits, 38.3% (*P* = 1.81 × 10^−2^–8.97 × 10^−4^) in PC, and 60.9% (*P* = 8.64 × 10^−4^–6.61 × 10^−5^) in root PAE (Fig. 2, B to E, Supplementary Fig. S12); similar increasing patterns were also observed in shoots for plants grown in pots (Fig. 2, G to J, Supplementary Fig. S13). These results suggest that GmGDPD2 enhances LP tolerance by promoting the development of root morphological traits and Pi efficiency traits.

We also investigated the exudation of 2 organic acids, oxalic acid and malic acid, that might be associated with P mobilization and uptake (Hocking 2001; Peng et al. 2018). We observed that overexpression of *GmGDPD2* enhanced the exudation of 2 organic acids (Supplementary Fig. S14). For example, OE-*GmGDPD2* roots exudated 8.99% to 128.00% more oxalic acid and 1.79% to 89.30% more malic acid than WT1 under NP or LP conditions, while oxalic acid and malic acid were 21.24% to 63.32% and 38.46% to 59.91% less, respectively, in KO-*GmGDPD2* roots than in WT2 roots under LP conditions.

We further asked how GmGDPD2 affects root growth and performed a microscopic examination of longitudinally sectioned roots. After examination, we observed a significantly increased number of smaller cells with a denser arrangement in the elongation and meristematic areas in root caps of OE-*GmGDPD2* root tips than in those of WT1 in both NP and LP conditions (Fig. 2L). These findings provide strong evidence that GmGDPD2 is involved in root growth, likely through the promotion of cell division in root tips, which is partially independent of P supply. Conversely, the root tips in KO-*GmGDPD2* lines are shorter with fewer cells in the areas than the corresponding wild type.

### GmGDPD2 affects multiple genes to enhance P efficiency and root growth

To gain insight into the downstream biological processes of GmGDPD2, we compared root transcriptomes in OE-*GmGDPD2* and KO-*GmGDPD2* plants with those of the respective wild types. The analysis identified 1,377 differentially expressed genes (DEGs) between OE-*GmGDPD2* and WT1 plants and 5,240 DEGs between KO-*GmGDPD2* and WT2 plants under NP conditions, and in parallel, more genes, namely 4,982 and 7,483 DEGs, in the comparisons were identified accordingly under LP stress. Venn diagram analyses consolidated the numbers to 518 and 1,819 DEGs shared by NP- and LP-treated transgenic roots, respectively (Supplementary Fig. S15A). GO term enrichment analysis revealed that biological processes associated with photosynthesis and photosystem were the most enriched (Supplementary Fig. S15B). The chlorophyll measurement in the greenhouse supported the result, where we demonstrated that the chlorophyll content significantly increased by 13.6% to 19.5% in OE-*GmGDPD2* plants but decreased by 6.7% to 16.3% in KO-*GmGDPD2* plants when compared with the respective wild types (Fig. 2K). In addition, GO terms associated with the regulation of protein dephosphorylation, response to red light, and pentose phosphate shunt were commonly enriched, suggesting that GmGDPD2 regulates LP-related traits by affecting these biological processes.

To connect *GmGDPD2* with current knowledge in LP tolerance, we investigated the expression of 7 previously reported genes between transgenic roots and WT roots (Supplementary Fig. S16), although some of the GO terms were not enriched. RT-qPCR identified several differentially regulated genes, including genes encoding acid phosphatase *GmACP1* and *ACID PHOSPHATASE 2* (*GmACP2*) (Zhang et al. 2014, 2017), purple acid phosphatase *PURPLE ACID PHOSPHATASE 17* (*GmPAP17*) (Xu et al. 2022); genes that govern LP-induced root meristem and root development, *LOW PHOSPHATE ROOT2* (*GmLPR2*) (Xu et al. 2020), *RGF1 INSENSITIVE 1* (*GmRGI1*) (Shinohara et al. 2016), *EXPANSIN-A7* (*GmEXPA7*) (Lin et al. 2011), oxidative stress tolerance *OXIDATIVE STRESS 3* (*GmOXS3*) (Blanvillain et al. 2009), and growth repressor genes *GmDELLAs*; Pi transporter *PHOSPHATE TRANSPORTER 1;7* (*GmPT7*) (Chen et al. 2019). Thus, we concluded that GmGDPD2 likely acts upstream of these genes in the pathway conferring LP tolerance.

### *GmGDPD2* is negatively regulated by the LP tolerance regulator GmMyb73

**Figure 3.**
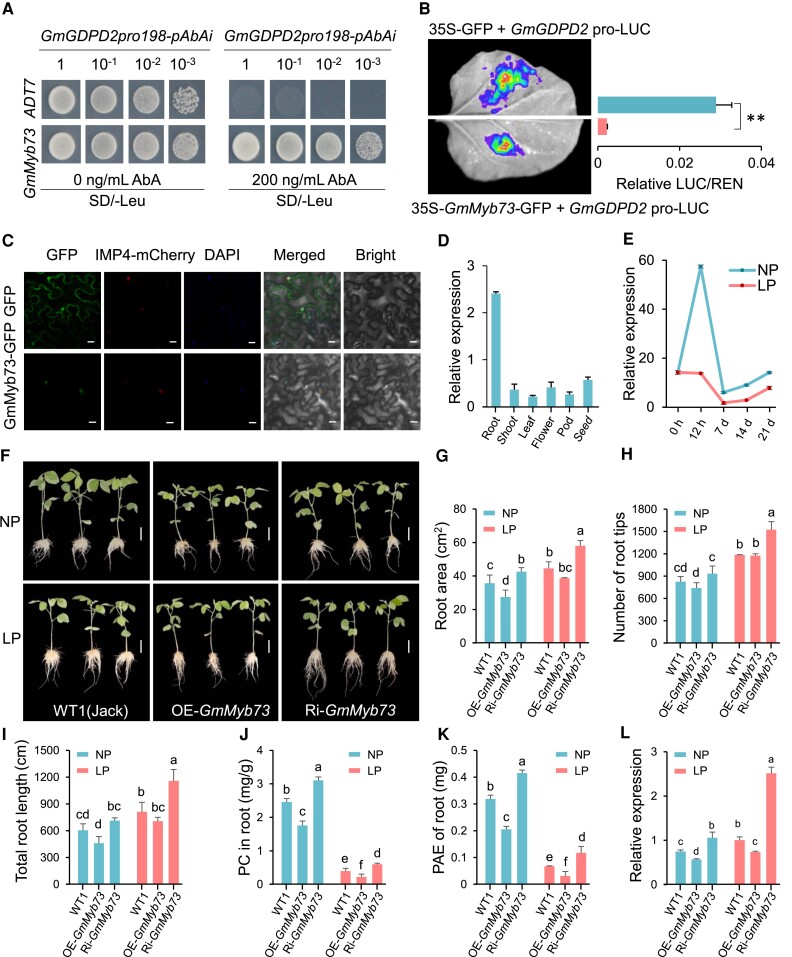
*GmGDPD2* is negatively regulated by LP tolerance regulator GmMyb73. **A)** Yeast one hybrid identified GmMyb73 binding to the promoter of *GmGDPD2*. **B)** Luciferase (LUC) reporter assay confirmed the interaction between GmMyb73 and GmGDPD2pro in *N. benthamiana* leaves and the LUC activities driven by *GmGDPD2* promoter influenced by the presence or absence of GmMyb73. **: *P* < 0.01. **C)** Subcellular localization of GmMyb73-GFP driven by the 35S promoter in *N. benthamiana* leaves. Empty GFP was used as a control. DAPI (4′,6-diamidino-2-phenylindole) specially stained cell nuclei. IMP4-mCherry was co-expressed as the nuclei maker. Bar = 20 *μ*m. **D)** Expression levels of *GmMyb73* for W82 in 6 tissues under NP condition. **E)** Expression level comparison of *GmMyb73* in W82 roots under NP and LP conditions. **F)** Comparison of soybean hairy roots for WT1, overexpressing *GmMyb73* (OE-*GmMyb73*) plants and silencing *GmMyb73* (Ri-*GmMyb73*) plants after NP or LP treatments for 10 d. Bars = 5 cm. **G–K)** Comparison of root and P efficiency traits (root areas (G), the number of root tips (H), total root length (I), Pi concentration in roots (J), and root PAE (K)) for WT1 and *GmMyb73* transgenic hairy roots after NP or LP treatments for 10 d. The phenotype of each line for each treatment was evaluated with 3 plants. **L)** Expression level comparison of *GmGDPD2* in WT1 and *GmMyb73* transgenic hairy roots after NP or LP treatments for 10 d. LP, low P supply (5 *μ*M, Pi); NP, normal P supply (500 *μ*M, Pi). Trait values of images of B, D, and G–L are shown as the mean ± SD. Means with different letters are significantly different (one-way ANOVA, Duncan, *P* ≤ 0.05).

The above results demonstrated that GmGDPD2 is an important LP tolerance factor with strong LP induction. Yeast one-hybridization (Y1H) with a serial dilution of yeast solution identified that GmGDPD2 interacted with the R2R3-type transcription factor GmMyb73 (Glyma.01G190100) (Fig. 3A). This interaction was confirmed by a LUC complementation assay in *Nicotiana benthamiana* leaves (Fig. 3B). Interestingly, we observed that the signal strength from *GFP*-*GmMyb73*-*GmGDPD2pro*-LUC was approximately 13-fold lower than that from *GFP*-*GmGDPD2pro*-LUC in leaves (*P* = 1.21 × 10^−6^) (Fig. 3B), suggesting that the expression of *LUC* driven by *GmGDPD2pro* was greatly suppressed by *GmMyb73*. These results indicate that *GmGDPD2* may be negatively regulated by GmMyb73.

GmMyb73 localized to the nucleus, as visualized by a protein GFP assay (Fig. 3C). *GmMyb73* was primarily expressed in roots, and the expression levels were markedly downregulated in LP-stressed roots compared with those in NP roots during a time-course gene expression investigation for 21 d (Fig. 3, D and E), implying an important role in soybean roots that might negatively respond to LP stress. To determine its function, we overexpressed or interfered with *GmMyb73* in Jack using modified soybean hairy root transformation (Zhang et al. 2014). Phenotypic investigation (Fig. 3F) revealed that overexpressing *GmMyb73* overall significantly decreased root morphological traits (RL, RN, and RA) and Pi efficiency-related traits (PC and PAE) (*P* = 1.78 × 10^−2^–5.54 × 10^−5^); in contrast, these traits were greatly enhanced in the RNAi plants to varying degrees (*P* = 3.50 × 10^−2^–6.34 × 10^−5^) (Fig. 3, G to K). This result functionally demonstrates that GmMyb73 negatively regulates the LP tolerance in accordance with the expression analysis (Fig. 3, B and E). Furthermore, we verified significant downregulation (*P* = 9.88 × 10^−4^, 3.54 × 10^−3^) in the expression of *GmGDPD2* in OE-*GmMyb73* roots (Fig. 3L) and upregulation (*P* = 1.62 × 10^−2^, 7.99 × 10^−5^) in RNAi-*GmMyb73* roots in both NP and LP conditions, further confirming that GmMyb73 negatively regulates *GmGDPD2*. These results clearly demonstrate that GmMyb73 negatively regulates *GmGDPD2* by directly targeting its promoter, which likely regulates LP tolerance in soybean.

### GmGDPD2 physically interacts with GmGA2ox1

**Figure 4.**
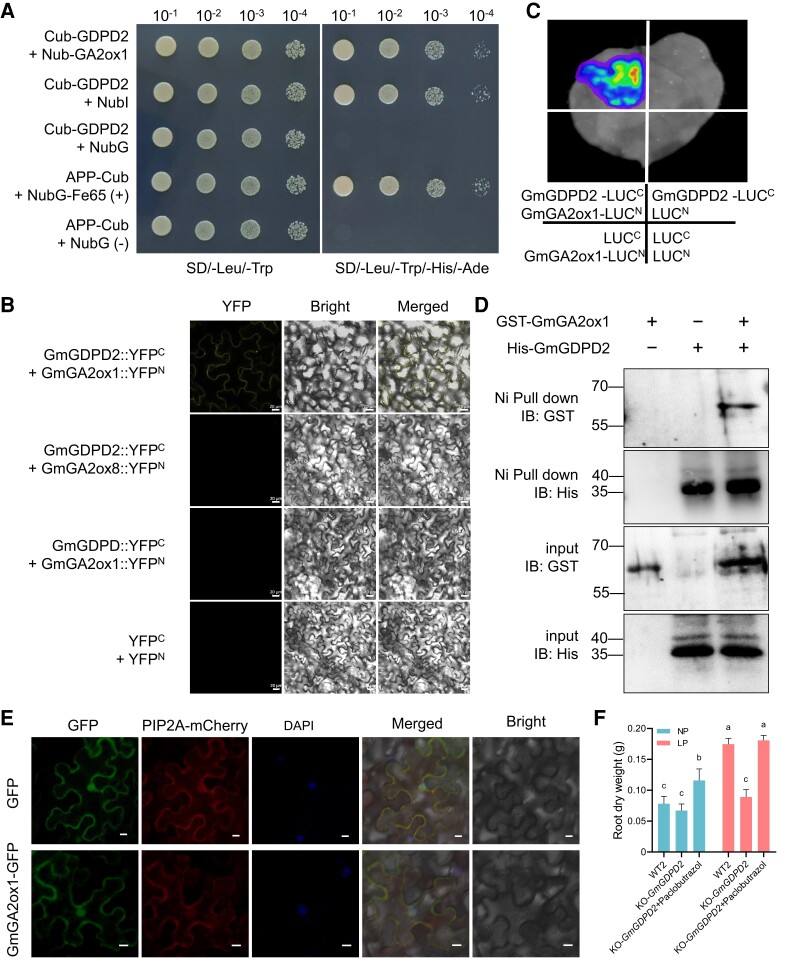
GmGDPD2 physically interacts with GmGA2ox1. **A)** Yeast split-ubiquitin assay indicates an interaction between GmGDPD2 and GmGA2ox1. Cub-GmGDPD2 + NubI and Cub-GmGDPD2 + NubG were used as positive and negative controls, respectively. APP-Cub + NubG-Fe65 and APP-Cub + NubG were provided with the commercial yeast split-ubiquitin assay system and used as positive and negative controls, respectively. **B)** Bimolecular fluorescence complementation analysis of the GmGDPD2–GmGA2ox1 interaction in *N. benthamiana* leaves. Fluorescence signals were monitored using confocal microscopy; images were taken 48 h after transformation. Bar 20 *μ*m. The images shown are representative of at least 3 independent experiments. GmGA2ox8 (Glyma.15G093900.1) was a member of the same protein family as GmGA2ox1, GmGDPD (Glyma.02G100400.1) was a member of the same protein family as GmGDPD2. **C)** For luciferase complementation imaging assays, *N. benthamiana* leaves were transformed with pairwise combinations of GmGDPD2-LUC^C^ with GmGA2ox1-LUC^N^, GmGDPD2-LUC^C^ with LUC^N^, GmGA2ox1-LUC^N^ with LUC^C^ and LUC^C^ with LUC^N^. The light spectrum bar indicates the signal intensity. nLUC and cLUC indicate the LUC N terminus and C terminus, respectively. **D)** GST-GmGA2ox1 pulled down His-GmGDPD2 in an in vitro pull-down assay. GST-GmGA2ox1 was detected with an anti-GST antibody. His-GmGDPD2 was detected with an anti-His antibody. Molecular mass markers are shown (kDa). **E)** Subcellular localization of GmGA2ox1 in *N. benthamiana* leaves. Empty GFP was used as a control. DAPI (4′,6-diamidino-2-phenylindole) specially stained cell nuclei. PIP2A-mCherry was co-expressed as the PM (plasma membrane) maker. IMP4-mCherry was co-expressed as the nuclei maker. Bar = 10 *μ*m. **F)** Root dry weight of WT2, KO-*GmGDPD2* and KO-*GmGDPD2* with Paclobutrazol treatment under NP and LP condition for 8 d. Paclobutrazol concentration: 30 mg/L. KO-*GmGDPD2* represents 3 Gm*GDPD2* knockout lines, the phenotype of each Gm*GDPD2* line for each treatment was evaluated with one plant, WT2 was evaluated with 3 plants. Trait values are shown as the mean ± SD. Means with different letters are significantly different (one-way ANOVA, Duncan, *P* ≤ 0.05).

Through yeast two-hybridization (Y2H) assay, Glyma.13G259400 was identified to physically interact with GmGDPD2 (Fig. 4A). The interaction was further confirmed with an in vitro pull-down assay and in vivo bimolecular fluorescence complementation (BiFC) and LUC complementation imaging assays in *N. benthamiana* leaves, and the interaction occurred in the plasma membrane (Fig. 4, B to D). Glyma.13G259400 encodes a gibberellin 2-dioxygenase, with 60% identity to AT1G78440 (GA2ox1), which might be associated with the homeostasis of bioactive gibberellins (Thomas et al. 1999). Therefore, we designated Glyma.13G259400 as *GmGA2ox1*. This interaction implies that GmGA2ox1 is likely involved in the GmGDPD2 pathway conferring LP tolerance. GmGA2ox1 localized on the nucleus and cellular membrane as visualized by protein GFP assay (Fig. 4E). *GmGA2ox1* showed a moderate expression level in roots relative to other selected tissues (Supplementary Fig. S17A), and the expression was significantly increased in OE-*GmGDPD2* roots and significantly decreased in KO-*GmGDPD2* roots compared with the respective wild types under both NP and LP conditions (Supplementary Fig. S17B), suggesting that the GA biosynthesis pathway might be involved in the GmGDPD2*-*associated root morphological and plant height changes (Fig. 4A, Supplementary Figs. S4F and S18). To verify this, we applied paclobutrazol [a gibberellin (GA) inhibitor] to transgenic soybean plants. The exogenous application of paclobutrazol restored the phenotypic values for RA, RL, RV and plant height in KO-*GmGDPD2* plants to the levels in WT2 under both NP and LP conditions (Supplementary Fig. S18). These results suggest that the GmGA2ox1-associated GA pathway is likely involved in GmGDPD2-regulated LP tolerance.

### GmGA2ox1 regulates root architecture traits and increases Pi efficiency

**Figure 5.**
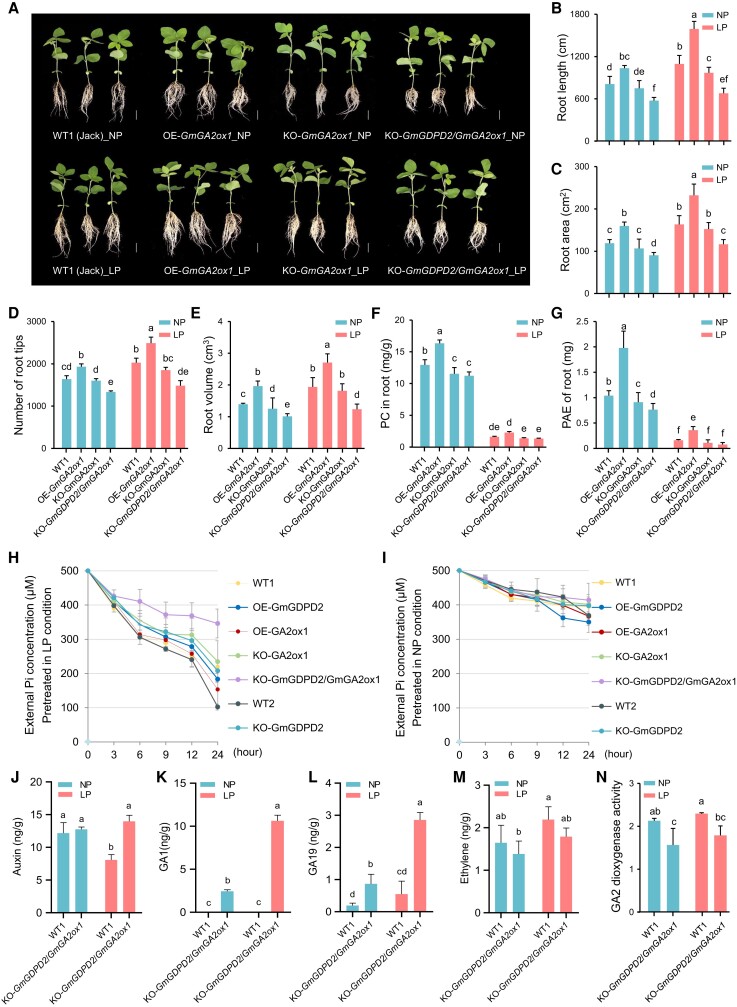
Phenotypic comparison between *GmGA2ox1* transgenic root traits and the wild type. **A)** Morphologic illustration of WT1, *GmGA2ox1* overexpression (OE-*GmGA2ox1*) plants, knockout (KO-*GmGA2ox1*) plants and double knockout (KO-*GmGA2ox1*/*GmGA2ox1*) plants after NP or LP treatments for 10 d. Bars = 5 cm. **B–G)** Comparisons of root and P efficiency traits between transgenic plants and the wild-type plants after NP or LP treatments for 10 d. **H,I)** Pi-uptake rate measurement. Seedlings with unfolded cotyledons were pretreated in NP and LP condition for days, then transferred to deionized water for 22 h, and then transferred to black plastic cup in NP condition. **J–M)** Phytohormones” changes in roots of KO-*GmGA2ox1*/*GmGA2ox1* plants after NP or LP conditions for 14 d, including auxin, GAs, and ethylene. **N)** GA2 dioxygenase activity in roots of KO-*GmGA2ox1*/*GmGA2ox1* plants after NP or LP treatments for 7 d. LP, low P supply (5 *μ*M, Pi); NP, normal P supply (500 *μ*M, Pi). The phenotypic statistics of OE-*GmGA2ox1*, KO-*GmGA2ox1*, and KO-*GmGDPD2/GmGA2ox1* were based on 3 different overexpression lines, 3 different knockout lines, and 2 different double knock lines, respectively, and the phenotype of each line for each treatment was evaluated with 3 plants. Trait values of image B–N are shown as the mean ± SD. Means with different letters are significantly different (one-way ANOVA, Duncan, *P* ≤ 0.05).

With the confirmed interaction, we next assessed whether GmGA2ox1 is involved in the response to LP stress. Three independent transgenic soybean plants overexpressing *GmGA2ox1* (OE-*GmGA2ox1*) and knockout mutants (KO-*GmGA2ox1*) in the Jack background (WT1) were obtained and evaluated in hydroponic culture experiments for phenotypic investigation (Fig. 5A; Supplementary Fig. S19). Overall, OE-*GmGA2ox1* plants exhibited enhanced root architecture traits in both NP and LP conditions, similar to OE-*GDPD2* plants (Fig. 5A). Specifically, overexpression of *GmGA2ox1* significantly increased RL (28.6%), RA (34.2%), RN (18.9%), RV (41.0%), PC (26.4%), and PAE (90.4%) in NP conditions (Fig. 5, B to G, Supplementary Fig. S20). In contrast, the increasing degrees were much greater under LP conditions.

Although the difference was not statistically significant, KO-*GmGA2ox1* mutants showed measurable decreases in these root traits (RL, RA, RN, and RV). Similar to the root traits, root PC or PAE in KO-*GmGA2ox1* roots were significantly lower (10.8% to 12.6%, *P* = 2.23 × 10^−2^–4.91 × 10^−2^) than those in WT1 under NP conditions **(**Fig. 5, F and G, Supplementary Fig. S20); unexpectedly, LP stress greatly decreased both traits (15.3% to 32.8%, *P* = 2.47 × 10^−3^–6.89 × 10^−3^) relative to those in WT1. These results indicate that GmGA2ox1 can enhance root architecture traits and P efficiency-related traits.

### *GmGDPD2 GmGA2ox1* double mutants further reduced the root traits

Given the potential for physical plant interactions between GmGDPD2 and GmGA2ox1 and the consistent effects on root architecture traits, we asked how these traits were displayed when both genes were mutated. To do this, we crossed KO*-GmGDPD2* and KO*-GA2ox1* plants to generate plants homozygous for both mutations (KO-*GmGDPD2*/*GmGA2ox1*) (Fig. 5A). We found that mutating 2 genes caused a greater reduction (18.5% to 37.9%) in root traits (RL, RA, RV, and RN) and P efficiency traits (11.3% to 51.5%) than WT1 or either of the knockout mutants (Fig. 5, B to G). The shoots of the KO-*GmGDPD2*/*GmGA2ox1* lines were obviously smaller than those of KO*-GmGDPD2*, KO*-GmGA2ox1*, and the wild types (WT1, WT2) under both NP and LP conditions for 8 d (Supplementary Fig. S21). Consistent with WT1, KO-*GmGDPD2*/*GmGA2ox1* showed a significant decrease in root architecture and Pi efficiency traits relative to either the *GmGDPD2* or *GmGA2ox1* mutant under NP and LP conditions. These results suggest that GmGDPD2 and GmGA2ox1 function synergistically in the regulation of LP tolerance by modifying root architecture traits.

We further compared the time-course changes in the Pi-uptake rate among the transgenic lines. The results showed that all the lines pretreated in LP conditions showed a higher Pi-uptake rate than those pretreated in NP conditions (Fig. 5, H and I, Supplementary Fig. S22). In the LP-NP switch, the OE-*GmGDPD2* and OE-*GmGA2ox1* lines took up 13.01% and 23.73% more Pi than WT1, while the KO lines took up 5.26% and 26.61% less Pi than the respective wild types. As expected, the double knockout line KO-*GmGDPD2*/*GmGA2ox1* took up 45.15% and 42.10% less Pi than WT1 and WT2, respectively, which was much less than the single gene mutants (Fig. 5H, Supplementary Fig. S22A). In contrast, in the NP–NP switch, all the transgenic lines showed Pi-uptake trends similar to those of the respective wild types (Fig. 5I, Supplementary Fig. S22B). These results suggest that GmGDPD2 and GmGA2ox1 play a synergistic role in enhancing Pi uptake.

GA is an essential hormone for root growth and development (Ubeda-Tomás et al. 2008) and plant adaptation to low Pi availability (Jiang et al. 2007; Zhang et al. 2019b; Gualano et al. 2021). Therefore, we next measured the levels of 4 phytohormones (auxin, bioactive GA1, and GA19, ethylene) in KO-G*mGDPD2*/*GmGA2ox1* under NP or LP conditions. The results indicate that the concentrations of auxin, bioactive GA1, and GA19 were significantly increased (*P* = 1.93 × 10^−2^–1.55 × 10^−5^) in KO-G*mGDPD2*/*GmGA2ox1* roots compared with in WT1 roots under NP conditions, and the increases were more dramatic (*P* = 1.17 × 10^−3^–9.04 × 10^−6^) under LP conditions (Fig. 5, J to L, Supplementary Fig. S23, A to C). In contrast, the concentration of ethylene in KO-G*mGDPD2*/*GmGA2ox1* roots was reduced under both NP and LP conditions (Fig. 5M, Supplementary Fig. S23D), and the activity of GA2 dioxygenase in KO-*GmGDPD2/GmGA2ox1* roots was significantly reduced under both NP and LP conditions (*P* = 3.10 × 10^−3^–4.86 × 10^−3^) (Fig. 5N, Supplementary Fig. S24). These results suggest that GmGDPD2–GmGA2ox1 might regulate the root architecture traits and Pi use efficiency traits by influencing auxin and GA biosynthesis pathways.

### GmGDPD2 increases yield-related traits

**Figure 6.**
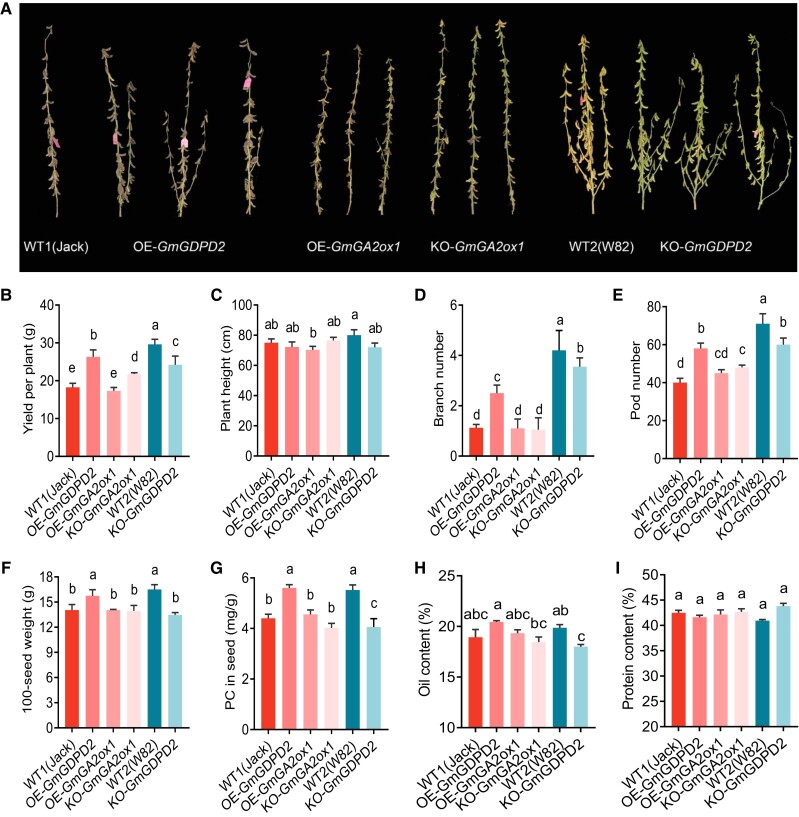
Phenotypic comparison between the *GmGDPD2* and *GmGA2ox1* transgenic plants with the respective wild types after maturation in the greenhouse. **A)** Morphological comparison of the mature transgenic and wild type plants. **B–I)** Comparisons of agronomic traits (yield per plant, plant height, branch number, pod number, and 100-seed weight) and seed quality traits (PC, oil content, and protein content) between transgenic and wild-type plants. The phenotypic statistics of OE-*GmGDPD2*, KO-*GmGDPD2*, OE-*GmGA2ox1*, and KO-*GmGA2ox1* were based on 3 Gm*GDPD2* overexpression, 3 Gm*GDPD2* knockout, 3 Gm*GA2ox1* overexpression, and 3 Gm*GA2ox1* knockout lines, respectively, and the phenotype of each line for each treatment was evaluated with 3 plants. Trait values are shown as the mean ± SD. Means with different letters are significantly different (one-way ANOVA, Duncan, *P* ≤ 0.05).

To better evaluate the breeding value of *GmGDPD2*, we grew the transgenic plants in the field and investigated the effects of *GmGDPD2* and *GmGA2ox1* expression on agronomic traits after maturation (Fig. 6A). The plants overexpressing *GmGDPD2* had higher yield (35.1%, *P* = 2.25 × 10^−3^) and yield-related traits, including branch number (121%, *P* = 6.09 × 10^−3^), pod number (25.0%, *P* = 6.97 × 10^−3^), and 100-seed weight (38.10%, *P* = 2.29 × 10^−3^) than WT1 (Fig. 6, A and B, D to F, Supplementary Fig. S25, A to H). The knockout of *GmGDPD2* reduced these traits and plant height (Fig. 6, A to F). Consistent with the changing patterns in Pi efficiency and associated traits (Fig. 2), the overexpression of *GmGDPD2* significantly increased Pi concentration by 27.3% (*P* = 4.91 × 10^−2^), while the knockout mutant showed a measurable decrease of 26.4% (*P* = 3.06 × 10^−3^) in mature seeds when compared with the respective wild types (Fig. 6G). In addition, OE-*GmGDPD2* seeds contained higher oil content (absolute value = 1.53%, *P* = 2.78 × 10^−2^), while KO-*GmGDPD2* seeds contained lower oil content (absolute value = 1.87%, *P* = 1.05 × 10^−3^) than the respective wild types (Fig. 6H). Accordingly, the protein content was lower in OE-*GmGDPD2* (absolute value = 0.86%, *P* = 0.08) and significantly higher in KO-*GmGDPD2* (absolute value = 2.88%, *P* = 1.03 × 10^−3^) than in the respective wild types (Fig. 6I). In contrast to OE-*GmGDPD2* plants, OE-*GmGA2ox1* or KO-*GmGA2ox1* plants did not show changes in these quality traits. These findings further validate the crucial roles of GmGDPD2 in regulating Pi efficiency at the maturation stages, thereby significantly improving both yield and associated traits.

## Discussion

### *GmGDPD2* is a potential target to facilitate both Pi efficiency and yield improvement

Our study uncovered a major QTL gene, *GmGDPD2*, through linkage and high-resolution GWAS and confirmed the primary role of its product in improving LP-related traits and yield traits. This QTL was identified in a previous linkage mapping-based QTL analysis with simple sequence repeat DNA markers (Supplementary Table S7) (Zhang et al. 2010), and the underlying gene could not be identified here until a large diverse germplasm panel was recently sequenced (Wang et al. 2016). In contrast to the previous LP tolerance QTL *qPE8* (*GmACP1*) (Zhang et al. 2014), *qPE19* was demonstrated to be pleiotropically responsible for all 7 LP-related traits and agronomic traits including seed yield, branch number, pod number, and seed weight. These yield-associated traits were all improved relative to the wild type after overexpression (Fig. 6), demonstrating that these traits can be simultaneously improved by *GmGDPD2* overexpression. This is also helpful to reduce P fertilizer application by enhancing Pi uptake, particularly in most of the subtropical or tropical regions in Asia that are deficient in Pi, where soybeans are one of the major crops cultivated as a primary source of plant-based protein (Lopez-Arredondo et al. 2014). In contrast, we did observe trade-off effects on plant height but not on the pod number and seed yield, which might be ideal for developing lodging-resistant cultivars to ensure production. The causal variants identified here may enable breeders to efficiently use *GmGDPD2* or pyramid it with other LP tolerance genes, such as *GmACP1* (Zhang et al. 2014) and *GmEIL4* (Yang et al. 2023), in breeding programs.

### The GmMyb73–GmGDPD2–GmGA2ox1 regulatory module confers LP tolerance

Plants evolve many different strategies with multiple layers of defense to cope with Pi deficiency stress, such as increasing Pi transport, regulating root architecture, and secreting phosphatases (Lopez-Arredondo et al. 2014; Song and Liu 2015). Changes in root system architecture, particularly the promotion of lateral root growth, appeared to be a conserved, typical response to LP stress in the plant kingdom because longer root hairs can enhance the total surface area of the roots to achieve more effective Pi acquisition in soil (Bates and Lynch 2000; Gahoonia and Nielsen 2004; Zhang et al. 2014). GmMyb73, GmGDPD2, and GmGA2ox1 can constitutively enhance or decrease root development or growth, as indicated in transgenic assays under LP conditions, and therefore might be major factors for increasing LP tolerance. Importantly, we confirmed that the 3 proteins function in a regulatory module, GmMyb73–GmGDPD2–GmGA2ox1, which greatly enhances our understanding and makes it possible to dissect the complex trait comprehensively.

In *Arabidopsis*, Cheng et al. (2011) characterized *GDPD* family genes and revealed that the expression of most members could be induced by Pi starvation and that mutation in *AtGDPD1* decreased Pi content in Pi-starved conditions. Similarly, the *GDPD* family in rice also responded to LP, and overexpression of *OsGDPD2* enhanced Pi content and biomass (Mehra and Giri 2016; Mehra et al. 2019). How the gene family is involved in the Pi starvation response is still unclear. Here, *GmGDPD2* is reported to exhibit an LP response in soybean; it controls major QTLs for multiple low Pi-associated traits, as revealed by both GWAS and linkage mapping, demonstrating its importance in LP responsiveness in soybean. The increased root production and yield-related traits in OE-*GmGDPD2* compared with the control implied that biomass enhancement might be a conserved function between GmGDPD2 and OsGDPD2 (Mehra et al. 2019). In *Arabidopsis*, *GA2ox1* is specifically expressed in the hypocotyl and lateral root primordium, and its overexpression reduces the levels of active GAs (Rieu et al. 2008; Li et al. 2019), which is required to increase root elongation by promoting cell division activity in the root meristem (Tanimoto 2012). Auxin is proposed to be a master regulator in altering root system architecture (Saini et al. 2013), and LP induces remobilization of auxin from root tips to root hair cells, leading to the promotion of root hair cell elongation (Parry 2018). Changes in auxin gradients then impact the formation of lateral roots and root hairs, thereby directly or indirectly influencing Pi uptake and/or Pi translocation (Yi et al. 2021). Similarly, we observed denser cells in the root apical meristem and elongation zones in OE-*GmGDPD2* root tips (Fig. 2L). The resulting increase in the production of lateral roots greatly contributed to the improved PC and PAE in shoots, which led to enhancement in plant growth and development in OE-*GmGDPD2* plants (Veneklaas et al. 2012). These results collectively suggest that GmGDPD2 modulates root growth and development likely through its regulation of GmGA2ox1-associated GA and auxin biosynthesis because the levels of selected GAs and auxin were significantly increased in KO-*GmGDPD2*/*GmGA2ox1* plants (Fig. 5, J to M, Supplementary Fig. S23). This preliminary conclusion is supported by the notion that GA levels control the root meristematic cell division rate, which modulates the rate of root elongation (Achard et al. 2009; Barker et al. 2021). The GmGDPD2–GmGA2ox1 interaction on the plasmatic membrane, likely in roots, implies the location of LP-related GA biosynthesis or the receptors that perceive LP signals, such as P transporters that are located on the cell membrane likely function as Pi sensors in the epidermal cells of the root hair (Karthikeyan et al. 2002; Mudge et al. 2002). In addition, our preliminary investigation suggests that the LP tolerance mediated by GmGDPD2 might be independent of canonical ethylene-associated LP tolerance that has been reported in various plants including soybean (Song and Liu 2015; Liu et al. 2020). We cannot exclude the possibility that GmGDPD2 might also be involved in the regulation of other genes associated with root development, such as Gm*LPR2*, Gm*RGI1*, and *GmEXPANSIN-A7*, which were differentially expressed between transgenic roots and the wild-type roots, and were likely act downstream of or tightly associated with the GmGDPD2 regulatory pathway, but how GmGDPD2 influences them remains to be investigated.

Myb73 is an R2R3-type MYB transcription factor that is reportedly involved in salinity tolerance (Kim et al. 2013), nitrogen-associated metabolism (Gaudinier et al. 2018), and *UV RESISTANCE LOCUS 8* (*UVR8*)-associated lateral root growth in *Arabidopsis* (Yang et al. 2020a). Here, we expanded the knowledge that GmMyb73 could respond to LP stress and negatively regulate root growth and development. Negative regulation was also observed for an *Arabidopsis Myb73* knockout mutant, which exhibited better salt tolerance than the wild type (Kim et al. 2013), suggesting a conserved molecular mode of action in regulating low P and salt stress. The observations of the effects of *GmGDPD2* expression on roots, the *GmMyb73* expression pattern, and the GmMyb73–GmGDPD2 interaction suggest that GmMyb73 constrains the growth and development of lateral roots, likely through its negative regulation of the GmGDPD2–GmGA2ox1 module, which is associated with the GA response. Interestingly, we indeed predicted response elements associated with ABA responsiveness, auxin responsiveness, and defense and stress responsiveness in the promoter region of *GmMyb73*, such as ABRE (ACGTG), TGA element (AACGAC), and TC-rich repeats (ATTCTCTAAC) (Supplementary Fig. S26). This preliminary speculation is consistent with a report that GmMyb73 affects lateral root growth through the regulation of the auxin response in *Arabidopsis* (Yang et al. 2020a). There is a possibility that the synergistic interaction between auxin and GA promotes cell division in LP-stressed roots, which has mainly been reported in stems but rarely in roots (Tanimoto 2005).

**Figure 7.**
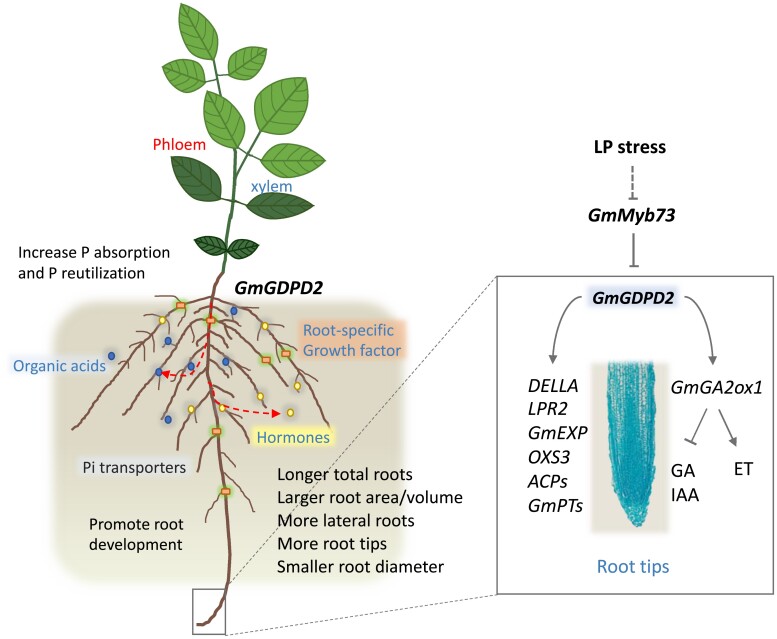
The Myb73–GDPD2–GA2ox1 module regulates low P tolerance in soybean. The quantitative trait locus (QTL) gene *GmGDPD2* is the center of the regulatory model. Low P (LP) stress downregulated the expression of *GmMyb73* which encodes a negative regulator of *GmGDPD2*. GmGDPD2 enhances LP stress tolerance by promoting root architecture traits such as RL, root areas, and RV, which allows the roots to acquire sufficient P in P-deficient soil. This regulatory process is likely achieved by its physical interaction with GmGA2ox1, leading to modification of the concentrations of phytohormones (e.g. GA, IAA) to promote cell division in the root tip zone, or through its influences on the expressions of many genes such as *GmPTs* that are important for LP stress tolerance. GA, gibberellin; IAA, 3-Indoleacetic acid; ET, ethylene; *GmGDPD2*, *GLYCEROPHOSPHORYL DIESTER PHOSPHODIESTERASE 2*; *GmGA2ox1*, *GA 2-DIOXYGENASE 1*, *DELLA*, growth repressor gene; LPR2, *LOW PHOSPHATE ROOT2*; *GmEXP*, *EXPANSIN*; *OXS3*, oxidative stress tolerance gene *OXIDATIVE STRESS 3*; *ACP*s, *ACID PHOSPHATASE*s; *GmPT*s, *PHOSPHATE TRANSPORTERs*.

In conclusion, we uncovered a major QTL gene, *GmGDPD2*. GmGDPD2 controls multiple LP-associated root morphological and PAE traits. Identification of its negative regulator GmMyb73 and its interacting protein GmGA2ox1 followed by consistent results allowed us to propose a regulatory module GmMyb73–GmGDPD2–GmGA2ox1. This major QTL gene-centered module is likely a major module regulating LP tolerance in soybean (Fig. 7). The negative regulation of Myb73–GDPD2 and positive regulation of GDPD2–GA2ox1 allow the expression plasticity of the model to achieve high efficiency in LP stress tolerance. Based on the mode of action in regulating LP tolerance, we could enhance soybean LP stress tolerance by adjusting the expression level of the module to remodel the root architecture traits and/or alter the expression levels of associated genes and/or hormone levels to enable maximal acquisition of available Pi in soils to support plant growth and development. These results also enhanced our understanding of the complex mechanism of LP tolerance in soybean, and further functional examination surrounding the module would advance our comprehension of LP responsiveness in plants.

## Materials and methods

### Plant materials and growth conditions

A total of 367 soybean (*G. max*) accessions (including 105 wild soybeans and 262 cultivated accessions) derived from 26 provinces within 6 ecological regions [North (I), Huang-Huai-Hai region (II), Yangtze River (III), Central and Southern regions (IV), Southwest plateau (V), and tropical region of South China (VI)] in China (latitude 53 to 24 °N and longitude 134 to 97 °E) (Wang and Gai 2002) were used for the evaluation of Pi efficiency-related traits and GWAS. A recombinant inbred line (RIL) population consisting of 127 F_11–12_ RILs was used for QTL linkage analysis. The population (DW population) was derived from a genetic cross between Dongnong50 (low Pi efficiency genotype) and Williams82 (W82, high Pi efficiency genotype) using the single-seed descent method (Kong et al. 2018).

Hydroponics experiments were conducted in 2018 and 2019 to assess the traits related to Pi efficiency as previously described (Li et al. 2016). Each hydroponics experiment was independently replicated at least 3 times. Briefly, seed germination and growth were performed in an artificial climate chamber (28/20°C day/night temperature, 10 h light/14 h dark photoperiod, 130–180 *μ*mol photons m^−2^ s^−1^ light intensity, and 50% to 60% relative humidity). The seeds were surface-sterilized with chlorine and germinated in sterile vermiculite. When the true leaves were fully expanded, healthy seedlings were transferred into modified one-half Hoagland's nutrient solution supplemented with 500 *µ*M Pi [normal Pi supply (NP)]. After 3 d of cultivation, half of the seedlings were transferred to modified one-half Hoagland's nutrient solution lacking Pi [5 *µ*M Pi, low Pi supply (LP)] as LP treatments, for 14 d, and the other half remained in +P conditions as NP controls. The plants were grown in plastic cones that were arranged in 60-well hydroponic tanks (70 × 50 × 30 cm) using a completely randomized block design (pH 5.8, the solution was renewed every 3 d). Three plants with similar growth of each line in each treatment were selected for LP-related trait evaluation. The hydroponics experiment for wild types and different overexpression and knockout lines were the same as described above but varied in the length of NP and LP treatment time. The details for the length of treatment time were shown in the figure legends.

T3 or T4 generation transgenic plants were grown in greenhouse trials for agronomic measurement in 2021 in Zhengzhou, China. A completely randomized design with a pot with a capacity of 10 kg of soil was used for these experiments, and 3 replicates were performed, with 3 plants per replicate. The soil had a moderate P concentration of 10 mg kg^−1^ available Pi (most of China's arable land is moderately P deficient soil, and 40% of the world's arable land is moderately Pi deficient soil) and contained 0.2 g kg^−1^ total nitrogen, 50 m kg^−1^ available K and 10 g kg^−1^ organic matter (Zhang et al. 2014). The double mutants for KO-*GmGDPD2*/*GmGA2ox1* were identified in the offspring of the cross between KO-*GmGDPD2* and KO-*GmGA2ox1*, and the homozygous lines were confirmed by Sanger sequencing.

### Phenotypic measurement

Seven traits reflecting morphological and physiological responses to Pi supply were measured through hydroponics experiments in 2018 and 2019. These traits included 5 root architecture-related traits, root length (RL), the number of root tips (RN), RA, root diameter (RD), and root volume (RV), and 2 P efficiency-related traits [Pi concentration (PC) and Pi absorption efficiency (PAE)] and were measured under LP (5 *μ*M, Pi) and NP (500 *μ*M, Pi) conditions. To well reflect the LP tolerance response, we used the ratio of measured values for each trait for 1 line under LP relative to NP as an LP-related trait, for example, RRL = RL under LP treatment/RL under NP treatment. All 7 traits were measured in the association panel, and 4 traits (RRA, RRL, RRN, and RRV) were measured for the RIL population.

The RL, RN, RA, RD, and RV traits were analyzed with an automatic root scanning apparatus (EPSON Expression 11000XL) equipped with WinRHIZO software. First, cleaned roots were placed in transparent root plates, 2-mm deep purified water was added to the plate, roots were spread evenly with tweezers to avoid overlapping and crossing as much as possible, and the plate was covered with cardboard. Second, roots were scanned with 400-dpi resolution ratio, and the root image was saved as a tiff file. Then, the root image was analyzed to measure root-related traits through WinRHIZO software.

PC trait was measured as previously described (Zhang et al. 2014). The plant enzymes were deactivated by heat-induced denaturation at 105°C for 60 min; the samples were then oven-dried at 65°C for 3 d. The dried samples were milled and subsequently digested with concentrated H_2_SO_4_ and H_2_O_2_ to facilitate the determination of P concentration using the molybdate-blue colorimetric method (Murphy and Riley 1962). PAE was calculated as the total Pi in the plant (mg plant^−1^) (Gourley et al. 1994).

In addition, other agronomic traits were also investigated at corresponding periods for the transgenic lines planted in the greenhouse, including yield, plant height, photosynthesis-related traits, branch number, pod number, and 100-seed weight at full maturation, and seed quality-related traits (oil content and protein content) using a near-infrared spectrophotometer (NIR) seed analyzer (DA7200, Perten Instruments, Huddinge, Sweden).

### Statistical analysis

The averages and standard deviations (SDs) of phenotypic data from different replicates were calculated for each environment to determine significant variations in P efficiency-related traits within the GWAS population and RIL population, as well as to assess the appropriateness of further genetic analysis. We performed analysis of variance (ANOVA) on phenotypic data using the mixed linear model procedure in SPSS Statistics 17.0 (SPSS, Inc., Chicago, IL, USA). Here, the genotype (G) and replications were treated as fixed factors, while the combinations of year and location were regarded as environments (E), and the genotype-by-environment (G × E) interaction was treated as a random factor. The data analysis was conducted using SPSS 17.0, and the mean values were used for Pearson correlation coefficients, descriptive statistics, frequency distribution, and Student's *t*-tests. The results were further visualized using GraphPad Prism version 8.0 software (GraphPad Software, San Diego, California). The presence of statistically significant differences is denoted by * (*P* < 0.05) and ** (*P* < 0.01).

### GWAS for Pi efficiency-related traits

The population of 367 soybean accessions was genotyped using the NJAU 355K SoySNP array as previously described, and the structure analysis showed that they were partitioned into 5 groups and were suitable for GWAS (Wang et al. 2016). In brief, all 367 accessions were genotyped with a DQC threshold of 0.82, and a call rate of 97% was included for further analysis. The final set of 213,317 high-quality SNPs (MAF > 0.05, missing rate <10%) distributed over the whole soybean genome were selected, whose physical position was based on Glyma.Wm82.a1.v1.1. Then, we renewed their physical position based on the Wm82.a4 reference genome (Supplementary Data Set 1). Association analyses were performed using a mixed-model approach with the rMVP R package for the 7 P efficiency-related traits (Yin et al. 2021). The significant association threshold was set as 1/*n* (*n*, total SNP number). Sequence variation in genes was retrieved from the resequencing data of 559 soybean accessions (Lu et al. 2020). Linkage disequilibrium (LD, as *r*^2^) of the soybean genome for 367 soybean accessions based on renewed position was estimated and shown through LDBlock Show-1.4 (https://github.com/hewm2008/LDBlockShow/archive/v1.40.tar.gz). The LD matrices of 84 SNP markers within the 500 kb interval surrounding the lead SNP (AX-94187201) were estimated and shown as heatmaps through LDBlock Show-1.4.

For the mapping of the candidate gene, we used 3,582,767 high-quality SNPs (MAF > 0.05) (Lu et al. 2020) to perform candidate gene-based association analyses for RRA in the GWAS population. The sequencing data have been deposited into the Genome Sequence Archive (GSA) database in the BIG Data Center (http://gsa.big.ac.cn/index.jsp) under accession number PRJCA001691 and into the NCBI database under accession number PRJNA608146. Protein structures for different haplotypes of candidate genes were predicted by I-TASSER (Yang et al. 2015), compared using RaptorX (TMscore 0.797) (Wang et al. 2013), and visualized with iCn3D (https://structure.ncbi.nlm.nih.gov/Structure/icn3d/).

### QTL linkage mapping

The linkage maps for the DW population contained 2,063-bin markers (Kong et al. 2018), the genetic length was 2,458.55 cM with an average marker interval distance of 1.20 cM, and the physical position of the SNPs was based on Wm82.a2.v1. The QTL analysis was subsequently performed using the inclusive composite interval mapping (ICIM) method to identify additive QTLs (Meng et al. 2015). The *P* values for entering variables (PIN) and removing variables (POUT) were set at 0.01 and 0.02, respectively, and the scanning step was set at 2 cM. The LOD thresholds for QTLs were determined by a 1,000-permutation test at a 95% confidence level. The proportion of observed phenotypic variance explained by each additive QTL and the corresponding additive effects were also estimated.

### DNA/RNA extraction and PCR analysis

Genomic DNA was extracted from soybean leaves by a modified CTAB method (Murray and Thompson 1980). PCR was performed in a 50-*μ*L reaction consisting of 1 *μ*L of DNA, 10 *μ*L of 2×PCR buffer, 10 *μ*L of 2 mm deoxyribose adenine triphosphates, 10 *μ*L of ddH_2_O, 1.5 *μ*L of each primer (10 mm), and 1 *μ*L of KOD FX polymerase. For expression analysis, roots and leaves were harvested separately at the following time points: 0, 3h, 12 h, 1 d, 3 d, 7 d, and 14 d after transferring to LP conditions, flash frozen in liquid nitrogen, and stored at −80°C. A total RNA Kit (AC0103, Sparkjade, Shandong, China) was used for RNA isolation. Approximately 1 *μ*g of total RNA was used for cDNA synthesis with a reverse transcription kit (AG0304, Sparkjade, Shandong, China). For RT-qPCR analysis, a 20-*μ*L volume was used with 2 *μ*L cDNA, 0.5 *μ*M specific primers, and 10 *μ*L 2× SYBR Green Mix (AH0101, Sparkjade), supplemented to 20 *μ*L with ddH_2_O, on a Bio-Rad real-time PCR machine (CFX96 Touch, Bio-Rad) according to the manufacturer's instructions. The soybean *Tubulin* (GenBank accession AY907703) was used as the internal control. All primers used for RT-qPCR analysis are listed in Supplementary Table S8. The analysis of relative gene expression data was performed using the 2^(−ΔΔC[T])^ method (Livak and Schmittgen 2001). Three biological and 3 technical replicates were used.

### Sequence alignment

The full-length amino acid sequences of GDPD2 in *Arabidopsis* and soybean were obtained from the National Center for Biotechnology Information (http://www.ncbi.nlm.nih.gov) database. DNAMAN was used for multiple sequence alignment according to the default parameters.

### Subcellular localization

Subcellular localization assays were performed mainly as previously described (Wang et al. 2023a). The full-length CDSs of *GmGDPD2*, *GmMyb73*, and *GmGA2ox1* were amplified from soybean cultivar Williams82 and subcloned and inserted into the modified pFGC5941 vector. The fusion constructs 35S:*GmGDPD2*-GFP, 35S:*GmMyb73*-GFP, and 35S:*GmGA2ox1*-GFP and control 35S:GFP were transformed into *Agrobacterium tumefaciens* strain EHA105 for the infiltration of *N. benthamiana* leaves. After 48 h, the transformed tissues were strained with DNA-specific nuclear strain 4′,6-diamidino-2-phenylindole (DAPI) for 10–15 min, and then fluorescent signals were monitored using a confocal laser scanning microscope (LSM710; Zeiss). Nuclei were indicated using IPM4-mCherry control and plasma membranes were labeled using PIP2A-mCherry. The excitation wavelengths for the GFP and mCherry signals were 488 and 587 nm, respectively. All fluorescence experiments were independently repeated at least 3 times.

### Vector construction and soybean transformation

The CDS of the candidate gene *GmGDPD2* from Williams82 (WT2, Hap5) was cloned and inserted into multiple cloning sites of pCAMBIA3300 with the selection marker gene *EPSPS*. The resulting recombinant 35S:*GmGDPD2* construct was transformed into the soybean cultivar Jack (WT1, Hap1) via the optimized *A. tumefaciens*-mediated soybean cotyledon node transformation system as previously described (Zhang et al. 2019a). For overexpression (OE) of *GmGA2ox1*, the CDS from Williams82 was cloned and inserted into pTF101 at the restriction sites *BamH*I and *Asc*I with a selection marker gene *Bar* (Chen et al. 2020). The resulting recombinant 35S:*GmA2ox1* construct was transformed into the soybean cultivar Jack (WT1, Hap1). The target sequence adapters for *GmGDPD2* or *GmGA2ox1* genes were designed using the web tool CRISPR-P (http://cbi.hzau.edu.cn/crispr/). The guide RNAs (sgRNA) were introduced into the linearized vector PGES401. The resulting vectors for *GmGDPD2* or *GmGA2ox1* genes were introduced into *A. tumefactions* strain EHA105 followed by transformation into soybean accessions Williams82 (WT2, Hap5) and Jack (WT1, Hap1), respectively, with a previously described approach (Tang et al. 2019).

For *Agrobacterium rhizogenes*-mediated transformation of soybean hairy roots, the coding sequence of *GmMyb73* was inserted into multiple cloning sites of the pTF101 vector (an overexpression binary vector) using the Hieff Clone Plus One Step Cloning. For the RNAi vector construct, a 446-bp specific segment of the *GmMyb73* exon fused with the attR1 and attR2 sequences was used and introduced into pB7GWIWG2 (II) using Gateway technology. The recombinant vector and the empty vectors were transformed into *A. rhizogenes* (K599) by the freeze-thaw method for soybean hairy root transformation in WT1 (Jack) as described previously with improvement (Fang et al. 2021). All primers used in this study are listed in Supplementary Table S8.

### RNA-seq analysis

WT1, WT2, 3 OE-*GmGDPD2* lines, and 3 KO-*GmGDPD2* lines were grown in NP and LP conditions with hydroponics experiments for 14 d, and root samples were collected for RNA extraction, library construction, and transcriptome sequencing. Approximately 2-g root tissue from each of the 3 OE-*GmGDPD2* lines or 3 KO-*GmGDPD2* lines was collected and pooled as 1 biological replicate, and 3 biological replicates were used per sample. Library construction was performed (Zhang et al. 2017) followed by sequencing on the Illumina HiSeq 2500 analyzer at Biomarker Technologies (Beijing, China), producing 200-bp paired-end reads. An average of 6.47-Gb clean data was generated. The quality-controlled reads were aligned to the Wm82.a4 reference genome with HISAT2 (https://daehwankimlab.github.io/hisat2/). Differential gene expression was determined using the DESeq R package (Anders and Huber 2010). Genes with an adjusted *P* < 0.05 and a fold change (FC) > 2.0 were defined as DEGs. Enrichment analysis of Gene Ontology of biological pathways (GOBPs) was performed using the GOseq R packages (Young et al. 2010). GOBPs with *P* < 0.01 were identified as enriched biological processes.

### Hormone determination

WT2 and 2 KO-*GmGDPD2/GmGA2ox1* lines were grown in NP and LP conditions with hydroponics experiments for 14 d. Each line was sown with 3 replicates, and mixed fresh root tissues of 3 plants for each repeat with consistent growth were collected for hormone measurement. Plant materials (50 mg fresh weight) were ground into powder with liquid nitrogen and extracted with 500 *μ*L H_2_O/ACN. Internal standards were added to plant samples before extraction. The supernatants were collected after centrifugation. The residue was re-extracted by repeating the steps above. Then, 10 *µ*L of triethylamine (TEA) and 10 *µ*L of 3-bromopropyltrimethylammonium bromide (BPTAB) were added to the resulting solution. The reaction solution was vortexed, incubated at 90°C for 1 h, and evaporated until dry under a nitrogen gas stream, redissolved in 100 *µ*L H_2_O/ACN, and filtered through a 0.22-*μ*m filter for further LC-MS analysis. The sample extracts were analyzed using a UPLC-ESI-MS/MS system (UPLC, ExionLC AD, ESI, Applied Biosystems 6500+ QTRAP LC-MS/MS System, MS, Applied Biosystems 6500 Triple Quadrupole). A specific set of MRM transitions was monitored for each period according to the plant hormones eluted within this period. GA contents were detected by MetWare (http://www.metware.cn/) based on the AB Sciex QTRAP 6500 LC-MS/MS platform.

### ELISA analysis of GA2 dioxygenase activity

For the GA2 dioxygenase activity assay in response to NP and LP, 10-day-old soybean seedlings of WT2 and 2 KO-*GmGDPD2/GmGA2ox1* lines were grown in NP and LP conditions with hydroponics experiments for 7 d, mixed fresh root tissues of 3 plants for each line with consistent growth were collected for GA2 dioxygenase activity determination, and each mixed sample was tested 3 times. An enzyme-linked immunosorbent assay (ELISA) kit (YX-E28688G) was used for GA2 dioxygenase activity according to the manufacturer's instructions (Sinobest Bio, China). Briefly, soybean root samples of approximately 0.1 g were weighed, 1 mL of extract was added, and the samples were ground into a homogenate on ice. Samples were then centrifuged at 10,000 × *g* at 4°C for 10 min, and the supernatant was placed on ice for laccase activity testing.

### Hormone supplementation experiment

To evaluate the relationship between GmGDPD2 and GA, we selected WT2 and 3 KO-*GmGDPD2* lines for the hormone supplementation experiment. Twenty-four seeds were selected for each line and germinated in sterile vermiculite. When the true leaves were fully expanded, 12 and 12 healthy seedlings were transferred into modified one-half Hoagland's nutrient solution supplemented with 500 *µ*M Pi [normal Pi supply (NP)] and 5 *µ*M Pi [normal Pi supply (LP)], respectively. For the KO-*GmGDPD2* lines, 6 of the 12 seedlings under NP conditions and 6 of the 12 seedings under LP conditions were treated with a GA inhibitor (Paclobutrazol) on the 3rd and 6th day. One plant for each treatment was selected for the evaluation of root fresh weight, root dry weight, RL, root area, RN, RV and plant height on the 8th day.

### Determination of organic acids secreted by roots

WT1, WT2, 3 OE-*GmGDPD2* lines, and 3 KO-*GmGDPD2* lines were first germinated in sterile vermiculite. When the true leaves were fully expanded, 6 and 6 healthy seedlings were transferred into modified one-half Hoagland's nutrient solution supplemented with 500 *µ*M Pi [normal Pi supply (NP)] and 5 *µ*M Pi [normal Pi supply (LP)], respectively. After NP and LP treatment for 8 d, the roots of all plants were cleaned with distilled water, 6 plants for each treatment were divided into 3 replicates, and each replicate (2 plants) was transferred to a black plastic bottle containing 50 mL of deionized water. Six hours later, the solution containing root exudates was collected and stored in a −20°C refrigerator. Ten milliliters of the solution were first filtered through a 0.45 *μ*m PES Syring Filter (Biosharp), dried through a vacuum freeze-drying instrument (LGJ-50FD), and dissolved to 1 mL. Finally, the concentrations of malic acid and oxalic acid were tested through a Thermos Scientific DIONEX ICS-5000+.

### Pi depletion assay

The seeds of WT1, WT2, OE-*GmGDPD2* lines, KO-*GmGDPD2* lines, OE-*GmGA2ox1* lines, KO-*GmGA2ox1* lines, and KO-*GmGDPD2/GmGA2ox1* lines were first germinated in sterile vermiculite. When the true leaves were fully expanded, 6 and 6 healthy seedlings were transferred into modified one-half Hoagland's nutrient solution supplemented with 500 *µ*M Pi [normal Pi supply (NP)] and 5 *µ*M Pi [normal Pi supply (LP)], respectively. After NP and LP treatment for 8 d, the roots of all plants were cleaned with distilled water, and all plants were transferred to deionized water for 22 h to deplete the Pi in the plants. Then, 6 plants for each treatment were divided into 3 replicates, and each replicate (2 plants) was transferred to a black plastic bottle containing 500 mL depletion solution. The component of the depletion solution was the same as the modified one-half Hoagland's nutrient solution supplemented with 500 *µ*M Pi [normal Pi supply (NP)]. A sample (5 mL) of the depletion solution was collected at 0, 3, 6, 9, 12, and 24 h. The 5 mL test solution was placed into a 50 mL volumetric flask and diluted to 30 mL with distilled water. Then, 2 drops of dinitrophenol indicator were added to the volumetric flask, 4 mol/L NaOH solution was added dropwise until the solution turned yellow, 2 mol/L sulfuric acid solution was added dropwise to just fade the yellow color of the solution (pH = 3). Five milliliters of molybdenum antimony reagent was added and then the solution was diluted to 50 mL with distilled water. After 30 min of storage, the color was compared at a wavelength of 880 nm. Before measuring the samples, the absorbance of a blank test was adjusted to 0.

### Protein expression

To obtain a specific polyclonal antibody against endogenous soybean GDPD, a peptide (LYDHYMTKGED) screened for potential antigenic epitopes in the GDPD protein N-terminal was synthesized and conjugated either to keyhole limpet hemocyanin (KLH) for immunization or to bovine serum albumin (BSA) for ELISA with cysteine in the N terminus (Royobiotech, Shanghai, China). Six-week-old SPF Balb/c mice (Zhengzhou Normal University, Zhengzhou, China) were immunized with peptide-KLH together with an adjuvant (KX0210041; Biodragon, Suzhou, China). When serum titers exceeded 1:100,000, serum was collected, and IgG was purified with the Protein A resin kit (L00210-100; GenScript, Nanjing, China) according to the manufacturer’s instructions.

Roots of WT1, WT2, OE-*GmGDPD2*, and KO-*GmGDPD2* lines were treated under NP and LP conditions for 7 d, then their roots were collected and ground using liquid nitrogen. Protein lysis solution (KTP3008, Abbkine, Beijing, China) was added for lysis, and then the suspension was placed on ice for 20 m before centrifuging at 12,000 rpm for 10 m. The obtained supernatant was then mixed with 5 × SDS-PAGE protein loading buffer (20315ES20, Yesen, Shanghai, China), and boiled at 100°C for 5 min. After cooling to room temperature, SDS-PAGE analysis was performed and then transferred to a nitrocellulose membrane for detection using Anti GDPD2 (1:5,000) and Anti Flag (1:3,000) (B1084, Biodragon, Suzhou, China).

### In vitro autophosphorylation assays

An in vitro kinase activity assay was performed mainly as previously described (Du et al. 2022) with minor modifications. The total volume of 50 *µ*L phosphorylation buffer contained 25 mm Tris-HCl (pH 7.5), 2.5 mm MgCl_2_, 2.5 mm MnCl_2_, 1 mm CaCl_2_, 10% (vol/vol) glycerol, 1 mm DTT, and 1 *μ*g His-GmGDPD2-KD. Phosphorylation was initiated by the addition of 1 mm ATP. In addition, 10 mm EGTA was added to determine the function of Ca^2+^ in this process (Zhao et al. 2021). Reactions were maintained at 30°C for 20 min, terminated by the addition of 4 × SDS protein loading buffer, and subsequently boiled for 5 min. The samples were then separated by SDS-PAGE, transferred onto a 0.45 *µ*m nitrocellulose membrane, and analyzed using anti-His antibody (1:5,000). Phosphorylation levels were determined by Phos-tag Western blot (Phos-tag, APExBIO, F4002) and normal Western blot (normal WB).

### GUS histochemical analysis of transgenic soybean

The *GmGDPD2* promoter was subcloned into the pCAMBIA1381Z vector harboring the GUS gene and transformed into *A. rhizogenes* K599 to induce soybean hairy roots. The induced soybean hairy roots were grown in NP and LP conditions for 7 d, and hairy roots from different treatments were collected. GUS activity was detected in situ by incubating soybean hairy roots in 5-bromo-4-chloro-3-indolyl glucuronide (X-gluc) solution overnight at 37°C. After staining, the tissues were washed with ethanol (70% v/v) and then photographed.

### Histological staining

Paraffin section. The 2 wild types (WT1 and WT2), OE-*GmGDPD2*, and KO-*GmGDPD2* lines were treated under NP and LP conditions for 7 d, and roots from different treatments were collected. All collected samples were vacuum infiltrated for 15 min and lightly fixed in FAA at 4°C for 20–30 min. Then, the samples were embedded in wax and sectioned. Finally, the tissue sections were stained with 1% toluidine blue solution and dehydrated with an ethanol gradient. The sections were imaged using an Olympus SZX16 stereomicroscope (Olympus SZX16, Japan).

### Yeast-hybrid assays

Y2H assays were performed using the DUAL membrane system (Dualsystems Biotech AG, Schlieren, and Switzerland) and conducted as described previously (Cui et al. 2022). The full-length CDS of *GmGDPD2* was introduced into the pBT3-SUC vector between the *Pst*I and *Nco*I sites as bait, and the bait plasmid was named pBT3-SUC-GmGDPD2. The full-length CDS of *GmGA2ox1* was inserted into the pPR3-N vector to construct the pPR3-N-GmGA2ox1 plasmid vector. The pBT3-SUC-GmGDPD2 and pOst1-NubI, pBT3-SUC-GmGDPD2 and pPR3-N, pSTU2-APP, and pNubG-Fe65 (as positive controls), and pSTU2-APP and pPR3-N (as negative controls) plasmids were transformed into NMY51. All yeast transformants were grown on SD/-Trp/-Leu or SD/-Trp/-Leu/-His/-Ade media for screening or interaction tests.

For the Y1H assay, the promoter of *GmGDPD2* was cloned and inserted into the BstBI-linearized pAbAi vector to generate the recombinant plasmid pAbAi-GmGDPD2, and the coding sequence of *GmMyb73* was cloned and inserted into the pGADT7 vector to generate pGADT7-GmMyb73. Then, both of them were transferred into the Y1H gold strain and tested on SD/-Leu medium with different concentrations of AbA (Aureobasidin A). The interactions of GmMyb73 with the promoter fragment of GmGDPD2 were tested on SD/-Leu medium with the tested ABA concentration. The primers used are listed in Supplementary Table S8.

### BiFC assay

*A. tumefaciens*-mediated transient transformation of *N. benthamiana* leaves was used for BiFC analysis. The CDSs of *GmGDPD2* and *GmGA2ox1* were PCR-amplified and cloned and inserted into the vectors 35S-SPYCE(M) and 35S-SPYNE173, respectively. We selected 2 highly homologous proteins, GmGDPD (Glyma.02G100400.1) and GmGA2ox8 (Glyma.15G093900.1), that showed 75% and 62% amino acid sequence identities with GmGDPD2 and GmGA2ox1, respectively, as the negative controls. GmGDPD and GmGA2ox8 were predicted to have the same location as GmGDPD2 and GmGA2ox1 through DeepLoc-2.0 (https://services.healthtech.dtu.dk/services/DeepLoc-2.0/), respectively. The CDSs of *GmGDPD* and *GmGA2ox8* were PCR-amplified and cloned and inserted into the vectors 35S-SPYCE(M) and 35S-SPYNE173, respectively. The fusion vectors were defined as GmGDPD2-YFP^C^, GmGDPD-YFP^C^, GmGA2ox1-YFP^N^, and GmGA2ox8-YFP^N^. Next, the following vector combinations (GmGDPD2-YFP^C^ vs. GmGA2ox1-YFP^N^; GmGDPD2-YFP^C^ vs. GmGA2ox8-YFP^N^; GmGDPD-YFP^C^ vs. GmGA2ox1-YFP^N^; YFP^C^ vs. YFP^N^) were cotransformed into 4-week-old *N. benthamiana* leaves. After 3 d, transformed *N. benthamiana* leaf epidermal cells were imaged on a confocal laser scanning microscope (Zeiss LSM780, Germany). The fluorescence signal of YFP staining was imaged at an excitation wavelength of 488 nm.

### Split-luciferase complementation assay

Luciferase complementation assays were performed as described previously (Chen et al. 2008). For vector construction, full-length CDSs were amplified and inserted into pCAMBIA1300-35S-LUC^N^ and pCAMBIA1300-35S-LUC^C^ to generate recombination plasmids. Two plasmid vectors for testing protein-protein interactions (GmGDPD2-LUC^N^ and GmGA2ox1-LUC^C^), together with the p19 silencing plasmid, were cotransfected into *N. benthamiana* leaves via *Agrobacterium* infiltration. In brief, *Agrobacterium* with LUC^N^ and LUC^C^ vectors was suspended and mixed in infiltration buffer (10 mm 2-(N-morpholino) ethanesulfonic acid [pH 5.6], 10 mm MgCl_2_, and 150 *μ*M acetosyringone). After 3 h of incubation at room temperature, the suspensions were infiltrated into young, fully expanded *N. benthamiana* leaves. Plants were then incubated at 22°C for 3 d before LUC activity was measured. Images were captured using the NightOWL II LB 983 low-light cooled charge-coupled device imaging apparatus.

### Promoter activity analysis using dual luciferase assay

The promoter of *GmGDPD2* and the ORF of *GmMyb73* were cloned and inserted into the pGreenII 0800-LUC vector using a Hieff Clone Plus One Step Cloning Kit (Yesen, 10911ES20) and pCAMBIA1305 vector respectively. Pro::LUC-35S::REN and 35S::GFP reporter constructs were transformed into *A. tumefaciens strain* GV3101, and the 2 vectors were used to infect *N. benthamiana* leaves together. LUC signals were collected by a Promega GloMax 20/20 chemiluminescence detector (Promega). LUC/REN was measured using a Dual-Luciferase Reporter Gene Assay Kit (Promega, Madison, WI, USA).

### Pull-down assays

To investigate the interaction between GmGDPD2 and GmGA2ox1, the CDS of *GmGDPD2* was amplified and cloned and inserted into pET28a, and *GmGA2ox1* was cloned and inserted into pGEX-4T-1. The recombinant fusion proteins were expressed in *E. coli* and purified with the corresponding affinity beads. Pull-down assays were performed as described previously with modifications (Li et al. 2021). In the assay, the purified recombinant His-GmGDPD2 was immobilized with Ni-Sepharose beads (Ni-Sepharose 6 Fast Flow; GE Healthcare) for 2 h at 4°C. Subsequently, the recombinant GST-GmGA2ox1 was incubated with prewashed beads for more than 8 h at 4°C before being washed with ice-cold phosphate-buffered saline (PBS). After boiling in 4 × SDS loading buffer, the pulled-down proteins were separated on a 10% SDS-polyacrylamide gel and analyzed using immunoblotting with anti-GST/His antibody (1:5000, M20001; Abmart, Shanghai, China).

### Statistical analysis

Statistical analyses were performed as described in each figure legend. Statistical data are provided in Supplementary Data Set 2.

### Accession numbers

Transcriptome raw data from this article can be found in the NCBI database (https://www.ncbi.nlm.nih.gov/bioproject/PRJNA1070210) under Bioproject ID PRJNA1070210. For other experimental data underlying this article will be shared on reasonable request to the corresponding author.

## Supplementary Material

koae041_Supplementary_Data

## Supplementary data

The following materials are available in the online version of this article.

**Supplementary Fig. S1.** Root phenotypic variation for DW RIL population and 367 diverse soybean accessions.

**Supplementary Fig. S2.** GWAS of phosphorus related traits and LD decay in 367 diverse soybean accessions.

**Supplementary Fig. S3.** Genes located in 15.74–16.24 mb region of chromosome 19 of the soybean genome.

**Supplementary Fig. S4.** Expression patterns of Glyma. 19G069500 and Glyma.19G0694002.

**Supplementary Fig. S5.** GUS staining of Glyma.19G069400 in developing roots of W82 under NP and LP treatment for 7 d.

**Supplementary Fig. S6.** Domain organization of GmGDPD2.

**Supplementary Fig. S7.** Detection analysis of His-GmGDPD2-KD recombinant protein.

**Supplementary Fig. S8.** *GmGDPD2* gene sequence of 9 soybean accessions.

**Supplementary Fig. S9.** Amino acid sequence of GmGDPD2 of 9 soybean accessions.

**Supplementary Fig. S10.** Superimposed predicted structures of Hap5 (pink) and Hap1 (blue) for GmGDPD2.

**Supplementary Fig. S11.** Identification of positive *GmGDPD2* transgenic lines.

**Supplementary Fig. S12.** Root traits between the *GmGDPD2* overexpression and knockout plants with the wild types under NP or LP conditions for 7 d.

**Supplementary Fig. S13.** Shoot traits between the *GmGDPD2* overexpression and knockout plants with the wild types under NP or LP conditions in the plot.

**Supplementary Fig. S14.** Oxalic acid and malic acid secreted from the root of *GmGDPD2* overexpression and knockout plants, and the wild types after NP and LP treatment for 8 d.

**Supplementary Fig. S15.** Gene expression comparison between *GmGDPD2* overexpression or knockout roots with corresponding wild types under NP or LP conditions for 14 d.

**Supplementary Fig. S16.** Gene expression patterns among *GmGDPD2* overexpression or knockout roots with corresponding wild types under NP condition.

**Supplementary Fig. S17.** Expression levels of *GmGA2ox1*.

**Supplementary Fig. S18.** Root related traits of WT2, KO-*GmGDPD2* and KO-*GmGDPD2* with paclobutrazol treatment under NP and LP condition for 8 d.

**Supplementary Fig. S19.** Identification of positive *GmGA2ox1* transgenic lines.

**Supplementary Fig. S20.** Comparisons of root and Pi efficiency traits between transgenic plants and the wild type plants after NP or LP treatments for 10 d.

**Supplementary Fig. S21.** Morphologic illustration of WT1, WT2, knockout (KO)-*GmGDPD2* plants, KO-*GmGA2ox1* plants and KO-*GmGA2ox1*/*GmGA2ox1* (double knockout) plants after NP or LP treatments for 9 d.

**Supplementary Fig. S22.** Pi-uptake rate measurement.

**Supplementary Fig. S23.** Phytohormone changes in roots of KO-*GmGA2ox1*/*GmGA2ox1* (double knockout) plants after NP or LP conditions for 14 d.

**Supplementary Fig. S24.** GA2 dioxygenase activity in roots of KO-*GmGA2ox1*/*GmGA2ox1* (double knockout) plants after NP or LP treatments for 7 d.

**Supplementary Fig. S25.** Phenotypic comparison between the *GmGDPD2* and *GmGA2ox1* transgenic lines with the respective wild types after maturation in the greenhouse.

**Supplementary Fig. S26.** Predicted cis-elements in *GmMyb73* promoter.

**Supplementary Table S1.** Descriptive statistical results for traits related to phosphate (Pi) efficiency in 127 recombinant inbred lines (RILs) and their parents in experiments conducted in 2018 and 2019.

**Supplementary Table S2.** Information for the 7 QTL identified by OTL mapping based on DW RIL population.

**Supplementary Table S3.** Descriptive statistical results for traits related to phosphate (Pi) efficiency in 367 soybean accessions in experiments conducted in 2018 and 2019.

**Supplementary Table S4.** Pearson correlation among the LP-related root and Pi efficiency traits.

**Supplementary Table S5.** Information of nine QTL identified through GWAS.

**Supplementary Table S6.** Identification of GmGDPD2 Hap5 through functional KSAP marker in 79 soybean lines.

**Supplementary Table S7.** Previously identified QTL for LP-related traits on chromosome 19.

**Supplementary Table S8.** Primers used in this study.

**Supplementary Data Set 1.** Renewed position for the 213,317 SNP markers that were used for GWAS based on Wm82.a4 genome.

**Supplementary Data Set 2.** Statistical analyses.

## Dive Curated Terms

The following phenotypic, genotypic, and functional terms are of significance to the work described in this paper:

AUX1 Gramene: AT2G38120
AUX1 Araport: AT2G38120

LPR2 Gramene: AT1G71040
LPR2 Araport: AT1G71040

MYB62 Gramene: AT1G68320
MYB62 Araport: AT1G68320

RGF1 Gramene: AT5G60810
RGF1 Araport: AT5G60810

RGI1 Gramene: AT3G24240
RGI1 Araport: AT3G24240

GmGDPD Gramene: Glyma.02G100400.1
GmGDPD Araport: Glyma.02G100400.1
