# Supplementary material for: The Myb73–GDPD2–GA2ox1 transcriptional regulatory module confers phosphate deficiency tolerance in soybean
Source: Plant Cell. 2024 Feb 12;36(6):2176–200. doi: 10.1093/plcell/koae041 (PMC11132883; doi:10.1093/plcell/koae041)
Supplement: koae041_Supplementary_Data [file koae041_supplementary_data.zip › TPC2023RA00539DR2_TPC2023RA00539DR2_Supplemental_Data.pdf]

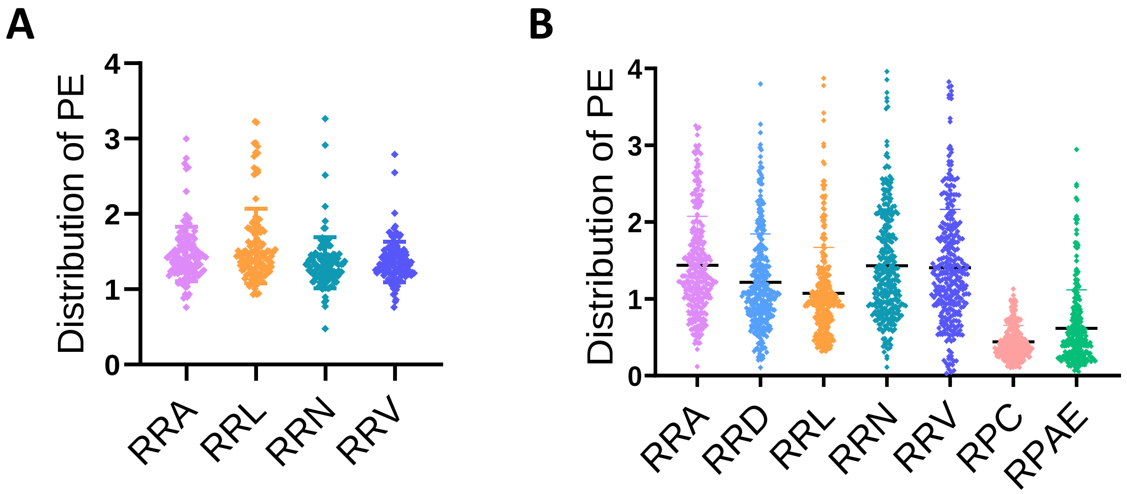

Supplemental Figure S1. **Root phenotypic variation for DW RIL population and 367 diverse soybean accessions. (Supports Fig. 1) A**, Root phenotype variation for DW RIL population under LP compared to NP condition. **B**, Root phenotype variation for 367 diverse soybean accessions under LP compared to NP conditions**.** PE, Pi efficiency related traits, including RRA, RRL, RRN, RRV, RRD, RPC, RPAE. The phenotypic of each line was the average value of phenotype evaluated in 2018 and 2019. RRA: relative root area; RRL: relative root length; RRN: relative number of hairy roots; RRV: relative root volume; RRD: Relative root diameter; RPC: relative P concentration; RPAE: relative P absorption efficiency (RPAE). LP, low P supply (5 μM, Pi); NP, normal P supply (500 μM, Pi).

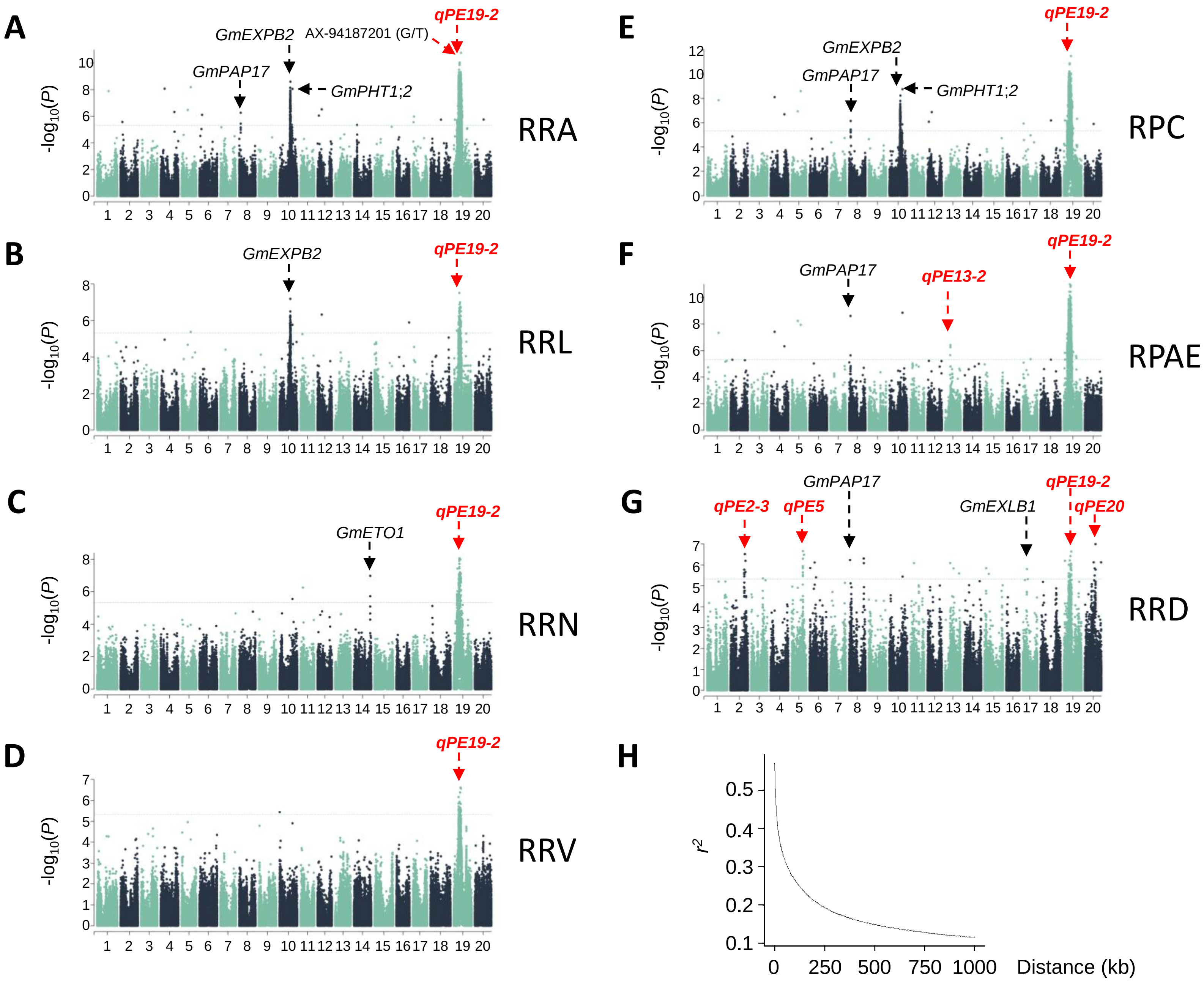

Supplemental Figure S2. **GWAS of phosphorus related traits and LD decay in 367 diverse soybean accessions. (Supports Fig. 1B) A-G**, GWAS of seven phosphorus related traits. **H**, LD decay of 367 diverse soybean accessions based on the new physical position (Table S9). RRA: relative root area; RRL: relative root length; RRN: relative number of hairy roots; RRV: relative root volume; RRD: Relative root diameter; RPC: relative Pi concentration; RPAE: relative Pi absorption efficiency (RPAE). *qPE19-2* was a new major QTL. PE, phosphate efficiency.

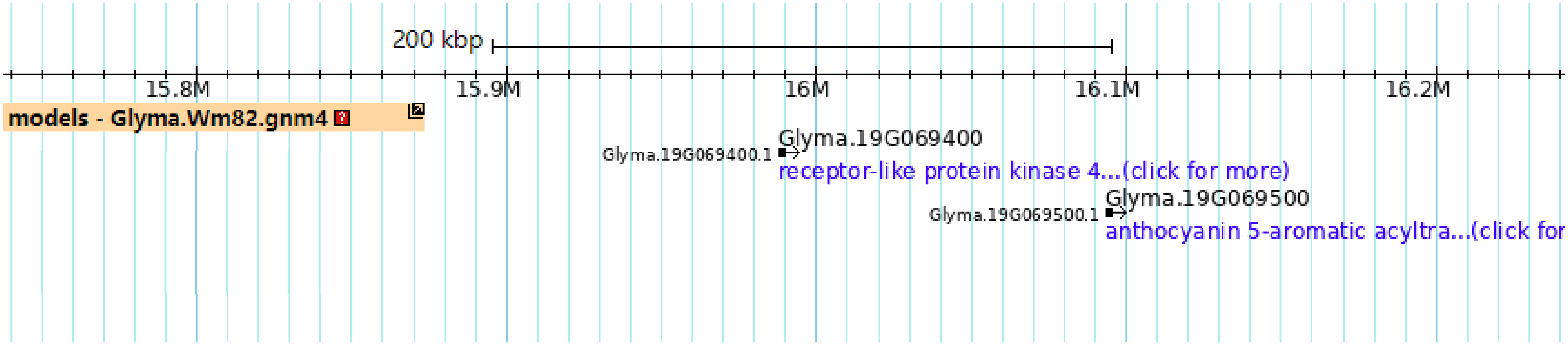

Supplemental Figure S3. **Genes located in 15.74 - 16.24 Mb region of chromosome 19 of the soybean genome. (Supports Fig. 1E)** The soybean genome was Wm82.a4 based on https://www.soybase.org/.

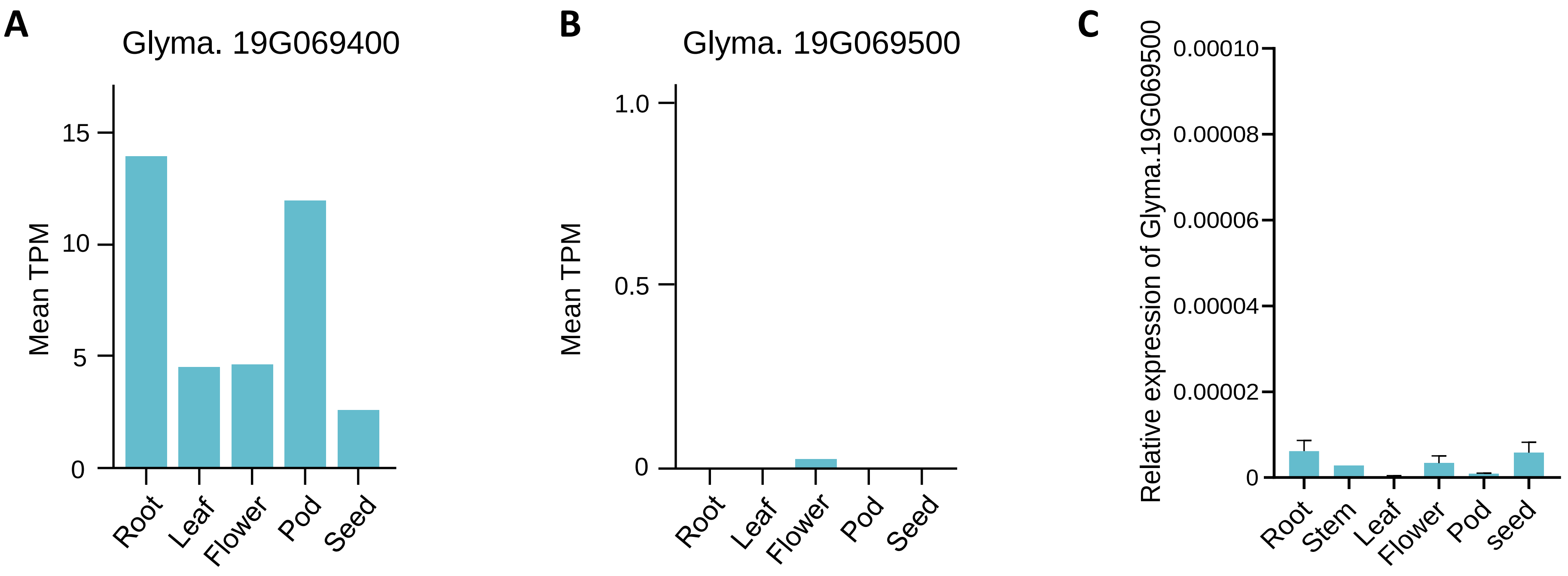

Supplemental Figure S4. **Expression patterns of Glyma.19G069500 and Glyma.19G069400. (Supports Fig. 1F) A**, **B** Expression pattern of Glyma.19G069400 and Glyma.19G069500 in different tissues. The expression data was download from https://soyatlas.venanciogroup.uenf.br/. **C**, Expression pattern of Glyma.19G069500 for Williams82 (W82) in six tissues under NP condition.

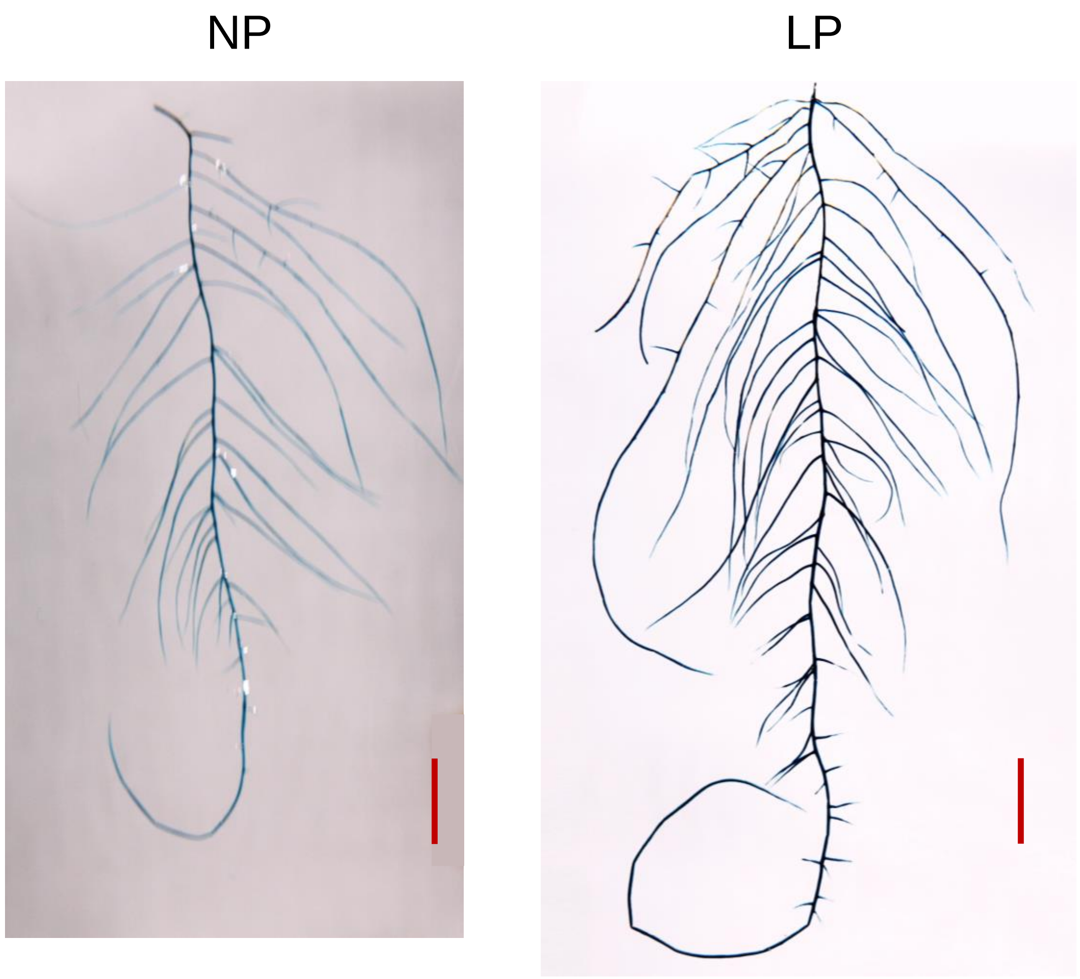

Supplemental Figure S5. **GUS staining of Glyma.19G069400 in developing roots of W82 under NP and LP treatment for 7 days**. **(Supports Fig. 1F)**
LP, low P supply (5 μM, Pi); NP, normal P supply (500 μM, Pi). Bar= 2 cm.

1	M L N F L N F S L L	L T L L F L L W K P	A I S K N G C T D K	C G R V R I Q F P F	Y L R N N S K L N H	T T T A Y S S G F D
61	L L C T D A D E T V	L E L P S V P I K L	F V K S I D Y K L Q	Q I Q I Y D P Q N C	L P R E L I K L G N	S S V A P F K F Q S
121	F G G N N V S F F R	C N S M S S S C P I	L Q L G Y D E G D F	I D P E I I S C T K	V S D V F S V Q W Q	V N N Y L G N A V V
181	M E W S K P D C S T	C E A Q G H K C K Y	K N G T Q G E T E C	F I C P T N R I S T	S S V V L I A A G G	I V G M I L L L V V
241	V K A L L H L Y D H	Y M T K G E D A R R	M E K F L E D Y R A	M K P T R F T Y A D	I K R I T N G F R E	S L G E G A H G A V
301	F K G M L S R E I L	V A V K I L N D T V	G D G K D F I N E V	G T M G K I H H V N	V V R L L G F C A D	G F H R A L V Y D F
361	F P N G S L Q R F L	A P P D N K D V F L	G W E K L Q Q I A L	G V A K G V E Y L H	L G C D Q R I I H F	D I N P H N I L I D
421	D H F V P L I T D F	G L A K L C P K N Q	S T V S I T A A R G	T L G Y I A P E V F	S R N F G N V S Y K	S D I Y S Y G M L L
481	L E M V G G R K N T	N M S A E E S F Q V	L Y P E W I H N L L	K S R D V Q V T I E	D E G D V R I A K K	L A I V G L W C I E
541	W N P I D R P S M K	T V I Q M L E G D G	D K L I A P P T P F	D K T S S S R T S V	V A P T R R Q N F E	L E I I N E I E E

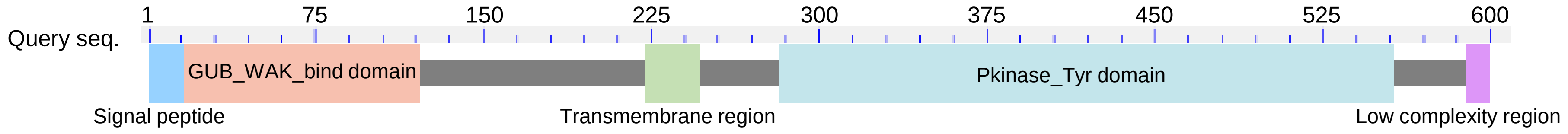

Supplemental Figure S6. Domain organization of GmGDPD2. (Supports Fig. 1) Domain analysis was based on SMART: Main page http://smart.embl-heidelberg.de/.

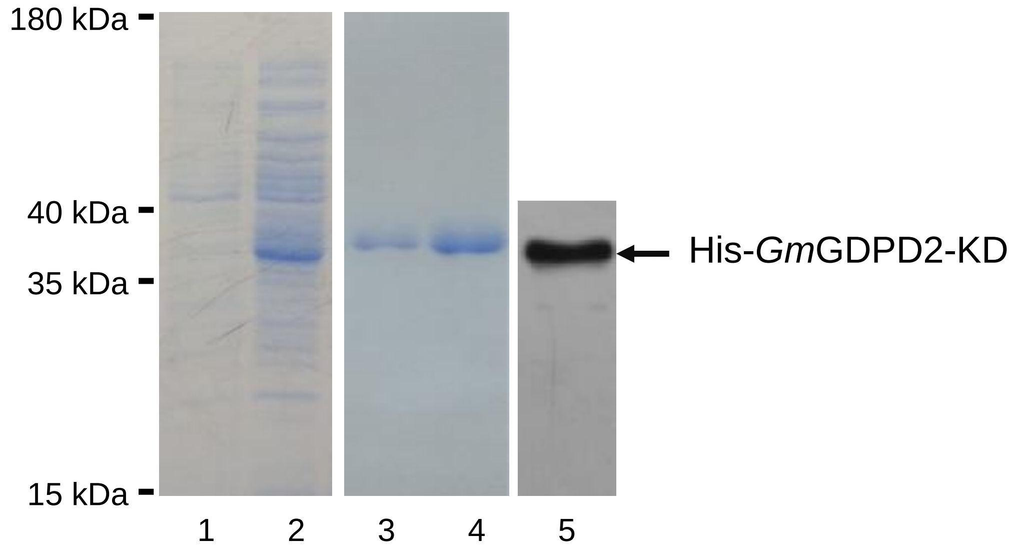

1:His
2: Expressed recombinant protein His-*Gm*GDPD2-KD
3,4: Purified recombinant protein His-*Gm*GDPD2-KD
5: WB analysis of His-*Gm*GDPD2-KD (Anti-His)

Supplemental Figure S7. **Detection analysis of His-GmGDPD2-KD recombinant protein. (Supports Fig. 1J)** Line 1, His. Line 2, expressed recombinant protein His-GmGDPD2-KD. Line 3 and line 4, purified recombinant protein His-*Gm*GDPD2-KD. Line 5, WB analysis of His-*Gm*GDPD2-KD (Anti-His).

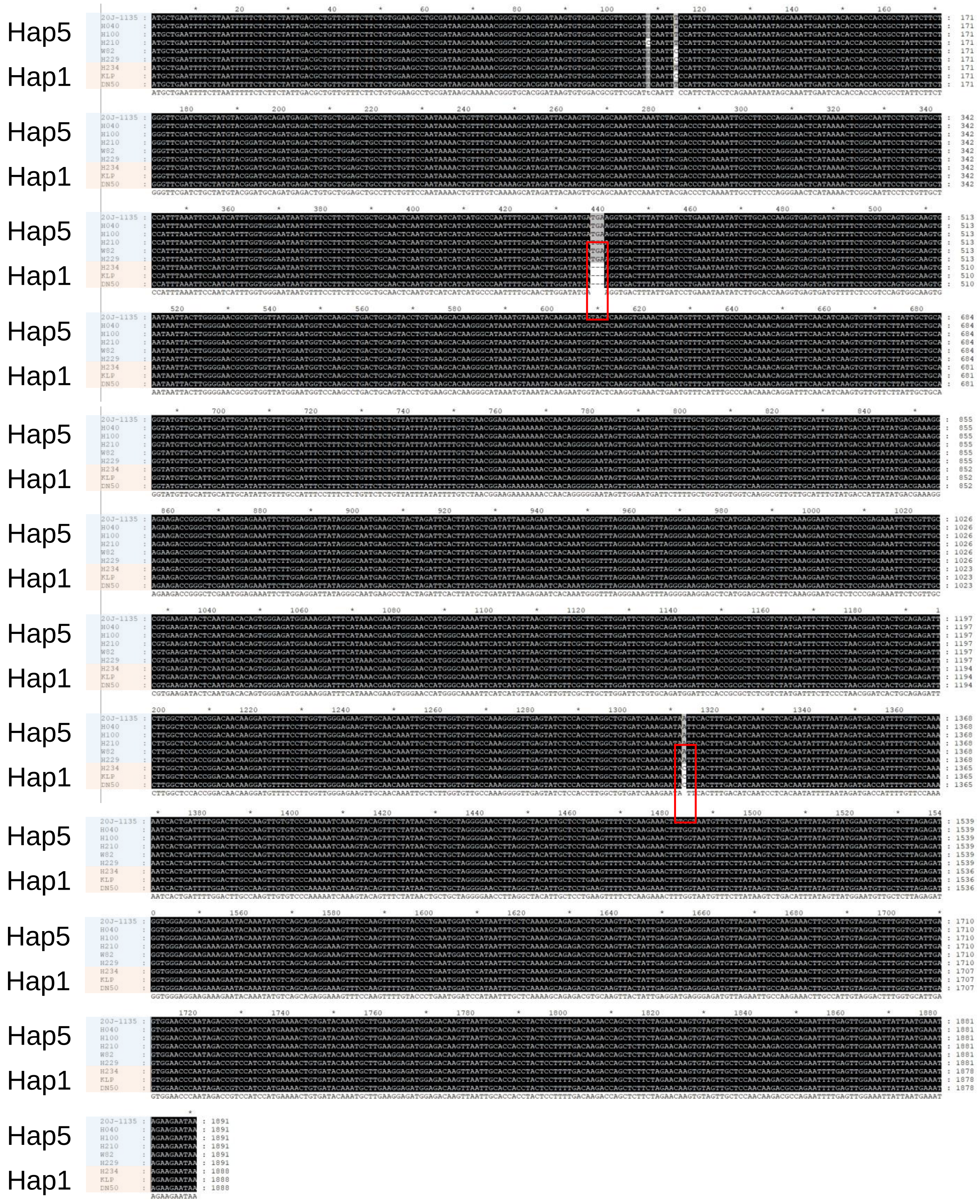

Supplemental Figure S8. ***GmGDPD2*** **gene sequence of 9 soybean accessions. (Supports Fig. 1H)** 20J-1135, H040, H100, H210, and H234 were Hap5, H234, KLP and DN50 were Hap1.

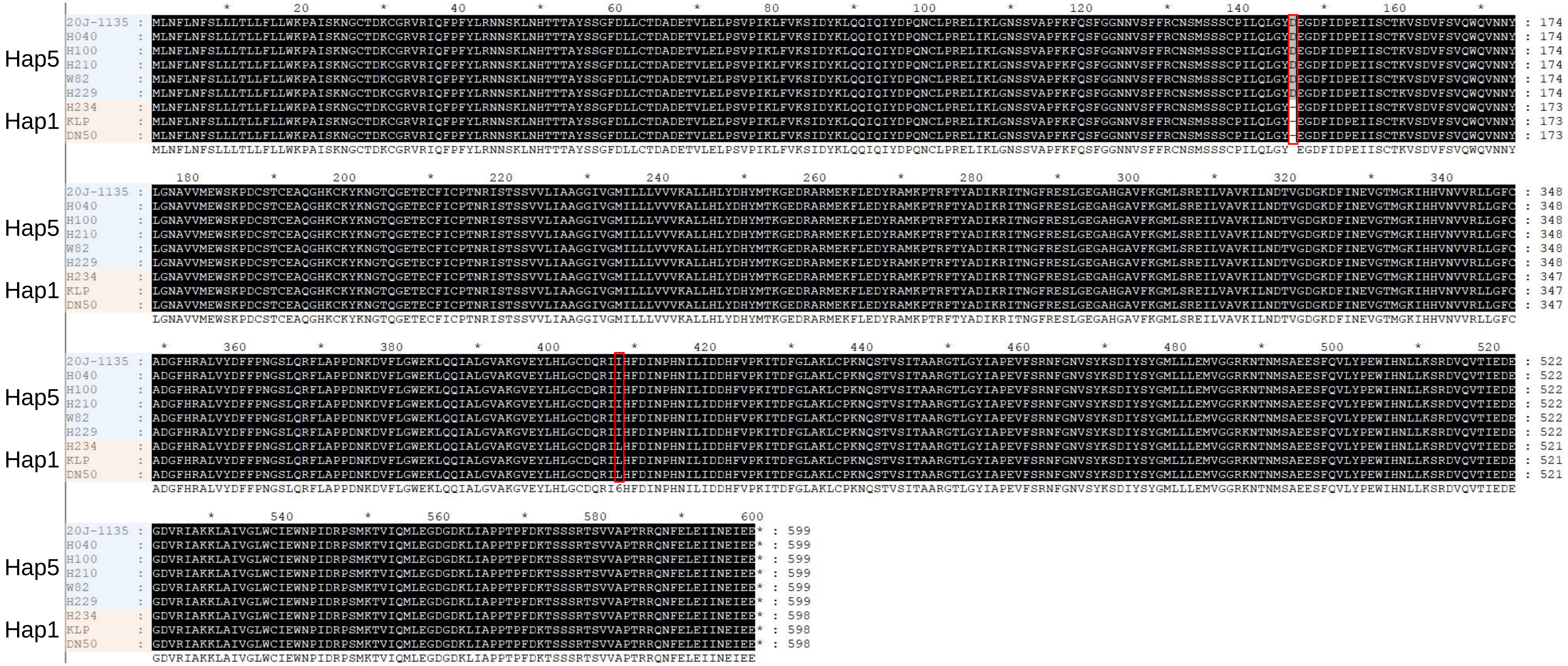

Supplemental Figure S9. Amino acid sequence of GmGDPD2 of 9 soybean accessions. (Supports Fig. 1H) 20J-1135, H040, H100, H210, and H234 were Hap5, H234, KLP and DN50 were Hap1.

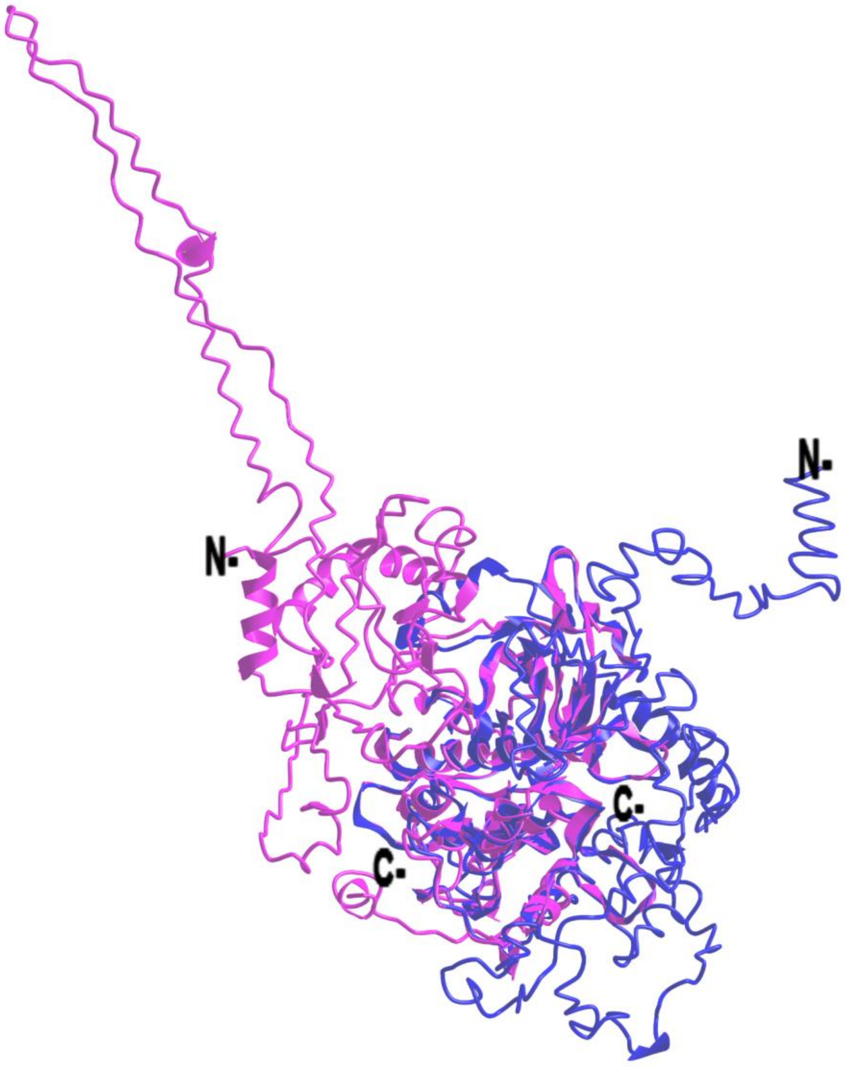

Supplemental Figure S10. Superimposed predicted structures of Hap5 (pink) and Hap1 (blue) for GmGDPD2. (Supports Fig. 1H) Two variations in CDS region were detected based on the sequences of three Hap1 and six Hap5 accessions (Fig. S8), and the TGA deletion in Hap1 led to a deletion of Asp (D), and the SNP variation in Hap1 changed the Ile (I) to Leu (L) (Fig. S9)**.** Structure prediction of Hap1 and Hap 5 indicated that the two variations may greatly change the protein structure of GmGDPD2 with almost distinct N-termini but distinct C-termini.

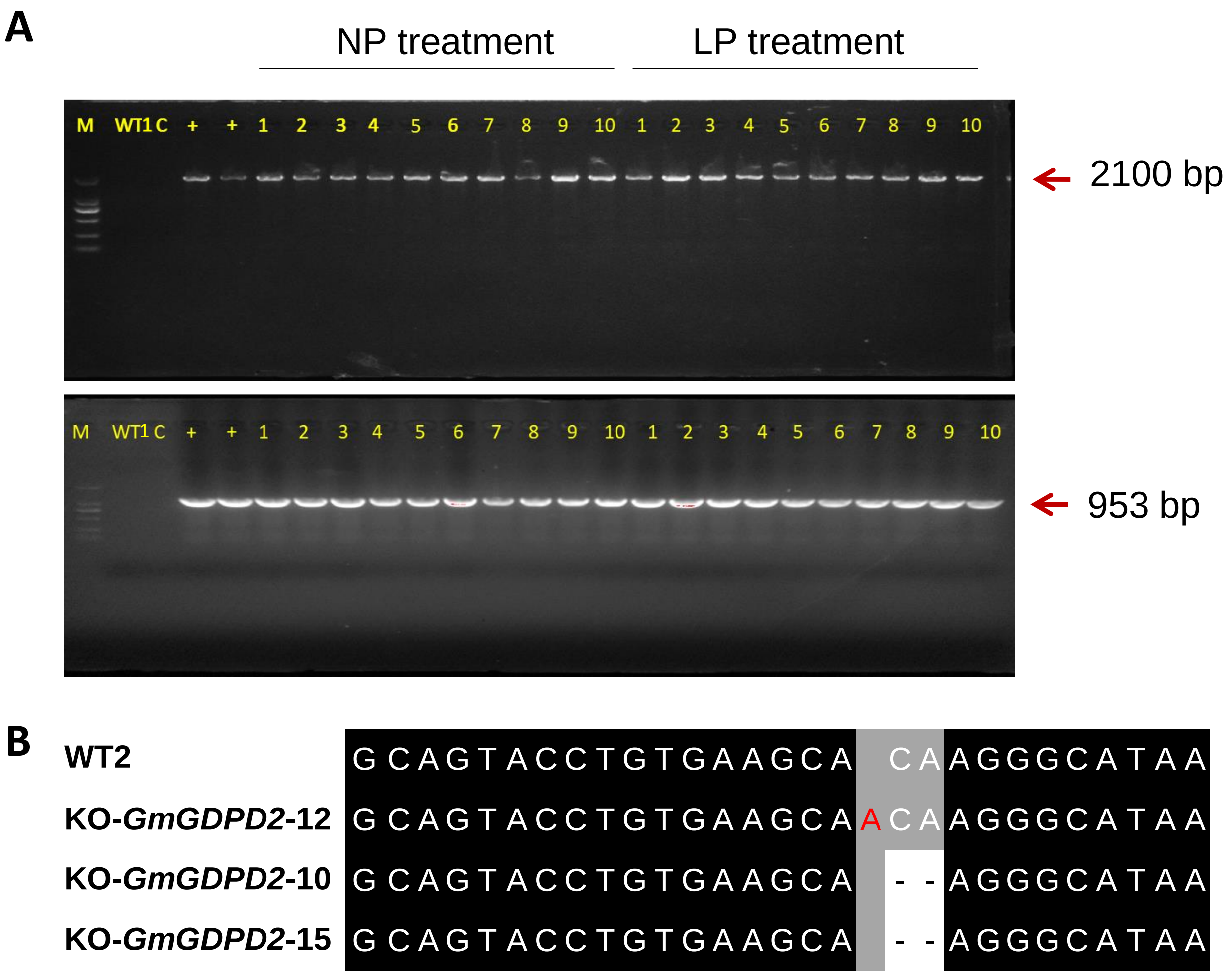

Supplemental Figure S11. Identification of positive *GmGDPD2* transgenic lines. (Supports Fig. 2) A, PCR results confirming the positive transgenics for overexpression of *GmGDPD2*. **B**, Sanger sequencing results confirming the positive transgenics for Cas9-mediated editing of *GmGDPD2*. WT1, Jack; WT2, W82.

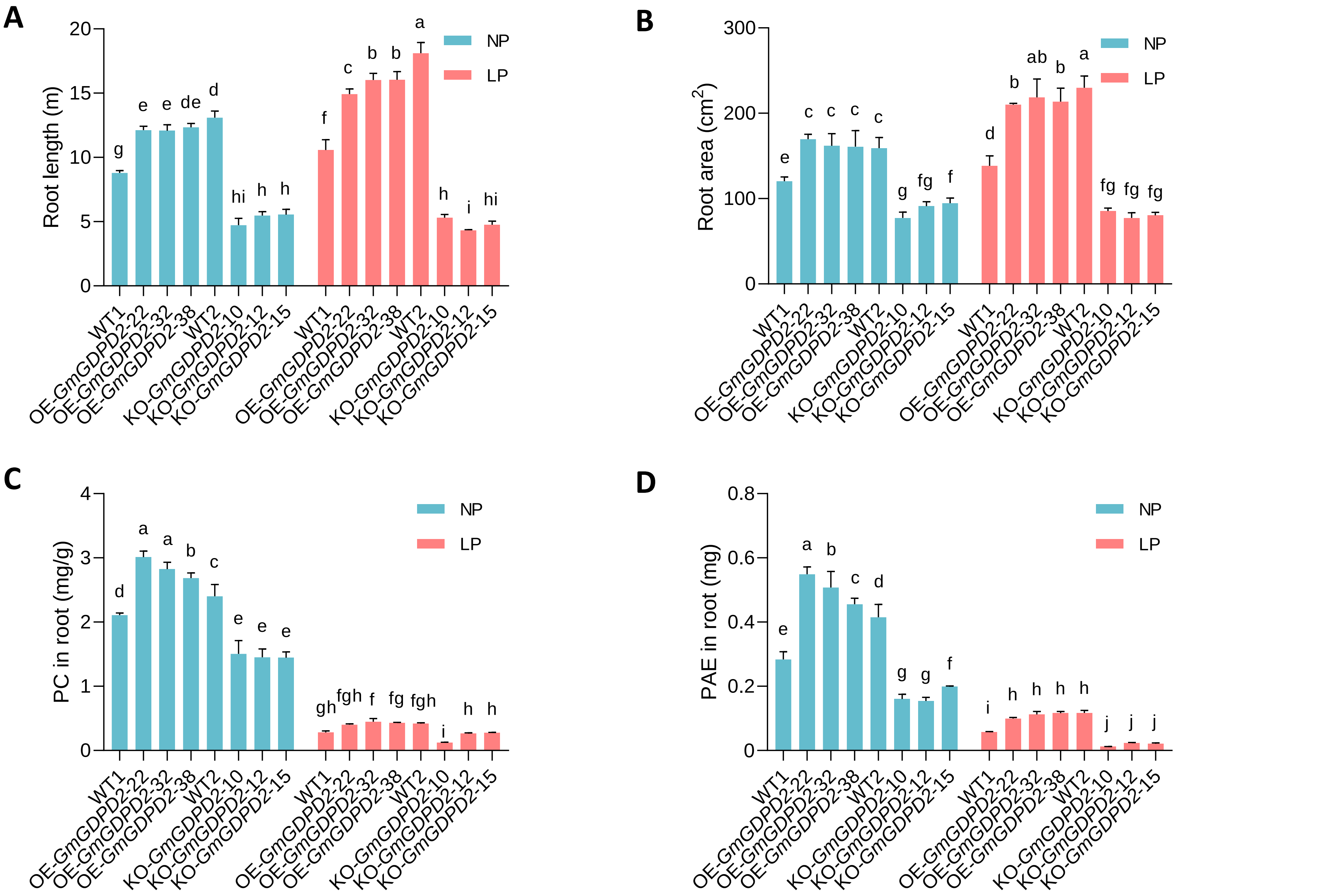

Supplemental Figure S12. Root traits between the *GmGDPD2* overexpression and knockout plants with the wild types under NP or LP conditions for 7 days. (Supports Fig. 2C-F) Root length (**A**), Root area (**B**), PC in root (**C**), and PAE in root (**D**) of two wild types, *GmGDPD2* overexpression (OE) and knockout (KO) lines. LP, low P supply (5 μM, P); NP, normal P supply (500 μM, P); WT1, Jack; WT2, W82. The phenotype of each line for each treatment was evaluated with three plants. Trait values are shown as the mean ± SD (standard deviation). Means with different letters are significantly different (one-way ANOVA, Duncan, $P \leq 0.05$). PC, Pi concentration; PAE, Pi absorption efficiency concentration.

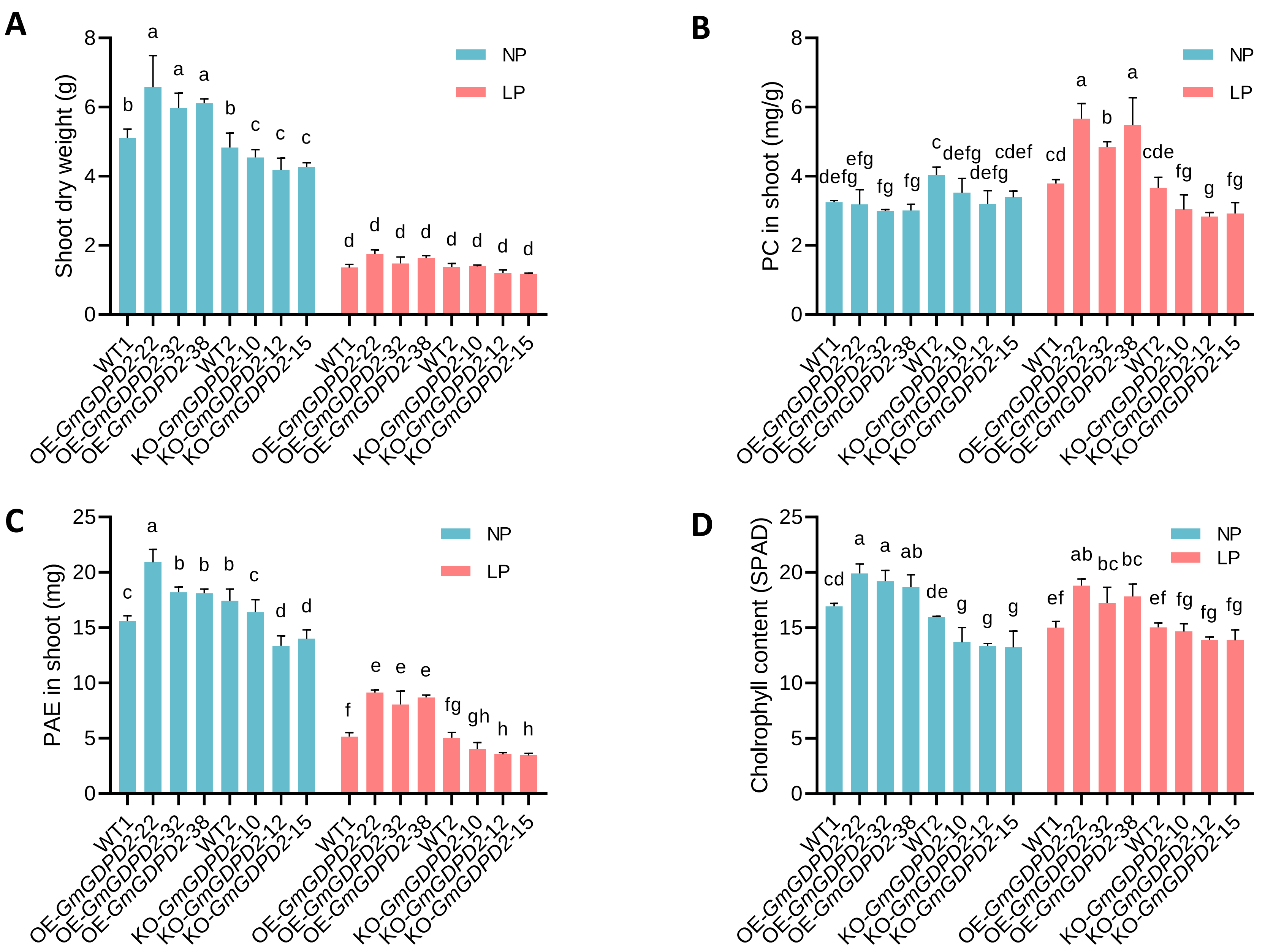

Supplemental Figure S13. Shoot traits between the *GmGDPD2* overexpression and knockout plants with the wild types under NP or LP conditions in the plot. (Supports Fig. 2H-K) Shoot day weight (**A**), PC in shoot (**B**), PAE in shoot (**C**), and cholrophyll content (SPAD) (**D**) of two wild types, *GmGDPD2* overexpression (OE) and knockout (KO) lines. LP, low P supply (5 μM, Pi); NP, normal P supply (500 μM, Pi); WT1, Jack; WT2, W82. The phenotype of each line for each treatment was evaluated with three plants. Trait values are shown as the mean ± SD (standard deviation). Means with different letters are significantly different (one-way ANOVA, Duncan, P ≤ 0.05). PC, Pi concentration; PAE, Pi absorption efficiency concentration.

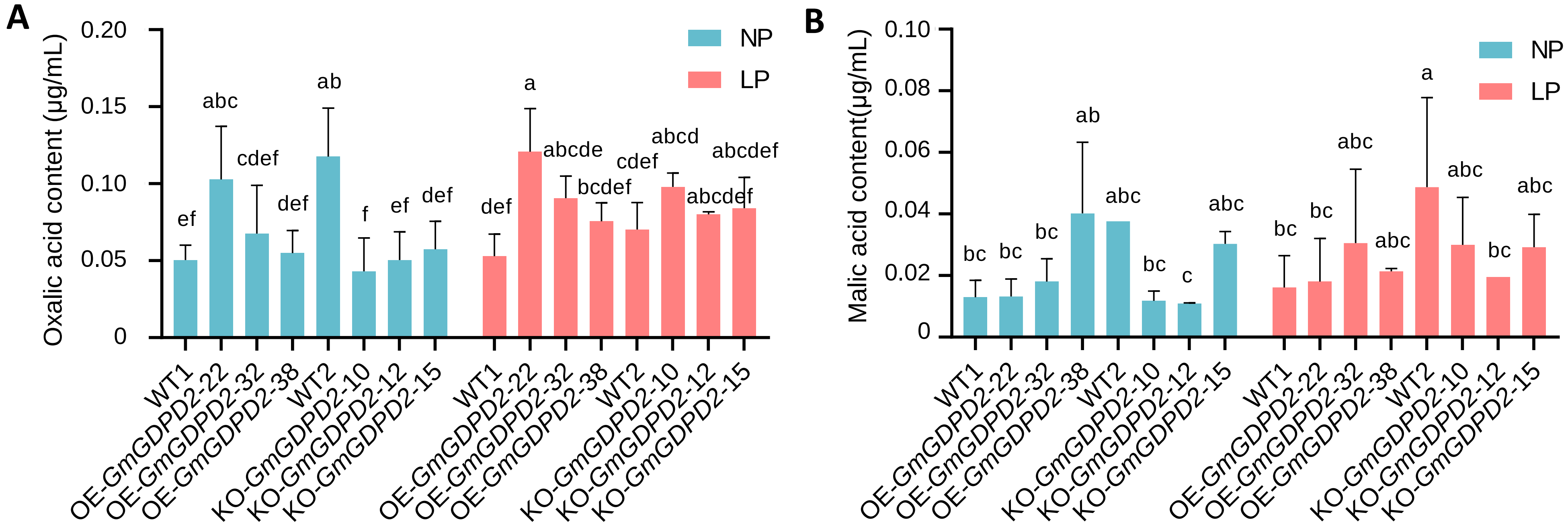

Supplemental Figure S14. Oxalic acid and malic acid secreted from the root of *GmGDPD2* overexpression and knockout plants, and the wild types after NP and LP treatment for 8 days. (Supports Fig. 7) Oxalic acid content (**A**) and Malic acid content (B) of two wild types, *GmGDPD2* overexpression (OE) and knockout (KO) lines. LP, low P supply (5 μM, Pi); NP, normal P supply (500 μM, Pi); WT1, Jack; WT2, W82. The phenotype of each line for each treatment was evaluated with three plants. Trait values are shown as the mean ± SD (standard deviation). Means with different letters are significantly different (one-way ANOVA, Duncan, P ≤ 0.05).

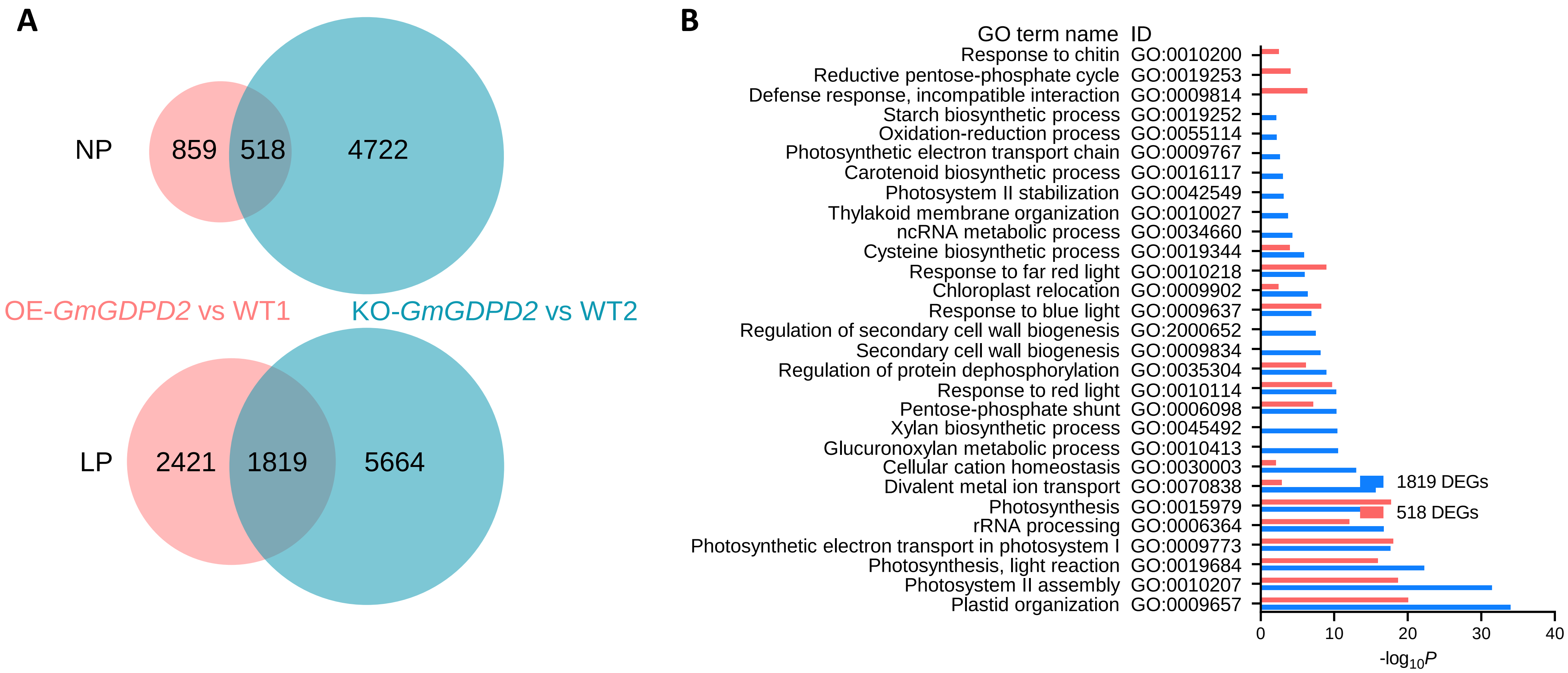

Supplemental Figure S15. **Gene expression comparison between *GmGDPD2* overexpression or knockout roots with corresponding wild types under NP or LP conditions for 14 days. (Supports Fig. 2) A**, Venn Diagram showing overlapping genes between DEGs from 'OE-*GmGDPD2* vs WT1' and DEGs from 'KO-*GmGDPD2* vs WT2'. WT1, Jack; WT2, W82; OE-*GmGDPD2*, overexpressing *GmMyb73* plants; KO-*GmGDPD2*, knock out *GmGDPD2* plants; DEGs, differentially expressed genes. LP, low P supply (5 μM, Pi); NP, normal P supply (500 μM, Pi). **B**, GO enrichment analysis for the overlapped 518 and 1819 DEGs under NP and LP conditions, respectively. Red bars represent the 518 DEG under NP condition, blue bars represent 1819 DEGs under LP condition.

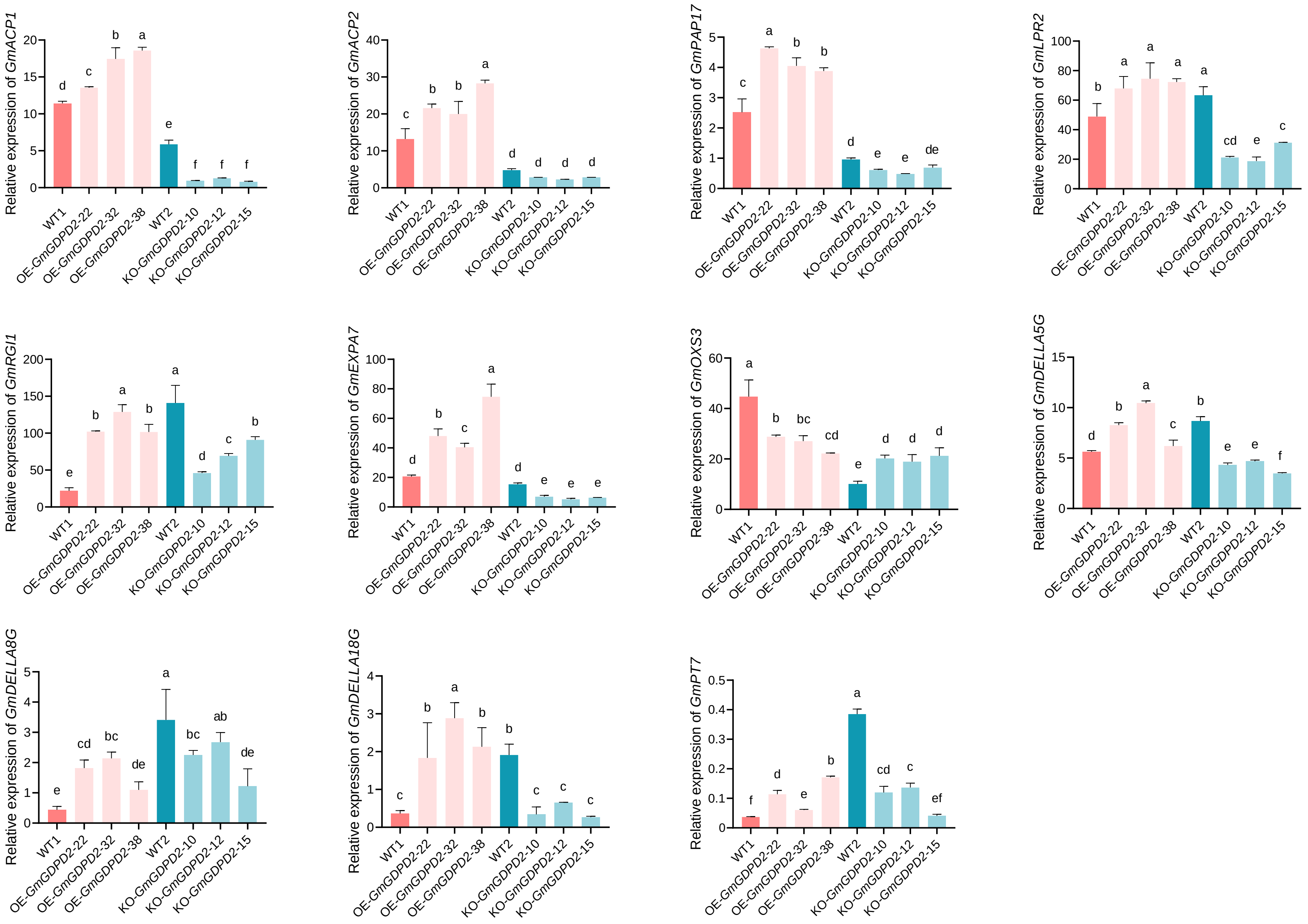

Supplemental Figure S16. Gene expression pattens among *GmGDPD2* overexpression or knockout roots with corresponding wild types under NP condition. (Supports Fig. 7) NP, normal P supply (500 μM, Pi); WT1, Jack; WT2, W82. Selected genes are *GmACP1* (Glyma.08G195100), *GmACP2* (Glyma.08G195000), *GmPAP17* (Glyma.08G056400), *GmLPR2* (Glyma.10G263800), *GmRG1* (Glyma.01G062900), *GmEXPA7* (Glyma.11G027600), *GmOXS3* (Glyma.01G006000), *GmDELLA* (5G, Glyma.05G140400; 8G, Glyma.08G095800; 18G, Glyma.18G040000), and *GmPT7* (Glyma.10G186500). Trait values are shown as the mean ± SD (standard deviation), and each line contains three individuals. Means with different letters are significantly different (one-way ANOVA, Duncan, P ≤ 0.05).

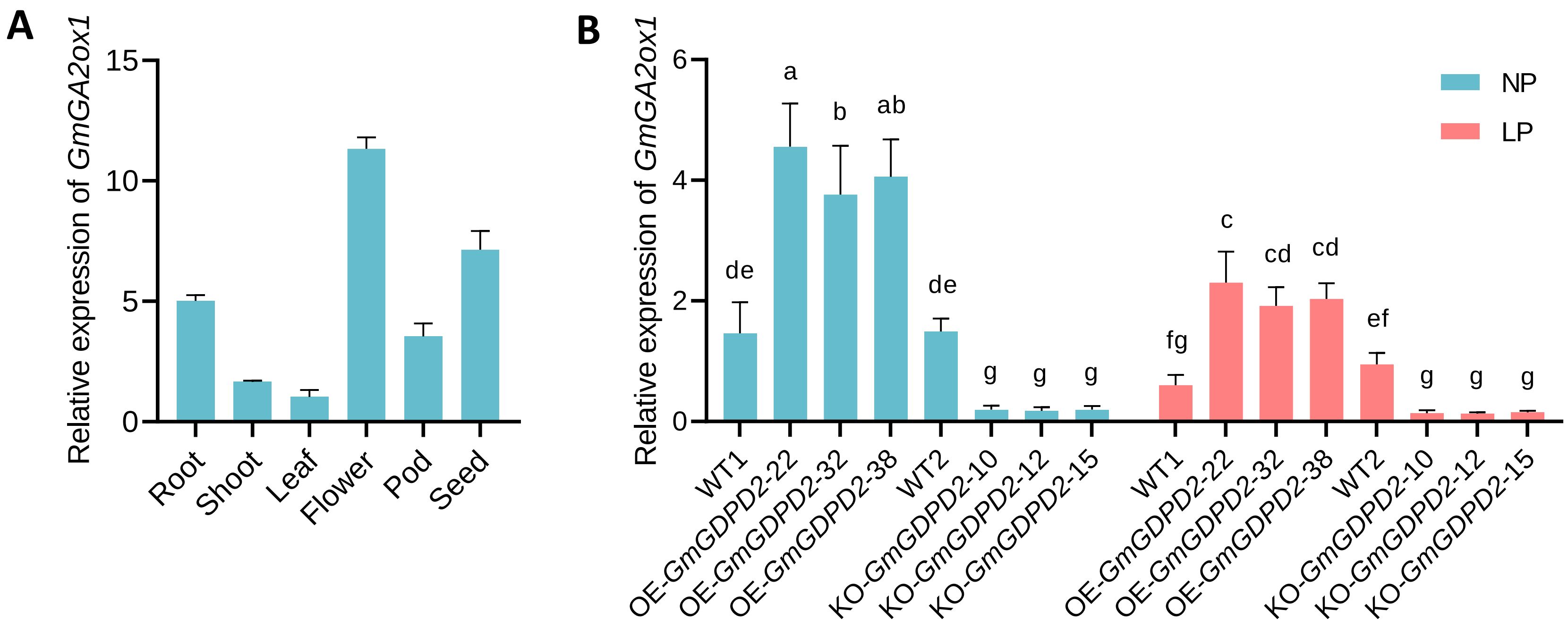

Supplemental Figure S17. **Expression levels of *GmGA2ox1*. (Supports Fig. 5) A**, Expression of *GmGDPD2* in soybean hairy roots for WT1, overexpressing *GmMyb73* (OE-*GmMyb73*) plants and silencing *GmMyb73* (Ri-*GmMyb73*) plants after NP or LP treatments for 10 days. **A**, Expression of *GmGA2ox1* for W82 in six tissues under NP conditions. **B**, Expression levels of *GmGA2ox1* in *GmGDPD2* overexpression (OE-*GmGDPD2*) plants, knockout (KO-*GmGDPD2*) plant roots after NP or LP treatments for 10 days. LP, low P supply (5 μM, Pi); NP, normal P supply (500 μM, Pi); WT1, Jack; WT2, W82. Expression data of each line for each treatment was based on three plants. Trait values are shown as the mean ± SD (standard deviation). Means with different letters are significantly different (one-way ANOVA, Duncan, P ≤ 0.05).

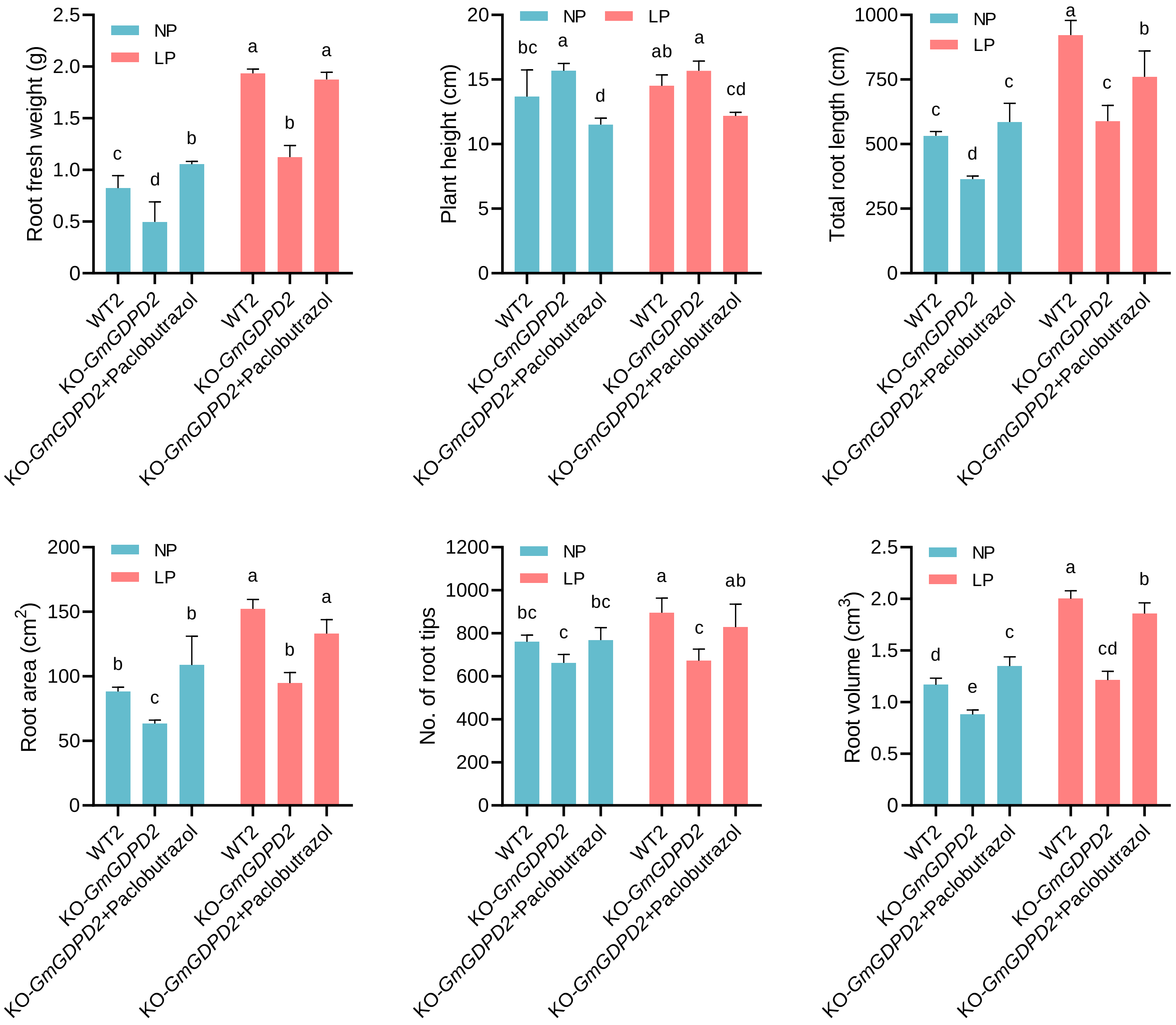

Supplemental Figure S18. Root related traits of WT2, KO-*GmGDPD2* and KO-*GmGDPD2* with Paclobutrazol treatment under NP and LP condition for 8 Days. (Supports Fig. 4F) LP, low P supply (5 μM, Pi); NP, normal P supply (500 μM, Pi); WT2, W82. Paclobutrazol concentration: 30 mg/L. KO-*GmGDPD2* represents three Gm*GDPD2* knockout lines, the phenotype of each Gm*GDPD2* line for each treatment was evaluated with one plant, WT2 was evaluated with three plants. Trait values are shown as the mean ± SD (standard deviation). Means with different letters are significantly different (one-way ANOVA, Duncan, P ≤ 0.05).

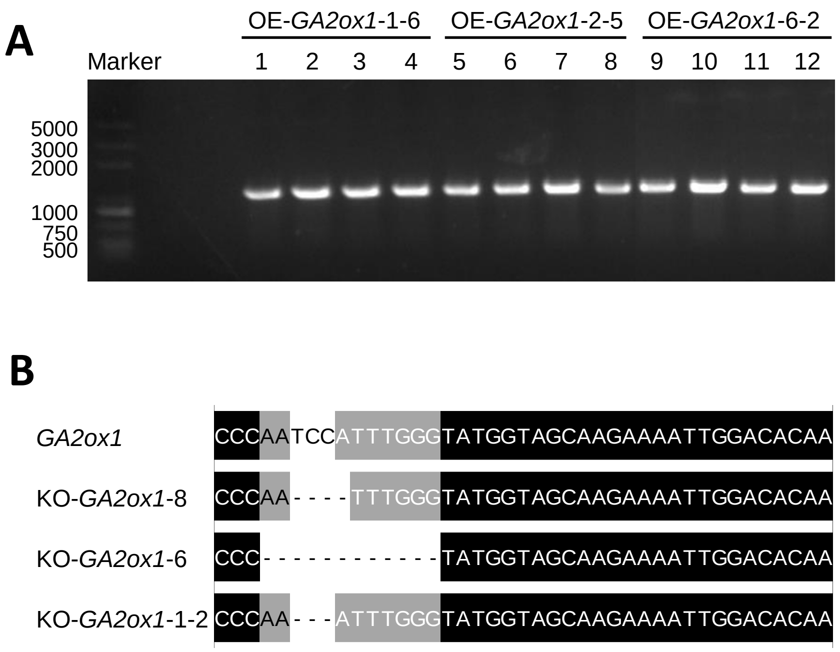

Supplemental Figure S19. Identification of positive *GmGA2ox1* transgenic lines. (Supports Fig. 5) A, PCR results confirming the positive transgenics for overexpression of *GmGA2ox1*. **B**, Sanger sequencing results confirming the positive transgenics for Cas9-mediated editing of *GmGA2ox1*.

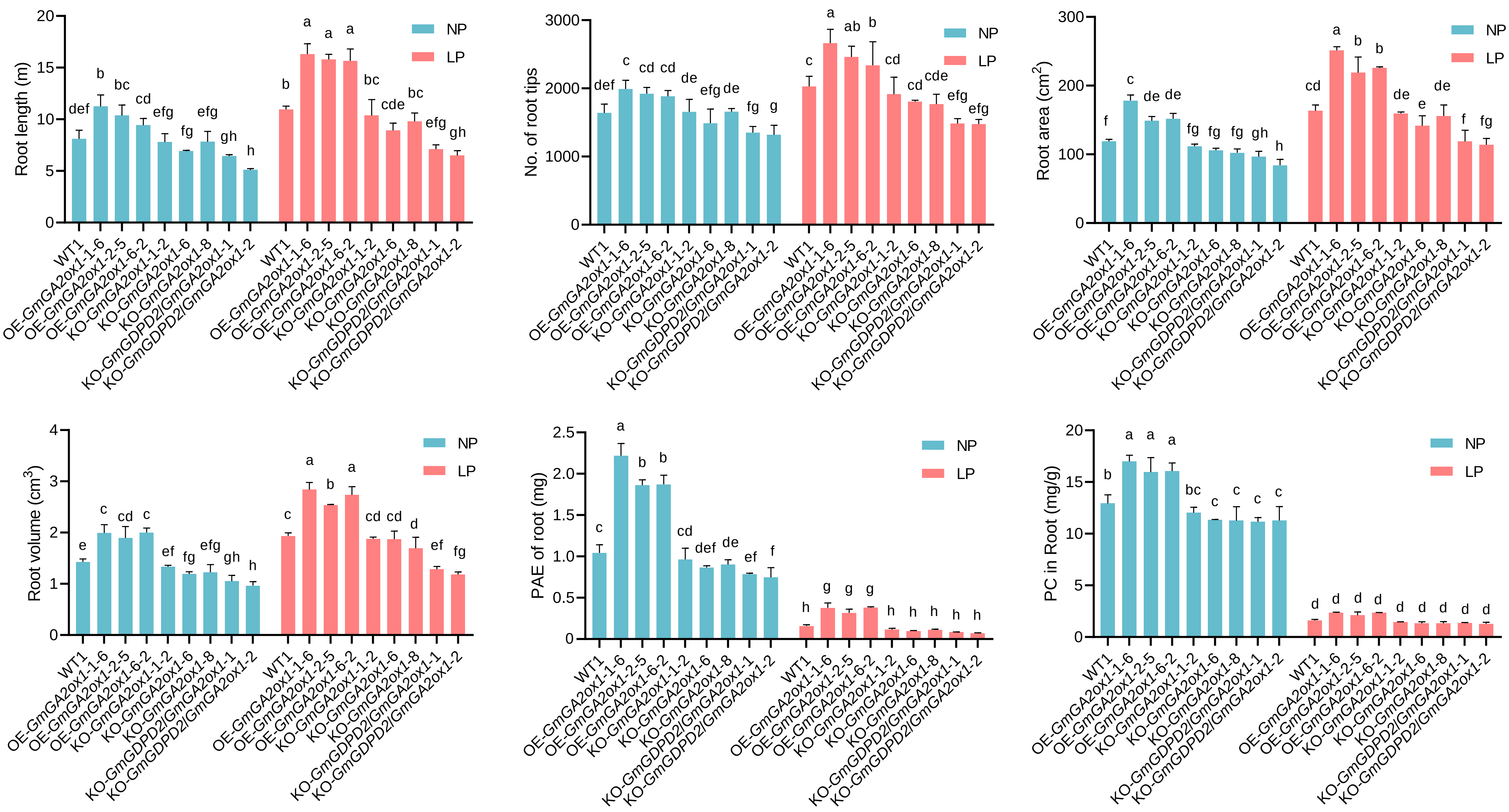

Supplemental Figure S20. Comparisons of root and Pi efficiency traits between transgenic plants and the wild type plants after NP or LP treatments for 10 days. (Supports Fig. 5B-G) LP, low P supply (5 μM, Pi); NP, normal P supply (500 μM, Pi); WT1, Jack; PC, Pi concentration; PAE, Pi absorption efficiency concentration. The phenotype of each line for each treatment was evaluated with three plants. Trait values are shown as the mean ± SD (standard deviation). Means with different letters are significantly different (one-way ANOVA, Duncan, P ≤ 0.05)..

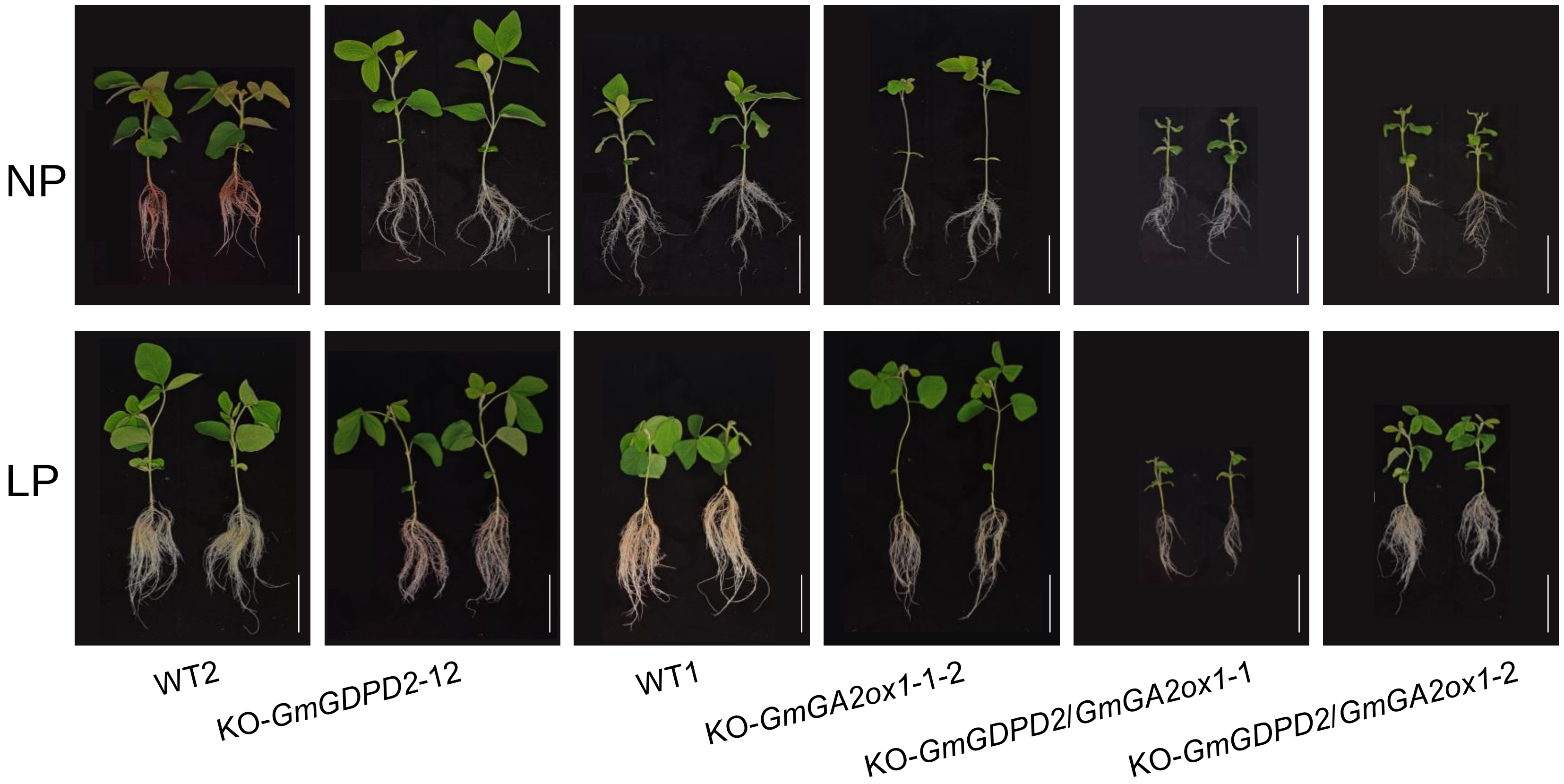

Supplemental Figure S21. Morphologic illustration of WT1, WT2, knockout (KO)-*GmGDPD2* plants, KO-*GmGA2ox1* plants and KO-*GmGA2ox1*/*GmGA2ox1* (double knockout) plants after NP or LP treatments for 9 days. (Supports Fig. 5A) Bars = 5 cm. LP, low P supply (5 μM, Pi); NP, normal P supply (500 μM, Pi); WT1, Jack; WT2, W82.

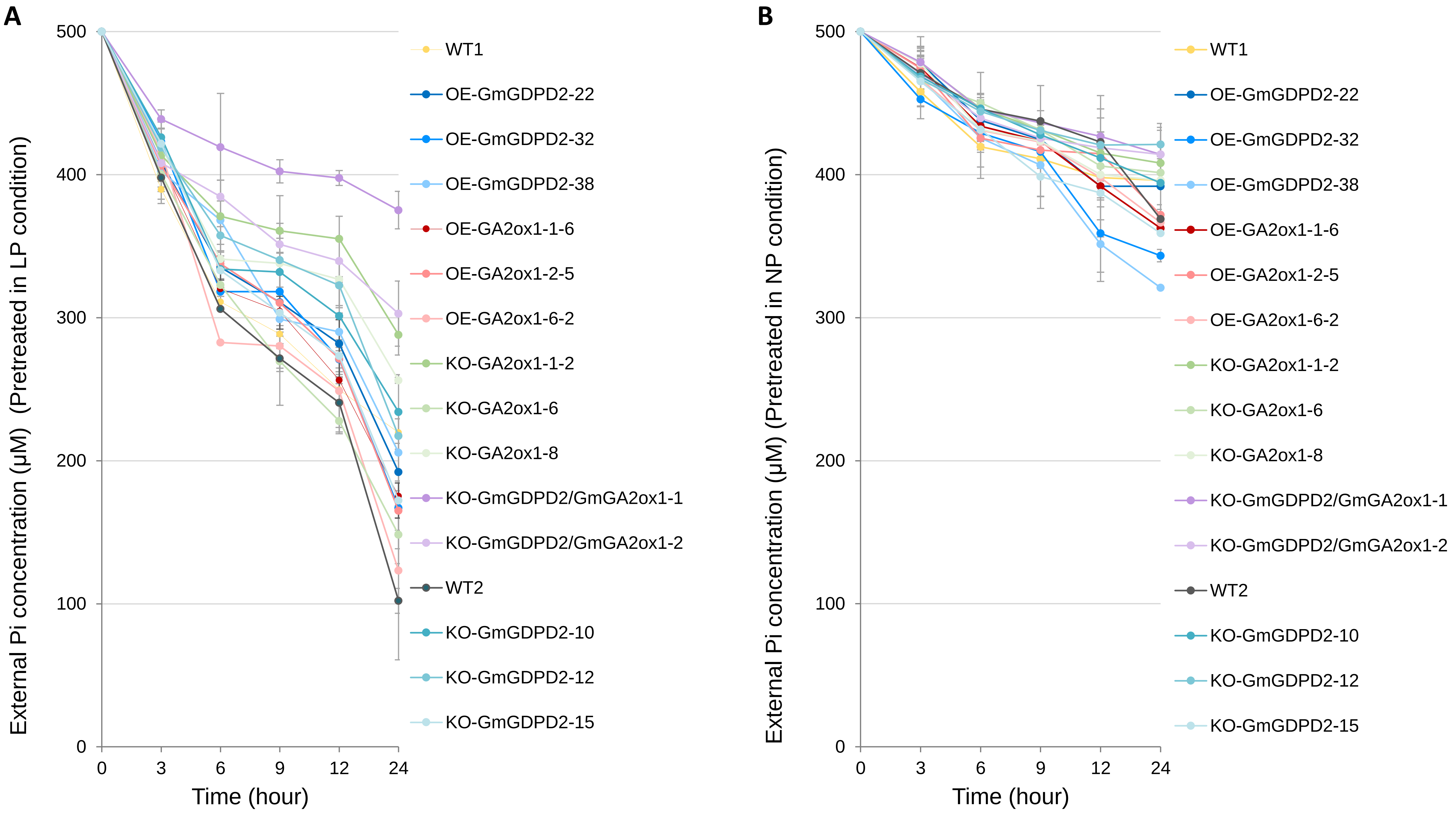

Supplemental Figure S22. Pi-uptake rate measurement. (Supports Fig. 5H-I) Seedlings with unfolded cotyledons were pretreated in LP (**A**) and NP (**B**) condition for 9 days, and then transferred to deionized water for 6 hours, then transferred to black plastic cup in NP condition. LP, low P supply (5 μM, Pi); NP, normal P supply (500 μM, Pi); WT1, Jack; WT2, W82. Evaluation of each line for each treatment was based on three plants. Trait values are shown as the mean ± SD (standard deviation).

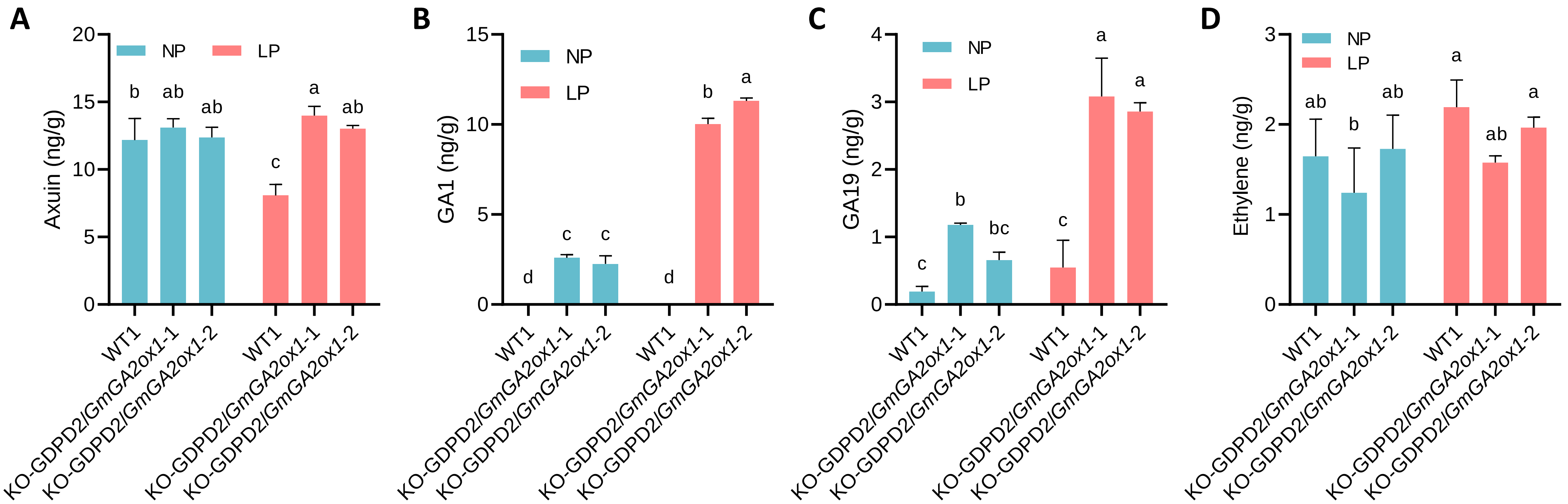

Supplemental Figure S23. Phytohormone changes in roots of KO-*GmGA2ox1*/*GmGA2ox1* plants after NP or LP conditions for 14 days. (Supports Fig. 5J-M) Auxin (**A**), GA1 (**B**), GA19 (**C**), and ethylene (**D**) of wild type WT1 and KO-*GmGA2ox1*/*GmGA2ox1* (double knockout) plants. LP, low P supply (5 μM, Pi); NP, normal P supply (500 μM, Pi); WT1, Jack. Evaluation of each line for each treatment was based on three plants. Trait values are shown as the mean ± SD (standard deviation). Means with different letters are significantly different (one-way ANOVA, Duncan, $P \leq 0.05$).

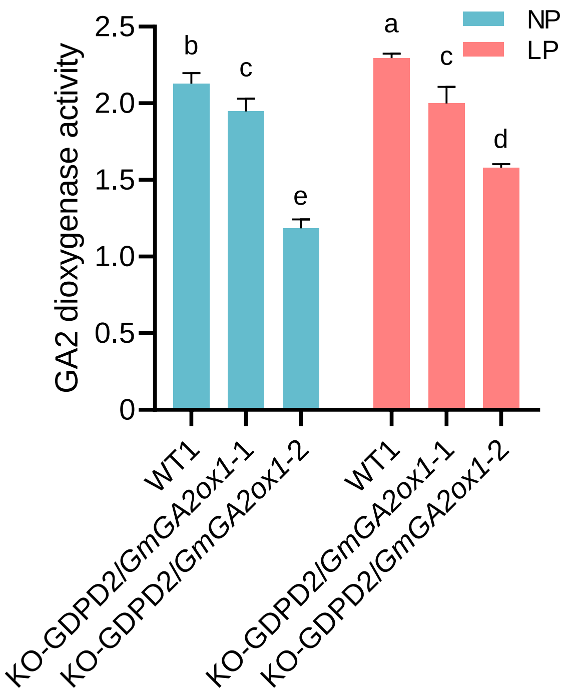

Supplemental Figure S24. GA2 dioxygenase activity in roots of KO-*GmGA2ox1*/*GmGA2ox1* plants after NP or LP treatments for 7days. (Supports Fig. 5N) LP, low P supply (5 μM, Pi); NP, normal P supply (500 μM, Pi); WT1, Jack. Evaluation of each line for each treatment was based on three plants. Trait values are shown as the mean ± SD (standard deviation). Means with different letters are significantly different (one-way ANOVA, Duncan, P ≤ 0.05).

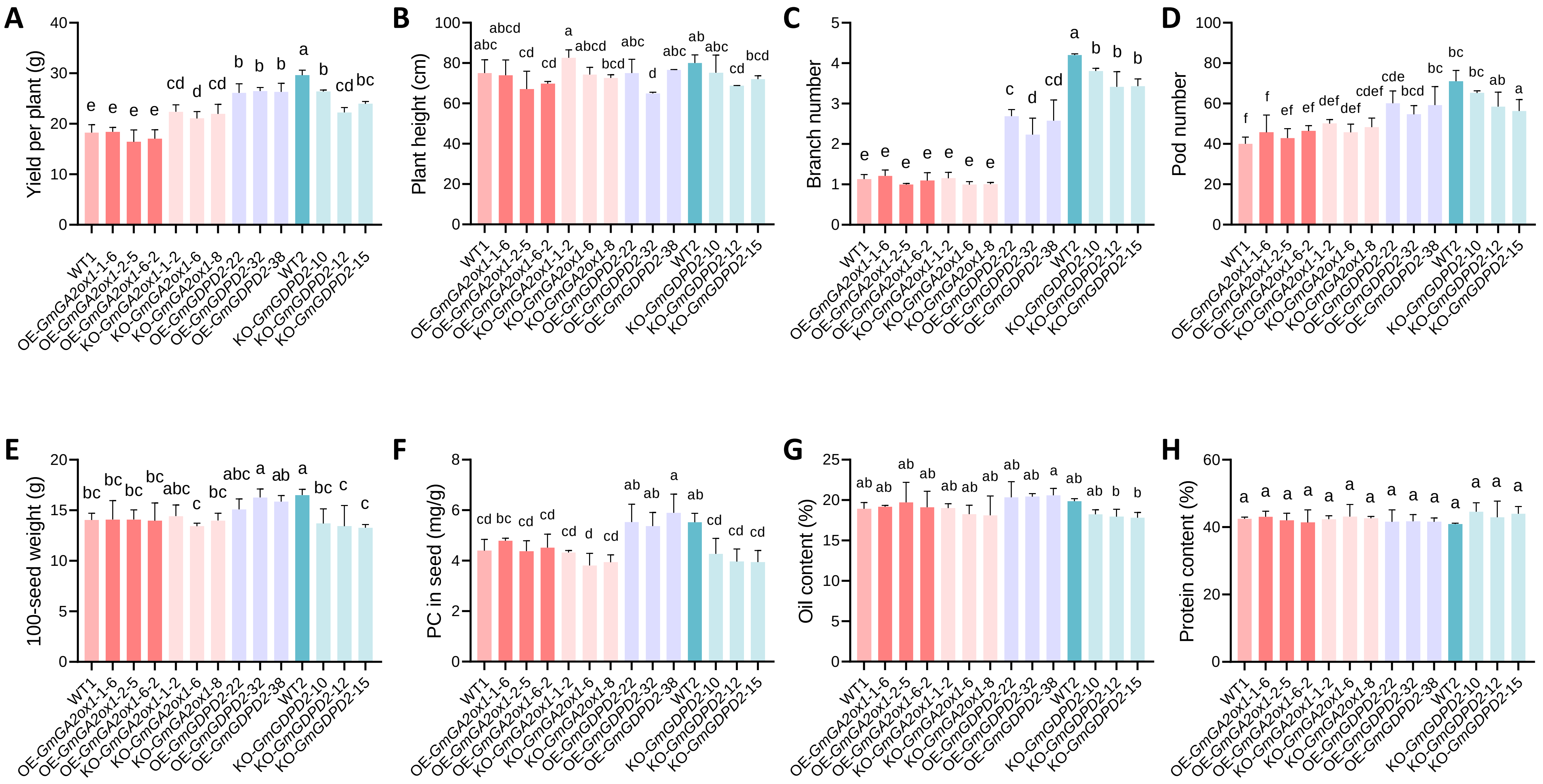

Supplemental Figure S25. Phenotypic comparison between the *GmGDPD2* and *GmGA2ox1* transgenic lines with the respective wild types after maturation in the greenhouse. (Supports Fig. 6B-I) Yield per plant (A), plant height (B), Branch number (C), pod number (D), 100-seed weight (E), PC in seed (F), oil content in seed (G), protein content in seed (H) of two wild types, *GmGDPD2* overexpression (OE) and knockout (KO) lines, *GmGA2ox1* overexpression (OE) and knockout (KO) lines. PC, Pi concentration; WT1, Jack; WT2, W82. Evaluation of each line for each treatment was based on three plants. Trait values are shown as the mean ± SD (standard deviation). Means with different letters are significantly different (one-way ANOVA, Duncan, P ≤ 0.05).

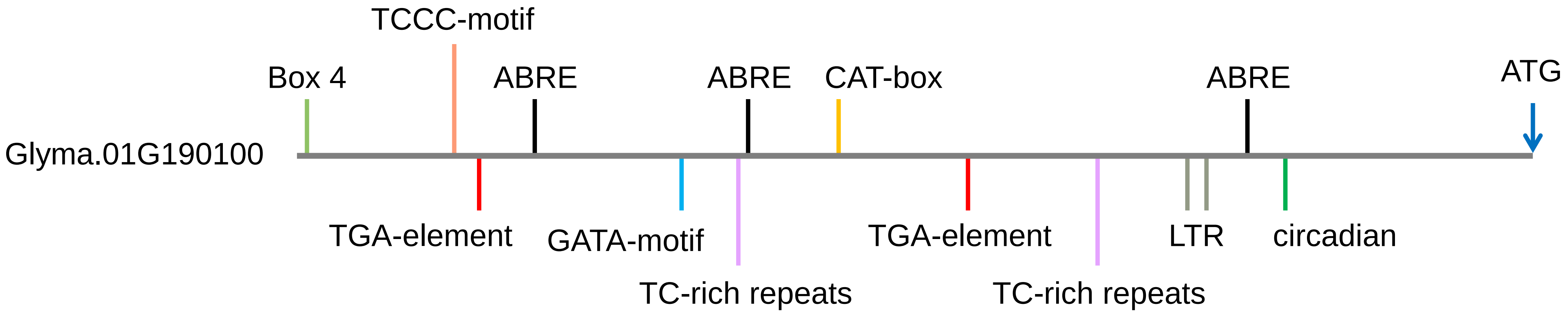

Supplemental Figure S26. Predicted cis-elements in *GmMyb73* promoter. (Supports Fig. 7) Box4 (ATTAAT), TCCC-motif (TCTCCCT), GATA-motif (GATAGGA), and TC-rich repeats (ATTCTCTAAC) are cis-acting regulatory elements involving in light responsiveness; TGA-element (AACGAC), auxin regulatory element; ABRE (ACGTG), cis-acting element involving in abscisic acid responsiveness; CAT-box (GCCACT), cis-acting regulatory element relates to meristem expression； LTR (CCGAAA), cis-acting element involving in low-temperature responsiveness; circadian (CAAAGATATC), cis-acting regulatory element involving in circadian control.

Supplemental Table S1. Descriptive statistical results for traits related to phosphate (Pi) efficiency in 127 recombinant inbred lines (RILs) and their parents in experiments conducted in 2018 and 2019.

Trait	Treatment	Year/Environment	Mean±SD	Range	G[a]	R[b]	Y[c]	G × E[d]	Dongnong50	Williams82
RRA	-P/+P	2018	1.53±0.43	0.76-3.55	**	ns	ns	ns	1.23	1.66
		2019	1.43±0.40	0.75-3.54	**	ns			1.21	1.69
RRV	-P/+P	2018	1.42±0.32	0.77-3.32	**	ns	ns	ns	1.25	1.55
		2019	1.34±0.36	0.73-3.50	**	ns			1.05	1.48
RRN	-P/+P	2018	1.42±0.42	0.48-3.77	**	*	ns	ns	1.21	1.69
		2019	1.32±0.40	0.48-3.45	**	ns			1.08	1.56
RRL	-P/+P	2018	1.67±0.63	0.93-3.73	**	*	*	*	1.23	1.81
		2019	1.51±0.45	0.76-3.55	**	ns			1.24	1.78

Statistically significant differences are indicated with * ($P < 0.05$) and ** ($P < 0.01$); ns: not significant; RRA: the relative values of root surface area for LP/NP, RRV: the relative values of root volume for LP/NP, RRN: the relative values of the number of root tips for LP/NP, RRL: the relative values of root length for LP/NP. [a] genotype; [b] replication; [c] year.

Supplemental Table S2. Information for the 7 QTL identified by OTL mapping based on DW RIL population.

QTL	Trait Name	Chr.	Position (CM)	Marker		LOD	PVE (%)	Physical position*		LP related gene that closed to the QTL	
				left	Right			Start	End	Gene name	Reference
qPE2-1	2018RRA	2	19	Gm02_19	Gm02_20	3.2095	11.0552	4,090,344	4,375,910		
qPE2-2	2019RRL	2	137	Gm02_131	Gm02_132	3.016	7.4811	46,654,290	46,984,625	*GmEIL4/EIN3*	Yang et al., 2023
qPE6	2018RRN	6	63	Gm06_40	Gm06_41	2.5902	8.4667	12,979,788	13,239,162		
qPE10-1	2019RRA	10	69	Gm10_61	Gm10_62	2.9513	6.9297	39,869,859	40,166,609	*GmPHT1;2*<br>*GmPHT1;6*	Guo et al., 2022<br>Song et al., 2014
qPE11	2018RRV	11	132	Gm11_72	Gm11_73	2.5235	10.8986	29,167,970	31,837,593		
qPE13-1	2019RRA	13	54	Gm13_51	Gm13_52	3.0363	7.1861	25,379,570	26,114,215	*GmERF1*	Wang et al., 2023
	2019RRL	13	56	Gm13_51	Gm13_52	4.6896	13.3863	25,379,570	26,114,215		
qPE19-1	2018RRA	19	27	Gm19_18	Gm19_19	2.6258	9.0426	6,672,601	8,791,935		
	2018RRN	19	27	Gm19_18	Gm19_19	3.7524	12.7507	6,672,601	8,791,935		
	2018RRL	19	27	Gm19_18	Gm19_19	6.2429	19.5433	6,672,601	8,791,935		
	2019RRV	19	27	Gm19_18	Gm19_19	5.5753	22.087	6,672,601	8,791,935		
	2019RRA	19	28	Gm19_21	Gm19_22	9.0267	23.9679	9,276,985	28,418,279		
	2019RRN	19	28	Gm19_21	Gm19_22	4.0134	13.7399	9,276,985	28,418,279		
	2019RRL	19	28	Gm19_21	Gm19_22	8.7297	24.0646	9,276,985	28,418,279		

*Physical position based on Wm82.a2.v1 genome.

Supplemental Table S3. Descriptive statistical results for traits related to phosphate (Pi) efficiency in 367 soybean accessions in experiments conducted in 2018 and 2019.

Trait	Treatment	Year/Environment	Mean±SD	Range	G[a]	R[b]	Y[c]	G × E[d]
RRA	-P/+P	2018	1.23±0.64	0.11-3.70	**	ns	ns	ns
		2019	1.21±0.64	0.12-3.91	**	ns		
RRD	-P/+P	2018	1.09±0.61	0.32-4.08	**	ns	ns	ns
		2019	1.07±0.60	0.32-3.88	**	ns		
RRV	-P/+P	2018	1.45±0.72	0.12-3.96	**	*	ns	*
		2019	1.42±0.71	0.11-3.96	**	ns		
RRT	-P/+P	2018	1.43±0.77	0.03-3.97	**	*	*	*
		2019	1.39±0.76	0.03-3.82	**	*		
RRN	-P/+P	2018	1.45±0.65	0.12-3.59	**	ns	*	*
		2019	1.42±0.65	0.13-3.40	**	ns		
RPC	-P/+P	2018	0.45±0.22	0.12-1.10	**	ns	ns	ns
		2019	0.44±0.22	0.10-1.17	**	ns		
RPAE	-P/+P	2018	0.63±0.51	0.06-3.01	**	ns	ns	ns
		2019	0.62±0.49	0.06-2.89	**	ns		

Statistically significant differences are indicated with * ($P < 0.05$) and ** ($P < 0.01$); ns: not significant; RRA: the relative values of root surface area for LP/NP, RRD: the relative values of root diameter for LP/NP, RRV: the relative values of root volume for LP/NP, RRN: the relative values of the number of root tips for LP/NP, RRL: the relative values of root length for LP/NP, RPC: the relative values of root Pi concentration for LP/NP, RPAE: the relative values of Pi absorption efficiency for LP/NP. a genotype; b replication; c year; d interaction between genotype and environments.

Supplemental Table S4. Pearson correlation among the LP-related root and Pi efficiency traits.

	RRA	RRD	RRL	RRN	RRV	RPC
RRD	0.39868374					
RRL	0.639612186	0.489246291				
RRN	0.362417749	0.430937038	0.480366521			
RRV	0.390759412	0.609239152	0.560164696	0.367991649		
RPC	0.950013794	0.401743182	0.650922704	0.393808968	0.407259584	
RPAE	0.749072742	0.742407718	0.716864737	0.518870191	0.737265479	0.77588635

RRA: the relative values of root surface area for LP/NP, RRD: the relative values of root diameter for LP/NP, RRV: the relative values of root volume for LP/NP, RRN: the relative values of the number of root tips for LP/NP, RRL: the relative values of root length for LP/NP, RPC: the relative values of root P concentration for LP/NP, RPAE: the relative values of P absorption efficiency for LP/NP.

Supplemental Table S5. Information of nine QTL identified through GWAS.

QTL	Traits	Leader SNPs	Chr.	Position	Ref	Alt	R^2	*P*.MLM	LP related gene that closed to the QTL	
									Gene name	Reference
qPE2-3	RRD	AX-93982911	2	41,387,979	T	G	0.1092	3.02849E-07		
qPE5	RRD	AX-94016860	5	32,599,883	G	A	0.3513	2.19341E-07		
qPE8	RRA, RRD, RPC, RPAE	AX-93750137	8	3,741,107	T	A	0.0721	2.48145E-09	*GmPAP17*	Xu et al., 2022
qPE10-2	RRA, RRL, RPC	AX-94076203	10	31,288,055	A	G	0.1977	2.5342E-09	*GmEXPB2*<br>*GmPHT1;2*	Guo et al., 20121<br>Guo et al., 2022
qPE13-2	RRD, RPAE	AX-93808626	13	15,487,028	C	A	0.3028	3.8397E-07		
qPE14	RRN	AX-94129026	14	46,936,977	T	C	0.096	1.05001E-07	*GmETO1*	Zhang et al., 2020
qPE17	RRD	AX-93861026	17	12,280,179	C	A	0.3435	1.57607E-06	*GmEXLB1*	Kong et al., 2019
qPE19-2	RRA, RRD, RRL, RRN, RRV, RPC, RPAE	AX-94187201	19	15,989,213	G	T	0.432	3.73367E-11		
qPE20	RRD	AX-94203530	20	30,996,793	A	G	0.3863	1.0053E-07		

*Physical position based on Wm82.a4 genome. RRA: the relative values of root surface area for LP/NP, RRD: the relative values of root diameter for LP/NP, RRV: the relative values of root volume for LP/NP, RRN: the relative values of the number of root tips for LP/NP, RRL: the relative values of root length for LP/NP, RPC: the relative values of root P concentration for LP/NP, RPAE: the relative values of P absorption efficiency for LP/NP.

Supplemental Table S6. Identification of *GmGDPD2* Hap5 through functional KSAP marker in 79 soybean lines.

Accession	Haplotype	Result of KSAP marker	Hap according to the KSAP
NJAU_C008	Hap5	TGA:TGA	Hap5
NJAU_C040	Hap5	TGA:TGA	Hap5
NJAU_C100	Hap5	TGA:TGA	Hap5
NJAU_C210	Hap5	TGA:TGA	Hap5
NJAU_C229	Hap5	TGA:TGA	Hap5
NJAU_C211	Hap5	TGA:TGA	Hap5
NJAU_C222	Hap5	TGA:TGA	Hap5
NJAU_W012	Hap5	TGA:TGA	Hap5
NJAU_W079	Hap5	TGA:TGA	Hap5
NJAU_W086	Hap5	TGA:TGA	Hap5
NJAU_W089	Hap5	TGA:TGA	Hap5
NJAU_C003	Hap1	-:-	Non-Hap5
NJAU_C004	Hap1	-:-	Non-Hap5
NJAU_C005	Hap1	-:-	Non-Hap5
NJAU_C006	Hap1	-:-	Non-Hap5
NJAU_C009	Hap1	-:-	Non-Hap5
NJAU_C011	Hap1	-:-	Non-Hap5
NJAU_C012	Hap1	-:-	Non-Hap5
NJAU_C013	Hap1	-:-	Non-Hap5
NJAU_C014	Hap1	-:-	Non-Hap5
NJAU_C099	Hap1	-:-	Non-Hap5
NJAU_C101	Hap1	-:-	Non-Hap5
NJAU_C219	Hap1	-:-	Non-Hap5
NJAU_C234	Hap1	-:-	Non-Hap5
NJAU_W006	Hap1	-:-	Non-Hap5
NJAU_W008	Hap1	-:-	Non-Hap5
NJAU_C010	Hap2	-:-	Non-Hap5
NJAU_C037	Hap2	-:-	Non-Hap5
NJAU_C050	Hap2	-:-	Non-Hap5
NJAU_C083	Hap2	-:-	Non-Hap5
NJAU_C134	Hap2	-:-	Non-Hap5
NJAU_C259	Hap2	-:-	Non-Hap5
NJAU_W025	Hap2	-:-	Non-Hap5
NJAU_W036	Hap2	-:-	Non-Hap5
NJAU_W096	Hap2	-:-	Non-Hap5
NJAU_W009	Hap3	-:-	Non-Hap5
NJAU_W018	Hap3	-:-	Non-Hap5
NJAU_W023	Hap3	-:-	Non-Hap5
NJAU_W030	Hap3	-:-	Non-Hap5
NJAU_W050	Hap3	-:-	Non-Hap5

Continued

**Accession**	**Haplotype**	**Result of KSAP marker**	**Hap according to the KSAP**
NJAU_W085	Hap3	-:-	Non-Hap5
NJAU_W090	Hap3	-:-	Non-Hap5
NJAU_W095	Hap3	-:-	Non-Hap5
NJAU_W103	Hap3	-:-	Non-Hap5
Jack	Hap1	-:-	Non-Hap5
Dongnong50		-:-	Non-Hap5
Suinong14		-:-	Non-Hap5
Shidou25		TGA:TGA	Hap5
Shangdou151		TGA:TGA	Hap5
Ningdou6		TGA:TGA	Hap5
Ninghuang LD222		TGA:TGA	Hap5
Zhonghuang319		TGA:TGA	Hap5
Handou22		TGA:TGA	Hap5
Yudou2		-:-	Non-Hap5
Zheng1311		-:-	Non-Hap5
Zheng1307		-:-	Non-Hap5
Zheng92116		-:-	Non-Hap5
Zheng0163		-:-	Non-Hap5
Zhongdou62		-:-	Non-Hap5
Zhoudou38		-:-	Non-Hap5
Luodou6807		-:-	Non-Hap5
Chengdou10		-:-	Non-Hap5
Fudou18		-:-	Non-Hap5
Shidou31		-:-	Non-Hap5
Yongmindou9		-:-	Non-Hap5
Shanning44		-:-	Non-Hap5
Ningjingdou7		-:-	Non-Hap5
Ningdou7		-:-	Non-Hap5
Ninghuang135		-:-	Non-Hap5
Yandou11		-:-	Non-Hap5
Jindou100		-:-	Non-Hap5
Wandou40		-:-	Non-Hap5
Wandou60		-:-	Non-Hap5
Zhongdou63		-:-	Non-Hap5
Shengdou24		-:-	Non-Hap5
Huaidou17		-:-	Non-Hap5
Huaidou21		-:-	Non-Hap5
Handou24		-:-	Non-Hap5
Handou26		-:-	Non-Hap5

Supplemental Table S7. Previously identified QTL for LP-related traits on chromosome 19.

Trait	QTL	LOD	Flanking markers	Physical position	Additive	PVE (%)	References
PUE	qPUL	4.5	Satt652-Satt523	9,202,420	0.03	3.3	Zhang et al., 2009, Eutycia
SDW	qSL	4.4	Sat_408-Satt182	20,595,900	-0.14	5.4	
PARPEC	qPARPECL-07	4.03	Satt182-Satt652	9,202,420	0.089	16.4	Zhang et al., 2010, Plant Breeding
PARPEC	eqL6-1	4.56	Satt652-Sat_405	12,437,468	0.013	2.86	
	eqL6-3	5.36	Satt652-Sat_405	12,437,468	-0.298	2.6	
	eqL6-4	5.11	Satt652-Sat_405	12,437,468	-0.25	2.2	
	eqL6-5	3.55	Satt652-Sat_405	12,437,468	-0.104	1.05	
PARPEC|PARLP	eqL6-6	4.65	Satt652-Sat_405	12,437,468	-0.137	1.67	
HNAPA2008	Chr.19	4.8	AX-94187400	15,837,025	0.23	6.2	Zhang et al., 2014b, PLoS Genetics
HNPC2008	Chr.19	6.2	AX-94187410	15,939,989	0.54	7.86	
NJPC2008	Chr.19	4	AX-94187410	15,939,989	0.17	5.33	
RDW06-P	q19	2.76	Marker1014646	22,564,329	0.24	5.14	Zhang et al., 2016, Frontiers in Plant Science

PUE, phosphate use efficiency; SDW, shoot dry weight under low P (LP) condition; PARPEC, Pi efficiency coefficient (the ratio of pod number abscission rate at LP to normal P (NP)); PARLP, pod abscission rate under LP treatment; HNAPA2008, acid phosphatase activity traits obtained at Henan in 2008; HNPC, Pi concentration obtained at Henan in 2008; NJPC, Pi concentration obtained at Nanjing in 2008; RDW06-P, root dry weight obtained in 2006 under LP condition.

Supplemental Table S8. Primers used in this study.

**For RT-qPCR**	**Primers sequence**
GmGDPD2-F	TCTCGTTGCCGTGAAGATACTC
GmGDPD2-R	CGGTGGAGCCAAGAATCTCTG
GmMyb73-F	TGATTGACGACGACAACACTCC
GmMyb73-R	ACGCTGGACTCACTCACATCT
GmGA2ox1-F	ATCCAACAACACTTCAGGC
GmGA2ox1-R	TGTCTCACACTTCGGAACC
GmACP1-F	CCAGCGTGGGATTTGATATG
GmACP1-R	CAACAGAACTTGCTCCAACTC
GmACP2-F	GTCGTCGATGATTTGGGTTT
GmACP2-R	GCAAAACCTCTTCAATGTCC
GmPAP17-F	CGCTTACAACCAATCACAAG
GmPAP17-R	CGCTGTACCACTGCTTTTGC
LPR2-F	AAGTTGGAGATGTTCGTGG
LPR2-R	ACACTGTTGTTGGAGGCAG
RGI1-F	CCTTGTTGTGTGTGAACTCATC
RGI1-R	CAAACTTTCCATACTCCTCCC
EXPA7-F	ATCGAGCAGCAGGTTCATGT
EXPA7-R	TGCAAAAGTTCCAGCTATGTGA
OXS3-F	ACTTCTTTAGCAAGGGTGGA
OXS3-R	TCTGACTATTCAAGCCTCCC
**Promoter cloning primers**	**Primers sequence**
GmGDPD2-GUS1-F	ATTACGAATTCCCGGGGATCCGAGGAATGTGGTGTTTGGATG
GmGDPD2-GUS1-R	CAAGCTTGGCTGCAGGTCGACCGTGTGGTTGTGTAAATGCTT
GmGDPD2-GUS2-F	ATTACGAATTCCCGGGGATCCTATCCCAAGTCGATTGCTCG
GmGDPD2-GUS2-R	CAAGCTTGGCTGCAGGTCGACCGTGTGGTTGTGTAAATGCTT
**Subcellular localization**	**Primers sequence**
GmGDPD2-GFP-F	TTCCTCGTATTGAGTCTAGAATGCTGAATTTTCTTAATTT
GmGDPD2-GFP-R	GGATCCATGGTGAGCAAGGGTTCTTCTATTTCATTAATAA
GmMyb73-GFP-F	ACAAATCTATCTCTCTCGAGATGGACCGGATCAAAGGCCCAT
GmMyb73-GFP-R	GCTCACCATGGATCCCGAATCAACCCTGCTAATTC
GmGA2ox1-GFP-F	ACAAATCTATCTCTCTCGAGATGGTGTTGTTGTCCAAAGC
GmGA2ox1-GFP-R	GCTCACCATGGATCCCGAAGCTGCAATTCTTTCAA
**Overexpression**	**Primers sequence**
GmGDPD2-PTF101-F	AGAACACGGGGGACTCTAGAATGCTGAATTTTCTTAATTT
GmGDPD2-PTF101-R	GTCCATGGGGATCCACGCGTTTCTTCTATTTCATTAATAA
GmGA2ox1-PTF101-F	AGAACACGGGGGACTCTAGAATGGTGTTGTTGTCCAAAGC
GmGA2ox1-PTF101-R	AGAACACGGGGGACTCTAGACGAAGCTGCAATTCTTTCAA
GmMyb73-PTF101-F	AGAACACGGGGGACTCTAGAATGGACCGGATCAAAGGCCCAT
GmMyb73-PTF101-R	GTCCATGGGGATCCACGCGTCGAATCAACCCTGCTAATTC
**For RNAi and cas9**	**Primers sequence**
GmGDPD2-sg1-F	TGCAGCAGTACCTGTGAAGCACAA
GmGDPD2-sg1-R	AAACCCCTTGTGCTTCACAGGTACTGC
KO-GmGDPD2-F	GCAGCAAATCCAAATCTACGAC
KO-GmGDPD2-R	CGGTCTTCTCCTTTCGTCATATA
GmGA2ox1-sg1-F	TGGTCTCGTGCAGTAGGACCTCCCAATCCATTTGGGTTTTAGAGCTAGAAATAGC
GmGA2ox1-sg2-R	TGGTCTCGAAACGAGTCATTTCAGGACAAGCAGGGTGCACCAGCCGGGAATCGAA
KO-GmGA2ox1-F	GGCCGAGCAAACAACAAGAA
KO-GmGA2ox1-R	AGGCCTGAAGTGTTGTTGGA
GmMyb73-RI-F	GGGGACAAGTTTGTACAAAAAAGCAGGCTTCCAGGCTCCGCTGGTGCAACC
GmMyb73-RI-R	GGGGACCACTTTGTACAAGAAAGCTGGGTCGTGAAGTTGGAGGGTCATTG

**For Y1H**	**Primers sequence**
GmGDPD2-AbAi-1-F	GAATTCGAGCTCGGTACCCTCCACATGGACTTGAGTTT
GmGDPD2-AbAi-1-R	ACAGAGCACATGCCTCGAGTTGTTTCTGTAATTTACGTA
GmGDPD2-AbAi-2-F	GAATTCGAGCTCGGTACCATCCTACAACCACCACCTAA
GmGDPD2-AbAi-2-R	ACAGAGCACATGCCTCGAGCGTGTGGTTGTGTAAATGCTT
GmGDPD2-0800-F	GGTACCGGGCCCCCCCTCGAGGCGAAATTCCTCTTGTGCTGAG
GmGDPD2-0800-R	CGCTCTAGAACTAGTGGATCC GCGGTGGTGGTGTGATTCAA
**For Y2H**	**Primers sequence**
pBT3-SUC-GmGDPD2-F	AATATCTGCAATGGCCATTACGGCCATGCTGAATTTTCTTAATTT
pBT3-SUC-GmGDPD2-R	ATTCCTGCAGATGGCCGAGGCGGCTTCTTCTATTTCATTAATAA
**For BiFC assay**	**Primers sequence**
GmGDPD2-Cbifc-F	GCGCGCCACTAGTGGATCCAATGCTGAATTTTCTTAATTT
GmGDPD2-Cbifc-R	CTTTTGCTCCATCCCGGGTTCTTCTATTTCATTAATAA
GmGA2ox1-Nbifc-F	GCGCGCCACTAGTGGATCCAATGGTGTTGTTGTCCAAAGC
GmGA2ox1-Nbifc-R	AGCGGTACCCTCGAGGTCGACCGAAGCTGCAATTCTTTCAA
**For LUC assay**	**Primers sequence**
GmGDPD2-cLUC-F	CGGGGCGGTACCCGGGATCCAATGCTGAATTTTCTTAATTT
GmGDPD2-cLUC-R	AAAGCTCTGCAGGTCGACTTATTCTTCTATTTCATTAA
GmGA2ox1-nLUC-F	GACGAGCTCGGTACCCGGGATCCATGGTGTTGTTGTCCAAAGC
GmGA2ox1-nLUC-R	ACGCGTACGAGATCTGGTCGACCGAAGCTGCAATTCTTTCAA
**For pull down assay**	**Primers sequence**
GmGDPD2-His-F	AGCAAATGGGTCGCGGATCCAAAAACGGGTGCACGGATAA
GmGDPD2-His-R	TGTCGACGGAGCTCGAATTCTTCTTCTATTTCATTAATAA
GmGA2ox1-GST-F	TCCAGGGGCCCCTGGGATCCATGGTGTTGTTGTCCAAAGC
GmGA2ox1-GST-R	TCACGATGCGGCCGCTCGAGCGAAGCTGCAATTCTTTCAA
**For TA cloning**	**Primers sequence**
GmGDPD2-T-F	ATTTACACAACCACACGATGCTG
GmGDPD2-T-R	AAGCATCGCACCATTAGAGCAT
GmMyb73-T-F	ACGCTCGCTGACAAGTCCAA
GmMyb73-T-R	CTGTCTCGCTGTACATCAACCA
GmGA2ox1-T-F	ACAGAATAACGGCCGAGCAA
GmGA2ox1-T-R	TGCTTTACTGCAATTTGTGTCA
**For sequencing**	**Primers sequence**
35S	GACGCACAATCCCACTATCC
35S-Terminate	GCTCAACACATGAGCGAAAC
NOS	TTGCGGGACTCTAATCATAA
STU-TEST-3F	TGCTACCCTCATCCATCAGTC
STU-TEST-4R	TGTTGTGTGGAATTGTGAGCG
PGex6-p-GST-F	GGGCTGGCAAGCCACGTTTGGTG
PGex6-p-GST-R	CCGGGAGCTGCATGTGTCAGAGG
T7	TAATACGACTCACTATAGGG
T7 terminator	GCTAGTTATTGCTCAGCGG
pAbAi-F	GTTCCTTATATGTAGCTTTCGACA
pAbAi-R	CCATCTCGAAAAAGGGTTTGCC
CYC1pro-F	TACATTAGGACCTTTGCAGC
Cub-R	CAGCGTTCTACCGTCTTCT
PPR3-F	GTCGAAATTCAAGACAAGG
PPR3-R	AAGCGTGACATAACTAATTAC
nLUC-R	AATTGTTCCAGGAACCAGGGCGTA
cLUC-f	ACGAAGTACCGAAAGGTCTTACC
