# Supplementary material for: The Myb73–GDPD2–GA2ox1 transcriptional regulatory module confers phosphate deficiency tolerance in soybean
Source: Plant Cell. 2024 Feb 12;36(6):2176–200. doi: 10.1093/plcell/koae041 (PMC11132883; doi:10.1093/plcell/koae041)
Supplement: koae041_Supplementary_Data [file tpc.00539.2023-s05.pdf › tpc.00539.2023-s05.pdf]

THE PLANT CELL

A transcriptional regulatory module, Myb73-GDPD2-GA2ox1, confers phosphate deficiency tolerance in soybean

Dandan Hu, Ruifan Cui, Ke Wang, Yuming Yang, Ruiyang Wang, Hongqing Zhu, Mengshi He, Yukun Fan, Le Wang, Li Wang, Shanshan Chu, Jinyu Zhang, Shanshan Zhang, Yifei Yang, Xuhao Zhai, Haiyan Lv, Dandan Zhang, Jinshe Wang, Fanjiang Kong, Deyue Yu, Hengyou Zhang, and Dan Zhang

Review Timeline:

Submission Date:	15-Nov-2022
Editorial Decision:	24-Nov-2022
Resubmission Received:	13-Jun-2023
Editorial Decision:	25-Jul-2023
Revision Received:	24-Oct-2023
Editorial Decision:	21-Nov-2023
Revision Received:	03-Jan-2024
Accepted:	14-Jan-2024

1st Editorial Decision 24-Nov-2022

Dear Dr. Wang:

Thank you for submitting your manuscript entitled "A Myb73-GDPD2-GA2ox1 module confers phosphorus deficiency tolerance in soybean" to The Plant Cell. Your submission has been evaluated by members of the editorial board, and we regret to inform you that we are not recommending that your manuscript proceed further in the review process. Your submission was assessed at this stage by editorial board members who judged that the work would not be appropriate for The Plant Cell, and initiating additional review would only delay the eventual publication of your story. This decision reflects the priorities and platform of The Plant Cell, and is not meant to indicate that the manuscript is unsuitable for publication elsewhere.

To provide specific feedback, the editors judged that your manuscript does not represent a sufficient advance to warrant further consideration at TPC, for the following reasons:

1. Your mutants likely have growth effects independent of P status. T-tests only showed differences within a treatment. The appropriate statistical test to be employed to support your conclusions is an ANOVA.
2. It is unclear how your Y2H could have obtained an interaction with a membrane-localized protein.
3. Gene annotations - there is no evidence that GDPDP2 and the GA2OX truly have kinase or enzyme activities.
4. Your double mutant analysis does not contain the single knockouts as controls. Furthermore additivity suggests that they do not genetically interact.
5. The SNP analysis and LD decay to identify the interval does not specifically demonstrate that it is only GDPDP2 that is responsible for this trait. There is another gene within this interval; as well as potential long-range enhancers.

Therefore, we must decline to further consider this work and recommend submission to another journal.

We thank you for your interest in and support of The Plant Cell. We look forward to your next submission and hope we will be able to render a more positive decision on future work.

On behalf of the editorial board,

Blake Meyers, Editor-in-Chief

The Plant Cell

--- Transfer Options ---
You may wish to consider publication in Plant Direct. You can transfer your manuscript and associated submission data automatically to Plant Direct via the link below.

Please note that transfer to Plant Direct does not guarantee acceptance of your manuscript, as the editors of Plant Direct will still evaluate the manuscript. Should you choose to transfer your manuscript, you will receive an email request from Plant Direct asking you to log in to the submission site's Author Center, provide any missing information unique to Plant Direct's submission process, and complete the submission.

If you wish to transfer your paper to Plant Direct, then click the link below:
Link Not Available

Thank you for submitting your manuscript entitled "A Myb73-GDPD2-GA2ox1 module confers phosphorus deficiency tolerance in soybean" to The Plant Cell. Your submission has been evaluated by members of the editorial board, and we regret to inform you that we are not recommending that your manuscript proceed further in the review process. Your submission was assessed at this stage by editorial board members who judged that the work would not be appropriate for The Plant Cell, and initiating additional review would only delay the eventual publication of your story. This decision reflects the priorities and platform of The Plant Cell, and is not meant to indicate that the manuscript is unsuitable for publication elsewhere.

To provide specific feedback, the editors judged that your manuscript does not represent a sufficient advance to warrant further consideration at TPC, for the following reasons:

Dear editor, thank you very much for your suggestions. We have made a thorough revision on the manuscript, reperformed and added some experiments according to the constructive suggestions. Please see the following for detailed revisions and response to the questions.

1. Your mutants likely have growth effects independent of P status. T-tests only showed differences within a treatment. The appropriate statistical test to be employed to support your conclusions is an ANOVA.

Response: The QTL *qPE19* was first identified and be responsible for four low P related root traits (RRA, RRN, RRL, and RRV) based on a RIL population, and further verified by GWAS and be responsible for seven low P (LP) related traits (RRA, RRN, RRL, RRV, RRD, RPC, and RPAE) (**Fig. 1A-B**), proving that the QTL *qPE19* was a reliable low p related QTL. The gene GmGDPD2 (Glyma.19G069400) was proved to be the target gene (**Fig. 1E-G, S3A-B**). For the overexpression *GmGDPD2* (OE-*GmGDPD2*) lines, it showed higher root length, root area, PC in root, PAE in root, PC in shoot, and PAE in shoot than WT1 after both normal P (NP) and LP treatments, but the increasing proportion under LP condition (19.5%-92%) was much

higher than under NP condition (13.6%-77.2%) (**Fig. 2**). For the knockout *GmGDPD2* (KO-*GmGDPD2*) lines, it showed lower root length, root area, PC in root, PAE in root, PC in shoot, and PAE in shoot than WT1 after both normal P (NP) and LP treatments, but the decreasing proportion under LP condition (20.2%-83.5%) was much higher than under NP condition (16.3%-59. 9%). The transgenic *GmMyb73* and *GmGA2ox1* lines also showed higher increasing/decreasing proportion under LP condition than NP condition in most of the LP related root traits (**Fig. 3, 5**). Those results indicated that the mutants have growth effects depend on the P status, and *GmGDPD2*, *GmMyb73*, and *GmGA2ox1* were genes response to LP stress.

For the statistics comparison of phenotype among wild types and transgenic lines under normal P (NP) and low P (LP) treatment, the appropriate statistical test is ANOVA, in the previous version, parts of comparisons were determined by T-test, which only showed the difference between wild type and transgenic lines, but not reflected the difference between NP and LP treatment, so we recalculated the statistic based on one-way ANOVA, Duncan, the modified results were shown in **Fig. 1G-I, Fig. S6B**.

2. It is unclear how your Y2H could have obtained an interaction with a membrane-localized protein.

Response: The previous results of subcellular localization for GmGDPD2 and GmMyb73 was not rigorous, so we reperformed the experiments, and proved that GmGDPD2 localized on the cellular membrane (**Fig 1I**), GmMyb73 localized on the nucleus (**Fig 3C**), GmGA2ox1 localized on the nucleus and cellular membrane (**Fig. 4E**). We proved the interaction between GmGDPD2 and GmGA2ox1 through Y2H, BiFC, LUC and pull-down arrays (**Fig 4A-D**), and the Y2H and BiFC arrays were also reperformed to make it more rigorous. Y2H assays were performed using the DUAL membrane system (Dualsystems Biotech AG, Schlieren, and Switzerland) and conducted as described previously (Cui et al., 2022). Besides, we modified the method to make it more detailed.

3. Gene annotations - there is no evidence that GDPDP2 and the GA2OX truly have kinase or enzyme activities.

Response: Domain analysis based on SMART showed that the GmGDPD2 carrying a Pkinase Tyr domain (**Fig. S3C**), so we performed the in vitro autophosphorylation assays of GmGDPD2. His-tagged Pkinase_Tyr domain of GmGDPD2 (His-GmGDPD2-KD) was expressed in E. coli BL21 (DE3) and purified by affinity purification with Ni-NTA resin for kinase activity analysis. And the recombinant protein was confirmed by western blot using anti-His monoclonal antibody combined with Coomassie blue staining (**Fig. S3D**). Adding the Ca^{2+} chelator ethyleneglycoltetraacetic acid (EGTA) to the phosphorylation system can significantly reduce the upper band of GmGDPD2-KD, P-GmGDPD2-KD represents a phosphorylated form of GmGDPD2-KD, this result proved the GmGDPD2 with kinase autophosphorylation (**Fig. 1J**).

Besides, we performed the ELISA analysis of GA2 dioxygenase activity for the WT1 and two KO-*GmGDPD2/GmGA2ox1* lines that grown in NP and LP condition. The activity of GA2 dioxygenase in KO-*GmGDPD2/GmGA2ox1* roots was significantly reduced under both NP and LP conditions (p=3.10 × 10^{-3} - 4.86 × 10^{-3}) **(Fig. 6L)**

4. Your double mutant analysis does not contain the single knockouts as controls. Furthermore additivity suggests that they do not genetically interact.

Response: We first proved the interaction between GmGDPD2 and GmGA2ox1 through Y2H, BiFC, LUC and pull-down arrays (**Fig 4A-D**). *GmGA2ox1* showed a moderate expression level in roots relative to other selected tissues (**Fig. S6A**), and its expression was significantly increased in OE-*GmGDPD2* roots and significantly decreased in KO-*GmGDPD2* roots compared with WT1 under both NP and LP conditions (**Fig. S6B**), these results indicating that *GmGA2ox1* plays a key role in roots, and its expression level is likely affected by *GmGDPD2*. For KO-*GmGA2ox1* lines, they showed lower root length, root area, number of root tips, root volume, PC in root, PAE of root than WT in both NP and LP condition, especially in LP condition,

but the decrease were not significant except PAE of root. While, the KO-*GmGDPD2*/*GmGA2ox1* lines showed significant lower root length, root area, number of root tips, root volume, and PAE of root under both NP and LP condition. Although this phenotypic evaluation didn't contain the single knockouts of KO-*GmGDPD2*, these results also proved to some extent that GmGDPD2 genetically interacted with GmGA2ox1.

5. The SNP analysis and LD decay to identify the interval does not specifically demonstrate that it is only GDPDP2 that is responsible for this trait. There is another gene within this interval; as well as potential long-range enhancers.

Response: For the QTL *qPE19-2* identified by GWAS, SNP AX-94187201 (chr19: 15,989,213, based on Williams82 a4 genome) was one of the top SNPs of *qPE19-2* (**Table S3**) that was significant in six LP-related traits, its two haplotypes (G/T) in the 367 soybean diverse accessions indicated that accessions carrying allele T were, on average, 63% - 180% higher in the seven LP-related traits than those carrying allele G ($p = 4.7 \times 10^{-8} - 7.8 \times 10^{-26}$) (**Fig. 1D**), indicating that AX-94187201 is highly associated with *qPE19-2* in controlling these traits. We scanned a 700 kb interval centered on the SNP AX-94187201, which was a relatively larger region according to the estimated LD decay rate of approximately 200kb in a soybean natural population (Wen et al., 2015) and only identified two predicted genes (*Glyma.19G069400*, *Glyma.19G069500*) within the interval (**Fig. 1E**). Interestingly, SNP AX-94187201 (S1308) is located within the coding sequence (CDS) of *Glyma.19G069400* (**Fig. 1H**). Besides, RT-PCR experiment demonstrates that *Glyma.19G069400* was expressed in all tissues (root, stem, leaf, flower, pod, and seed) of W82 with predominant expression in roots (**Fig. 1F, S3A**), whereas, *Glyma.19G069500* was nearly not expressed in any of the tissues based on the online database (**Fig. S3A**). Further expression examination demonstrated that *Glyma.19G069400* was highly induced in roots after LP treatment for 3d (**Fig. 1G**). These results strongly indicated that *Glyma.19G069400* is the most promising candidate gene of *qPE19-2*.

Prof. Dan Zhang
Henan Agricultural University
College of Agronomy
Zhengzhou
China

Dear Dr. Zhang:

We have received reviews of your manuscript entitled "A Myb73-GDPD2-GA2ox1 module confers phosphorus deficiency tolerance in soybean." Thank you for submitting your best work to The Plant Cell. The editorial board agrees that the work you describe is substantive, falls within the scope of the journal, and may become acceptable for publication, pending revision and potential re-review.

We ask you to pay attention to the following points in preparing your revision:

All agreed that the manuscript provides novel and findings of significant importance to the field. In preparing your revision, please pay particular attention to reviewers 1 and 3 who state that important methodological information is missing, particularly with respect to the quantitative genetic analysis (ie population structure, models, genome sequence/SNPs etc; replicates, measurement of root traits). All reviewers also stated that more expression data is necessary to support the findings in the title.

However I should stress that we are reluctant to see manuscripts undergoing multiple rounds of revision and would be unlikely to offer you more than one chance to satisfy the reviewers.

In addition, we find that the writing suffers from some problems with grammar and awkward phrasing, and in general lacks clarity and conciseness. We strongly encourage the use of a professional editing service to ensure that the work is presented in the best manner and reaches the broadest possible audience. Several editing services have agreed to extend a discount to authors coming to their website via this ASPB page https://aspb.org/aspb-journal-submission-editing-services/.

Please contact us if there are ambiguous comments or if you wish to discuss the revision.

Given the nature of the comments, we are offering you 60 days from when we have issued this decision to complete the revision. If a revision is not returned within this time frame, and if you have not been granted an extension, we will withdraw the manuscript, which will leave you free to submit the work elsewhere. If you need an extension, we encourage you to contact us at any point before the 60 days have passed.

When you are ready to submit the revised version, please upload a highlighted copy that indicates all changes made in response to the editor and reviewer recommendations. Include an itemized list of all changes made in response to each of the reviewer's suggestions in the "Response to Reviewers" section; please note that reviewers do not have access to your cover letter, nor was this decision letter shared with them.

Thank you for the privilege of reviewing your work. We look forward to receiving your revised manuscript.

On behalf of the editorial board,

Reviewing Editor, Board of Reviewing Editors
Siobhán Brady, Senior Editor
Blake Meyers, Editor-in-Chief

The Plant Cell

Please note the following:

-The Plant Cell now requires authors to complete and submit an author revisions checklist upon submission of a revised manuscript. The aim of the checklist is to aid authors in preparing a high-quality manuscript, facilitate the review and assessment of revised manuscripts, and help to ensure that journal standards are maintained across the board. If your manuscript is accepted, the completed checklist will be published as supplemental material attached to the article online. Please download a copy of the checklist (pdf fillable form) at this link, for submission with your revised manuscript: https://tpc.msubmit.net/html/Author_Revisions_Checklist.pdf.

-Supplemental materials should be restricted to large datasets and tables, presentation of replicates, and validation of

reagents, methods, or genotypes. Any data that are used to support the major claims must be in the main manuscript. Supplemental figure legends must indicate what figure in the main manuscript is supported by the supplemental data presented. Please justify how each of the supplemental figures meet the criteria.

-Sampling methods and nature of "biological replicates" should be described precisely (i.e. different plants, parts of plants, pooled tissue, independent pools of tissue, sampled at different times, etc), along with a clear description of and rationale for any statistical analyses conducted. The reader should know exactly what was sampled; what forms the basis of the calculation of any means and statistical parameters reported. This is also necessary to ensure that proper statistical analysis was conducted.

-Want to add this revision deadline to your calendar? Click below!

-- Reviewer comments:

Reviewer #1 (Comments for the Author):

Improving low phosphorus (P) tolerance in crops is a major topic. In this work, the authors reported the identification of A Myb73-GDPD2-GA2ox1 module that confers phosphorus deficiency tolerance in soybean. The manuscript is nicely written and experiments are well performed. Nevertheless, the manuscript needs improvement in many sections, including the discussion where a role for auxin in root growth under LP was identified earlier doi: 10.1093/plphys/kiab441 through GWAS approach in Arabidopsis.

the information in the introduction is a bit vague, and some statements are missing references (i.e. lines 61-64), and more relevant are missing support statements like the ones on lines 54-57. More importantly, reading the abstract and the introduction, we do not understand what are the LP-related traits, total root length, and root biomass,.... which then can be identified in the first section of results. But this is a bit late, and worth mentioning in the introduction, with a brief mention of why these traits are the most important ones regarding Pi deficiency. the absence of compelling elements supporting this research reduces the interest of the readers to pursue reading. Therefore, I suggest rewriting the introduction and improving the reference citations to give credit to the initial works.

The methods for QTLs detection should be further developed, as it is enough to referer to the initial paper. The variation in the population used should be better described. Again, referring to the previous paper for such an important part of this work is not helpful. The same applies to GWA studies.

The authors presented a nice phenotype for plants growing on NP and LP in Figure 2F. However, this difference is absent in subsequent figures such as Figures 3F and 6A. Is the age of the plant used changed, or have growth conditions? Please indicate this information in the Figure legends. Indeed, the figure legends are missing important information to understand the figures (number of plants, age of plants, treatments, statistical tests).

The authors considered the following 4 traits: RRA (relative root surface area), RRL (relative root length), RRN (relative number of root tips) and RRV (relative root volume) under LP or normal P (NP) conditions. Interestingly, the authors find some known QTLs and list a new QTL qPE19-1 that is located on chromosome 19 and detected for all four LP-related root traits with two environments. At this stage this statement is confusing as if this QTL is found in two environments means to me that this QTL is not dependent on P availability! If this was what the result to present please be precise and provide the % that explains the variation of each trait.

Here again, are the GWAS peaks unique to LP conditions? The standard of GWAS analysis is the mixed model. Why the authors used the Linear Model for the Analysis of variance (ANOVA) of phenotypic data? Have the authors confirmed that the population structure is suitable for GWAS for these traits? Please provide all data supporting the GWAS analysis to be able used by the authors. Are the genomic sequences for this population publically available? Please provide links. Finally, the chromosome distance used to identify candidate genes is arbitrary, or more based on LD. Please provide more information in the mat and met section as more needed information is either missing or referred to a published paper (often one single paper, and therefore needs more validation).

The authors reported the identification of A Myb73-GDPD2-GA2ox1 module that confers phosphorus deficiency tolerance in soybean. The GmGDPD2 is proposed to regulate LP tolerance likely through influencing root auxin and GA levels-associated cell division in the root meristem zone. A role for auxin transport to regulated root growth under LP was identified earlier doi: 10.1093/plphys/kiab441 this reference is absent and not discussed despite the tight mechanism found in this work.

The authors performed a nice protein and microscopy work to identify the protein that performs the module regulating the 4 traits related to LP in soybean. Nevertheless, at this stage of the manuscript, something is missing. For example,

It is still unclear how the change in expression of these proteins relates to P content, P concentration, and P uptake capacity, and this affects the aboveground phenotypes. Is better root growth translate to a better P uptake? These types of data are missing to better appreciate how the discovered mechanism contributes to better plant growth in LP conditions.

Is the Figure 8 show "Lateral roots" or "root tip"?

Reviewer #2 (Comments for the Author):

The manuscript by Hu et al. identified and characterized a Myb73-GDPD2-GA2ox1 module which demonstrates phosphorus deficiency tolerance in soybean. Linkage mapping and GWAS identifies, a major QTL 'qPE19' controlling low P associated root traits. GmGDPD2 gene was claimed as underlying gene for qPE19 QTL, while the haplotype analysis determines that 'Hap5' contains the allele for LP tolerance. They overexpressed Hap5-type GmGDPD2 which shows better root architecture & P efficiency while knockout (through CRISPR/Cas9) plants show opposite effects. Authors demonstrates GDPD2 was negatively regulated by Myb73 and double mutant for GmGDPD2 and GmGA2ox1 shows GmGDPD2 controls low P tolerance. This is an interesting work and most of the experimental analysis is detailed studied, except the identification of candidate gene is not precise enough and needs further refinement. My concerns are listed below:
Authors performed SNP analysis and LD decay to narrow down the region and identified GDPDP2 as candidate but only these analyses aren't sufficient to claim only GDPDP2 is specifically associated for P deficiency tolerance, what about other genes in this interval?
What are the key putative motif(s) in GmGDPDP2 which are responsible for P tolerance, please investigate and describe in the manuscript. Author needs to add multiple sequence alignment of GmGDPDP2 proteins from tolerant and sensitive lines and highlight the (putative) key motifs for stress tolerance.
It's unclear why authors didn't show expression of GDPDP2 gene under native promoter. Haplotype analysis shows unique haplotypes in promoter for Hap5.
It was claimed that, Hap5 shows significant higher root relative area as compared to other haps, did author tested the SNPs underlying this Hap? authors are encouraged to develop appropriate markers to discriminate P tolerance and sensitive accessions which can confirm the functional role of GDPDP2 in breeding program.
GmGA2ox1 localized on the both nucleus and cellular membrane, did author tried other stain like DAPI to confirm the nuclear localization?
Merged figure of subcellular localization in case of Fig. 1I and 4E looks similar.
Put the appropriate scale bars for root traits images throughout the manuscript.
In fig. 1I, 3C - what does the scale bar indicates?

Reviewer #3 (Comments for the Author):

The manuscript identifies a genomic loci associated with several seedling root traits under low phosphate availability in soybean, and progresses this to identify the underlying gene, SNP and functional implications of these in terms of plant responses to low phosphate availability.

Major comments.
1. The Introduction and Discussion sections are very selective on the literature they cite, and do not acknowledge many papers in this area that have direct relevance to their findings either in soybean or other plant species. This should be addressed in any resubmission.

2. Methodologically the manuscript is mostly sound. However, as is customary when evaluating OE and KO transgenic lines, data from multiple independent OE or KO lines are presented to demonstrate consistency between transformation events, which is lacking from this manuscript.
3. There is also a lack of detail on how the phenotypic traits were measured, the levels of replication used and how they were analysed.
3.1 For example, why were the plants grown over two years? Where all the lines grown at once, or were they spread over the two years, or all lines grown in both years? How were the experiment-to-experiment variations accounted for in the analyses? How many replicates per line per treatment per year were utilized?

3.2 Root traits are notoriously variable. How were the root traits quantified - no details are given. For the root trait measurements, no indication of replication level is provided. This is critical, given the highly variable nature of root traits.
3.3 The ANOVA used for phenotypic data only includes genotype and year-location as fixed factors - was treatment not included as a factor in this analysis?

4. Figure 8 contains some speculative elements - Organic acids were not quantified, so they should not be present. What is meant by the 'Root-specific Growth factor'? Not all the genes to the left of the root tip were quantified in the manuscript, so these should be refined and only mention those quantified. There are also no data presented on P reutilisation being driven by GmGDPD2, so this should be removed.

5. The use of the term 'Myb73-GDPD2-GA2ox1 module' in the title implies these genes are linked in some way, such as an operon or all physically interact as a tri-mer. However, the evidence to demonstrate the physical interaction of all three at the same time is not presented, so this should be revised.
6. Overall, the manuscript would benefit from editing for English language.

Minor comments.
L33 - replace 'controls' with 'associated with'
L59 - plants actively take up phosphorus, so replace 'absorb' with 'taken up'
General point - plants take up phosphate not phosphorus and fertilizers are always phosphate based. So careful use of the abbreviation of phosphorus to P is needed when referring to the uptake of phosphate and phosphate based fertilizers.
L61-64 - costs of fertilizers are country specific, so this needs to be acknowledged and a reference for these data provided.
L80-84 - one of the most significant impacts of phosphate deficiency on soybean productivity is the restriction it places on the development and functioning of the nitrogen fixing symbiosis with rhizobium - this should be acknowledged here.
L104 - the authors cite one study utilizing GWAS for identifying adaptations to low phosphate availability, but there are many others that should be acknowledged here - Wang et al 2022a,b, Du et al 2020, Yang et al 2020, Lu et a; 2018, plus probably others I didn't find with a simple search.
L108 - replace 'controls' with 'associated with'
L158 - replace 'controlling' with 'associated with'
L172 - replace 'after' with 'in'
L183 - Arabidopsis GDPDL2 has previously been shown to be responsive to low phosphate availability - this should be acknowledged - see Cheng et al., (2011) The Plant Journal (2011) 66, 781-795.
L213 - replace 'absorption' with 'up take' - absorption implies a passive process.
L254 - mentioned 'in the field' but no field trials are described - do you mean in the growth chamber?
L282 - revise to read 'GmGDPD2 may be negatively regulated in planta by GmMyb73'
L326 - where you say 'transgenic plant' do you mean OE lines or both OE and KO lines?
L341 - Revise to read 'Given the potential for physical in planta interaction between...'
L345 - replace 'severer' with 'greater'.
L353 - several papers have show a role for GA in plant adaptations to low phosphate availabilities, so to say this is 'rare' is incorrect.
L367 - GAs
L372 and 375 - delete 'greatly'
L381 and 382 - delete 'much'
L410 - refers to root hairs, but these were not quantified, so this is speculative and should be removed.
L418 - is this a new pathway? - both the GDPD2 (Cheng et al., 2011) and GA2ox1 (Zhang et al., (2019) Plant Sci 283, 177-188) have been identified in previous studies as associated with tolerance to low P availability in other plants.
L462 - likely downstream or regulated by an independent pathway?

Reviewing Editor comments:

Summary of changes to figures and tables

Figure/table	Description
**Fig. 1I, 3C, 4E**	the three figures were once again conducted independently<br>- as requested by reviewer 2
**Fig. 2A**	new figure showing protein expression of GmGDPD2<br>- as requested by reviewer 1
**Fig. 4F, S18**	new figure showing exogenous hormones restore root phenotype<br>- as requested by reviewer 2
**Fig. 5H-I, S22**	new figure showing the changes of Pi-uptake rate and organic acid content<br>- as requested by reviewer 1
**Fig. S2H**	new figure showing LD decay of 367 soybean accessions<br>- as requested by reviewers 1 and 2
**Fig. S4A**	new figure showing expression pattern of *Glyma.19G069500*<br>- as requested by reviewers 1 and 2
**Fig. S8, S9**	new figure showing *GmGDPD2* gene/amino acid sequence of Hap1 and Hap5<br>- as requested by reviewers 2
**Fig. S10**	new figure showing predicted GmGDPD2 structure of Hap1 and Hap5<br>- as requested by reviewers 2
**Fig. S12, S13**	new figure showing phenotype of each transgenic line<br>- as requested by reviewer 3
**Fig. S14**	new figure showing secretion of organic acids by roots<br>- as requested by reviewer 3
**Fig. S16H-K**	new data added showing the expression of three *DELLA* genes and *GmPT7*<br>- as requested by reviewers 2 and 3
**Fig. S17B**	new figure showing *GmGA2ox1* expression data of all the three lines<br>- as requested by reviewer 3
**Fig. S20**	supplementary data showing root traits of all the three transgenic lines<br>- as requested by reviewer 3
**Fig. S21**	new figure showing phenotype changes of double mutant
**Fig. S23-24**	new phytohormones data as the complement for **Fig. 5J-N**<br>- as requested by reviewer 3
**Fig. S25**	details of the phenotype are complementary to **Fig. 6B-I**<br>- as requested by reviewer 3
**Table S1, S3**	new tables showing descriptive statistical results for RIL/natural population<br>- as requested by reviewer 1
**Table S6**	new table showing KSAP marker of *GmGDPD2* Hap5 in 79 soybean lines<br>- as requested by reviewer 2

Reviewer #1 (Comments for the Author):

Improving low phosphorus (P) tolerance in crops is a major topic. In this work, the authors reported the identification of A Myb73-GDPD2-GA2ox1 module that confers phosphorus deficiency tolerance in soybean. The manuscript is nicely written and experiments are well performed.

Response: Thank you so much for the encouragement and your constructive comments. We tried our best to revise the manuscript, and we hope it will address all your questions. Thank you again.

Nevertheless, the manuscript needs improvement in many sections, including the discussion where a role for auxin in root growth under LP was identified earlier doi: 10.1093/plphys/kiab441 through GWAS approach in Arabidopsis. The information in the introduction is a bit vague, and some statements are missing references (i.e. lines 61-64), and more relevant are missing support statements like the ones on lines 54-57.

Response: We thank the reviewer for raising this concern and agree that there are some improvements needed in the Introduction and Discussion sections. We reviewed a number of recent relevant studies and summarized them in the corresponding sections. A detailed description of this point has been added to the text. For example, in the Introduction section, described as "*Plants have evolved a series of adaptive responses, including modified root architecture and hormone regulatory effects, genetic changes, and domestic adaptation, to increase Pi acquisition ……Another auxin gene, PIN-LIKES7 (PILS7), an auxin efflux carrier,…*" (Lines 67-145).

In the Discussion section, described as "*In Arabidopsis, Cheng et al. (2011) characterized GDPD family genes and revealed that the expression of most members could be induced by Pi starvation……Changes in auxin gradients then impact the formation of lateral roots and root hairs, thereby directly or …*" (Lines 520-548).

More importantly, reading the abstract and the introduction, we do not understand what are the LP-related traits, total root length, and root biomass,.... which then can be identified in the first section of results. But this is a bit late, and worth mentioning in the introduction, with a brief mention of why these traits are the most important ones regarding Pi deficiency. the absence of compelling elements supporting this research reduces the interest of the readers to pursue reading. Therefore, I suggest rewriting the introduction and improving the reference citations to give credit to the initial works.

Response: Thank you for pointing this out. Following the suggestion, we added a summary of several representative papers in the Introduction section to acknowledge recent progress and related achievements. We also clearly indicated LP-related traits

by summarizing them in the Introduction followed by references and brief descriptions. A summary on this point has been added to the text as follows: "*Plants have evolved a series of adaptive responses, including modified root architecture and hormone regulatory effects, genetic changes, and domestic adaptation,…… The changes in these morphological and physiological traits have been used as representative traits to evaluate...*" (Lines 67-82). Thank you very much again.

The methods for QTLs detection should be further developed, as it is enough to referer to the initial paper. The variation in the population used should be better described. Again, referring to the previous paper for such an important part of this work is not helpful. The same applies to GWA studies.

Response: Thank you for this suggestion. We added more details to the method for GWAS and linkage mapping in the Material and Method section, including the phenotyping methodology, models, genotyping procedure, and QTL identification method. We also cited the initial papers to support the methods. In addition, we added the descriptive statistical results of the RIL and GWAS populations to **Table S1** and **Table S3**, respectively. Detailed description of the GWAS and RIL population, described as "*A total of 367 soybean accessions (including 105 wild soybeans and 262 cultivated accessions) derived from 26 provinces within six ecological regions (North (I), Huang-Huai-Hai…*" (Lines 605-614).

For phenotyping methodology, described as "*Hydroponics experiments were conducted in 2018 and 2019 to assess the traits related to Pi efficiency as previously described (Li et al., 2016). Each hydroponics experiment was independently replicated at least three times.…*". (Lines 615-633)

For the genotyping procedure, "*The population of 367 soybean accessions was genotyped using the NJAU 355K SoySNP array as previously described, the structure analysis showed that they were partitioned into five groups and were suitable for GWAS (Wang et al., 2016). …*". (Lines 693-700, 720-722)

For the QTL identification method, described as "*The QTL analysis was subsequently performed using the inclusive composite interval mapping (ICIM) method to identify additive QTLs (Meng et al. 2015). The P values for …*". (Lines 723-729)

The authors presented a nice phenotype for plants growing on NP and LP in Figure 2F. However, this difference is absent in subsequent figures such as Figures 3F and 6A. Is the age of the plant used changed, or have growth conditions? Please indicate this information in the Figure legends. Indeed, the figure legends are missing important information to understand the figures (number of plants, age of plants, treatments, statistical tests).

Response: Thank you for your professional insight. **Fig. 2F** (**Fig. 2G,** in the revised manuscript) shows the phenotypic changes in the agronomic traits of *GmGDPD2* stable transgenic plants after 45 days under low P stress in the pot experiment. Considering that low-P stress has a great effect on the flowering and pod development of soybean, the purpose of **Fig.** 2F (**Fig. 2G,** in the revised manuscript) is to identify the phenotypic changes in the agronomic traits of transgenic plants under different P treatments at the flowering and pod stages. Additionally, because the effect of low P stress on soybean growth and development and phenotypes will be aggravated with the extension of stress time, the phenotypic changes in **Fig. 2F** (**Fig. 2G,** in the revised manuscript) will be more obvious. **Fig. 3F** and **Fig. 5A** are mainly intended to show the phenotypic changes in root-related traits of *GmMyb73* and *GmGA2ox* transgenic plants after 10 days under different P treatments in the hydroponics experiment. We evaluated 3 plants for each line and each treatment, and the statistical test was calculated using SPSS Statistics 17.0 (SPSS, Inc., Chicago, IL, USA) (one-way ANOVA, $P < 0.05$). Detailed information on the number of plants, age of plants, treatments, and statistical tests was added to the figure legend and highlighted in red (**Figs. 2, 3, 5**).

The authors considered the following 4 traits: RRA (relative root surface area), RRL (relative root length), RRN (relative number of root tips) and RRV (relative root volume) under LP or normal P (NP) conditions. Interestingly, the authors find some known QTLs and list a new QTL qPE19-1 that is located on chromosome 19 and detected for all four LP-related root traits with two environments. At this stage this statement is confusing as if this QTL is found in two environments means to me that this QTL is not dependent on P availability! If this was what the result to present please be precise and provide the % that explains the variation of each trait.

Response: Thank you for pointing this out. This confusion might mainly come from the unclear statement in the main text. The two environments refer to two independent repeated experiments conducted in two different years, 2018 and 2019, rather than different P treatments. Specifically, the root and PAE-associated phenotypes for both the RIL population and association panel were measured in 2018 and 2019, and the ratio for each trait in LP relative to NP was used as an LP-related trait. Taking the RRL (relative root length) trait as an example, we evaluated the root length of the population under the NP treatment and LP treatment in the same environment at the same time. Then, we calculated the relative root length through the following formula:

$$\text{RRL (relative RL)} = \frac{\text{RL under LP treatment}}{\text{RL under NP treatment}}$$

We modified the description of "two environments" to make it clear, as following "*To well reflect the LP tolerance response, we used the ratio of measured values for each*

trait for one line under LP relative to NP as an LP-related trait, for example, RRL (relative RL) = RL under LP treatment/RL under NP treatment.". (Lines 651-654).

Here again, are the GWAS peaks unique to LP conditions? The standard of GWAS analysis is the mixed model. Why the authors used the Linear Model for the Analysis of variance (ANOVA) of phenotypic data? Have the authors confirmed that the population structure is suitable for GWAS for these traits? Please provide all data supporting the GWAS analysis to be able used by the authors. Are the genomic sequences for this population publically available? Please provide links. Finally, the chromosome distance used to identify candidate genes is arbitrary, or more based on LD. Please provide more information in the mat and met section as more needed information is either missing or referred to a published paper (often one single paper, and therefore needs more validation).

Response: Thank you for raising this concern. The GWAS peaks were identified based on the ratio in LP relative to NP treatment in the same environment at the same time, so the peak associations were significantly associated with the change for a given trait under LP compared with NP, not under a certain P condition. Taking the relative root length (RRL) trait as an example, the GWAS peaks for the RRL trait are significant for the changes in root length (RL) in LP relative to NP conditions. To make it clear, we made changes in the corresponding parts, as follows: "*To well reflect the LP tolerance response, we used the ratio of measured values for each trait for one line under LP relative to NP as an LP-related trait, for example, RRL (relative RL) = RL under LP treatment/RL under NP treatment.*". (Lines 651-654).

In addition, we also corrected the description of the ANOVA model in the text and added it to the text read as "*We performed analysis of variance (ANOVA) on phenotypic data using the mixed linear model procedure in SPSS Statistics 17.0 (SPSS, Inc., Chicago, IL, USA)...*" (Lines 681-691)

The population structure and relatedness among the 367 diverse soybean accessions were described in Wang et al., 2016 and the genomic sequences are publicly available (https://doi.org/10.1038/srep20728). The panel was divided into five groups and has been used in GWAS (Wang et al. 2016), proving that it is suitable for GWAS. In the revised manuscript, we reanalyze the physical position of these SNPs based on the up-to-date genomic information of Wm82.a4 (**Table S9**). We analyzed the linkage disequilibrium (LD) for the 367 soybean accessions, and the distance over which LD decayed to 0.2 was 226.5 kb (**Fig. S2H,** newly added), which is consistent with the LD distance (0.2) of 200 kb for a different association panel comprising 302 diverse soybeans (Zhou et al., 2015, doi:10.1038/nbt.3096). So we scanned a 250 kb on each side of the lead SNP AX-94187201, this 500 kb region contains only two predicted

genes, *Glyma.19G069400* and *Glyma.19G069500* (**Fig. 1E**). *Glyma.19G069400* was mainly expressed in roots (**Fig. 1F, S4C**) and was highly induced after LP treatment (**Fig. 1G, S5**), while *Glyma.19G069500* was rarely expressed in all tissues according to qRT-PCR experiments (**Fig. S4A**, newly added experiments) and public transcriptome database (**Fig. S4B**), and qRT-PCR experiments showed that it was not expressed after LP treatment. In addition, *Glyma.19G069400,* the lead SNP, was located within the coding sequence (**Fig. 1H**). Detailed description on these points has been added to the text (Lines 203-222, 693-695, 699-700).

The authors reported the identification of A Myb73-GDPD2-GA2ox1 module that confers phosphorus deficiency tolerance in soybean. The GmGDPD2 is proposed to regulate LP tolerance likely through influencing root auxin and GA levels-associated cell division in the root meristem zone. A role for auxin transport to regulated root growth under LP was identified earlier doi: 10.1093/plphys/kiab441 this reference is absent and not discussed despite the tight mechanism found in this work.

Response: Thank you so much for this suggestion. We added this reference to the text accordingly. In the Introduction section, described as "*Another auxin gene, PIN-LIKES7 (PILS7), an auxin efflux carrier, was identified by genome-wide association study (GWAS) of Pi concentration in Arabidopsis plants (Yi et al., 2021)....*" (Lines 84-86). In the Discussion section, described as "*Changes in auxin gradients then impact the formation of lateral roots and root hairs, thereby directly or indirectly influencing Pi uptake and/or Pi translocation (Yi et al., 2021). ...*" (Lines 538-540).

The authors performed a nice protein and microscopy work to identify the protein that performs the module regulating the 4 traits related to LP in soybean. Nevertheless, at this stage of the manuscript, something is missing. For example, It is still unclear how the change in expression of these proteins relates to P content, P concentration, and P uptake capacity, and this affects the aboveground phenotypes. Is better root growth translate to a better P uptake? These types of data are missing to better appreciate how the discovered mechanism contributes to better plant growth in LP conditions.

Response: This is a great suggestion. We appreciate it. We setup the experiment as soon as we received the review comments and obtained the GmGDPD2 protein expression results after many attempts. For GDPD2 protein, its expression levels were much higher in LP-treated OE-GmGDPD2 roots than those in NP grown OE-GmGDPD2 (**Fig. 2A, new Figure**), suggesting that GmGDPD2 protein is also inducible by LP and positively correlated with the observed increase in PC, P uptake and likely the yield related traits. Unfortunately, we are unable to obtain the expression result for Myb73 and GA2ox1 proteins at this moment.

Moreover, we newly tested the Pi uptake rate of all *GmGDPD2* and *GmGA2ox1* transgenic lines and two wild types. The results showed that all the lines pretreated in LP conditions showed a higher Pi uptake rate than the lines pretreated in NP conditions (**Fig. 5H-I, S22, new Figure**). (Lines 435-445)

Soybean roots showed longer root length, larger root area and volume, more root tips in LP condition than NP condition, and the overexpression of *GmGDPD2* and *GmGA2ox1* showed longer root length, larger root area and volume, more root tips, higher PC in root and PAE in root when compared with wild types. These results strongly suggest that better root growth and production contribute greatly to Pi uptake.

Is Figure 8 show "Lateral roots" or "root tip"?

Response: Thanks, it is Figure 7 in the revised manuscript, and it was corrected.

Reviewer #2 (Comments for the Author):

The manuscript by Hu et al. identified and characterized a Myb73-GDPD2-GA2ox1 module which demonstrates phosphorus deficiency tolerance in soybean. Linkage mapping and GWAS identifies, a major QTL 'qPE19' controlling low P associated root traits. GmGDPD2 gene was claimed as underlying gene for qPE19 QTL, while the haplotype analysis determines that 'Hap5' contains the allele for LP tolerance. They overexpressed Hap5-type GmGDPD2 which shows better root architecture & P efficiency while knockout (through CRISPR/Cas9) plants show opposite effects. Authors demonstrates GDPD2 was negatively regulated by Myb73 and double mutant for GmGDPD2 and GmGA2ox1 shows GmGDPD2 controls low P tolerance. This is an interesting work and most of the experimental analysis is detailed studied, except the identification of candidate gene is not precise enough and needs further refinement. My concerns are listed below:

Response: Thank you for your encouragement and helpful suggestions.

Authors performed SNP analysis and LD decay to narrow down the region and identified GDPDP2 as candidate but only these analyses aren't sufficient to claim only GDPDP2 is specifically associated for P deficiency tolerance, what about other genes in this interval?

Response: Thank you for this helpful suggestion. Following the suggestion, we analyzed the linkage disequilibrium (LD) for the 367 soybean accessions, and the distance over which LD decayed to 0.2 was 226.5 kb (**Fig. S2H,** newly added). So we scanned a 250 kb on each side of the lead SNP AX-94187201, this 500 kb region contains only two predicted genes, *Glyma.19G069400* and *Glyma.19G069500* (**Fig.**

1E). All the gene examination analyses including the tissue specific expression pattern analysis, LP induced expression analysis, sequence variaiton-LP related traits association (**Fig.1**) , and liteature investigation pointed to that *Glyma.19G069400* is the most promising candidate genes for the QTL, and our later detailed function validation in soybean further confirm that *Glyma.19G069400* underlies the QTL. The parallel analysis for *Glyma.19G069500* didn't support that it has any relationship with the QTL or LP-related traits, although we haven't test its funcition in soybean. An additional description on this point has also been added to the text as "*Considering that the LD (decay to 0.2) for our 367 soybean accessions was 226.51 kb (Fig. S2H), we scanned a 500 kb interval centered around the SNP AX-94187201, and identified two predicted genes (Glyma.19G069400, Glyma.19G069500)…*" (Lines 203-222, 693-695, 699-700).

What is the key putative motif(s) in GmGDPDP2 which are responsible for P tolerance, please investigate and describe in the manuscript? Author needs to add multiple sequence alignment of GmGDPDP2 proteins from tolerant and sensitive lines and highlight the (putative) key motifs for stress tolerance.

Response: Thank you for this helpful suggestion. The haplotype analysis results indicated that the 3 bp deletion in exon 1 account for Hap 5's higher tolerance than the other four Haps. In the revised manuscript, we re-sequenced the *GmGDPD2* gene in six soybean accessions with Hap5 and three accessions with Hap1 (**Fig. S8**), and found that the SNP (A/C, 1313) was linked with the 3 bp deletion (TGA/-, 437) in the CDS region. The TGA deletion in Hap1 led to a deletion of Asp (D), and the SNP variation in Hap1 changed the Ile (I) to Leu (L) (**Fig. S9**). We predicted the structure of GmGDPD2-Hap5 and GmGDPD2-Hap1 through I-TASSER61 and found that the two variations may greatly change the protein structure (**Fig. S10**). Although the 3 bp deletion did not occur in the conserved domain, amino acid mutations changed the 3D structure of the protein, which may be a major reason for the functional difference. An additional description on this point has also been added to the text (Lines 251-263).

It's unclear why authors didn't show expression of GDPDP2 gene under native promoter. Haplotype analysis shows unique haplotypes in promoter for Hap5.

Response: Thank you for this great suggestion. It would be more useful to gain more precise evaluation of the function using the native promoter driven GDPD2 in transgenic assay. Our promoter activity assay indeed demonstrates that hap5 is statistically higher in the activity than hap1. However, because the hap5 promoter is about 1.5-fold higher than hap1 promoter (**Fig.1L**), we have a concern that the native promoter cannot trigger the strong increase of GDPD in the transgenic plants as the

constitutively expression promoter 35S. We will test it at a later moment. Thank you.

It was claimed that, Hap5 shows significant higher root relative area as compared to other haps, did author tested the SNPs underlying this Hap? authors are encouraged to develop appropriate markers to discriminate P tolerance and sensitive accessions which can confirm the functional role of GDPDP2 in breeding program.

Response: Thank you so much for the important and valuable suggestions. We think the 3 bp indel in the CDS region of *GmGDPD2* was the key variation that affected the LP-tolerant and LP-sensitive genotypes. We designed KASP (Kompetitive Allele Specific PCR) markers based on the 3 bp Indel and verified that the KASP marker can effectively distinguish Hap5 from other Haps in 45 lines randomly selected from the 367 soybean accessions. Interestingly, we examined 34 soybean cultivars from the Huang-Huai-Hai Area of China (one of the three main soybean producing areas) and identified six of them as harboring Hap5 (**Table S6, new Table**). We hope this functional molecular marker can be applied to the breeding of soybean with high Pi efficiency. An additional description on this point has also been added to the text as "*We developed KASP (Kompetitive Allele Specific PCR) markers based on the TGA deletion (S437) and confirmed that the indel can effectively distinguish Hap5 from other Haps in a panel of 45 lines that were randomly selected from the 367 soybean accessions …*" (Lines 255-260)

GmGA2ox1 localized on the both nucleus and cellular membrane, did author tried other stain like DAPI to confirm the nuclear localization?

Response: This is a great suggestion. As you suggested, we redo the subcellular localization experiments with additional DAPI for GmGA2ox1 (**Fig. 4E**) and GmMyb73 (**Fig. 3C**) to confirm the nuclear localization. The results show that GmGA2ox1 localized to both the nucleus and cellular membrane and that GmMyb73 localized to the nucleus.

Merged figure of subcellular localization in case of Fig. 1I and 4E looks similar.

Response: Thank you for your careful review. We redo the two experiments for GmGDPD2 and GmGA2ox1 again separately. The new images are shown in **Fig. 1I** and **Fig. 4E** in the revised manuscript.

Put the appropriate scale bars for root traits images throughout the manuscript.

Response: Thank you for your suggestion. We have added scale bars for root trait images throughout the revised manuscript (**Fig. 2B, 2G, 3F, 5A**).

In Fig. 1I, 3C - what does the scale bar indicates?

Response: Thank you for pointing out this problem. Bar = 10 μm. We have added a description of the scale bar in **Fig. 1I, 3C, and 4E** in the figure legend.

Reviewer #3 (Comments for the Author):

The manuscript identifies a genomic locus associated with several seedling root traits under low phosphate availability in soybean, and progresses this to identify the underlying gene, SNP and functional implications of these in terms of plant responses to low phosphate availability.

Response: Thank you for your encouragement and helpful suggestions.

Major comments.

1. The Introduction and Discussion sections are very selective on the literature they cite, and do not acknowledge many papers in this area that have direct relevance to their findings either in soybean or other plant species. This should be addressed in any resubmission.

Response: Thank you so much for this important comment. We reviewed related papers and summarized the progress with some more representative papers in the Introduction section: "*Plants have evolved a series of adaptive responses, including modified root architecture and hormone regulatory effects, genetic changes, and domestic adaptation, to increase Pi acquisition and meet their growth and survival needs (Parry, 2018; Wang et al., 2022a)...*". (Lines 67-145)

2. Methodologically the manuscript is mostly sound. However, as is customary when evaluating OE and KO transgenic lines, data from multiple independent OE or KO lines are presented to demonstrate consistency between transformation events, which is lacking from this manuscript.

Response: Thank you for your encouragement and helpful suggestions. We obtained three duplicate phenotypic data points from at least three independent transgenic strains for all the transgenic strains mentioned in this manuscript. However, the mean values were selected to plot the data due space limitation. In the revised manuscript, we have modified the figures to show all the independent lines in Figures **S12, S13, S16, S17, S20,** and **S23** according to your suggestion.

3. There is also a lack of detail on how the phenotypic traits were measured, the levels of replication used and how they were analysed.

Response: Thank you for this suggestion. We provided more details on phenotyping of the plants grown in hydroponics, field experiment, root scanning, examination of PC, PAE, and seed quality (Lines 605-676). All experiments were independently replicated at least three times (Lines 616-617). Methods of statistical analysis was rewritten in the revised manuscript (Lines 678-691).

3.1 For example, why were the plants grown over two years? Where all the lines grown at once, or were they spread over the two years, or all lines grown in both years? How were the experiment-to-experiment variations accounted for in the analyses? How many replicates per line per treatment per year were utilized?

Response: We again thank the reviewer for raising this concern. Low phosphate stress is a complex quantitative trait that is greatly affected by the environment. Phenotypic identification at multiple sites over the years is important in assessing trait stability and breeding availability. All the lines were cultivated in the same environment, and it was repeated in the next year. The ANOVA results show that the differences of experiment-to-experiment (year-to-year) are mainly caused by environmental effects. For each treatment of each line, we tested the traits of three individuals. We have modified the method to make this clear (Lines 629-630, 646-656, 678-691).

3.2 Root traits are notoriously variable. How were the root traits quantified - no details are given. For the root trait measurements, no indication of replication level is provided. This is critical, given the highly variable nature of root traits.

Response: Thank you for your suggestion. The root-related traits of RIL population, 367 soybean materials, wild type and transgenic lines were measured by hydroponics, and the nutrient solution was updated every 2 days to ensure the consistency of root growth environment. Each line was repeated 5 times, and finally the best 3 with consistent phenotypes as the replicates were selected for subsequent analysis. For root related traits, they were analyzed with an automatic root scan apparatus (EPSON Expression 11000XL) equipped with WinRHIZO software. First, place the cleaned root in the transparent root plate, add 2 mm deep purified water to the plate, cover the plate with cardboard. Second, scan the root with 400 dpi resolution ratio, save root image as tiff file. Then, WinRHIZO software was used to analyze root images and obtain phenotypic data of root-related traits. A description on this point has been added to the text as "*Each hydroponics experiment was independently replicated at least three times.*" (Lines 616-617), and "*The RL, RN, RA, RD and RV traits were analyzed with an automatic root scanning apparatus (EPSON Expression 11000XL) equipped with WinRHIZO software. First, place the cleaned root…*" (Lines 657-663).

3.3 The ANOVA used for phenotypic data only includes genotype and year-location as fixed factors - was treatment not included as a factor in this analysis?

Response: We apologize that we did not clarify this clearly previously. The two environments refer to two independent repeated experiments conducted in 2018 and 2019 rather than different phosphorus treatments. We use the ratio of the phenotype between LP and NP, the ratio values were used as phenotypes for linkage mapping and GWAS. Taking the RRL (relative root length) trait as an example, we evaluated the root length of the population under the NP treatment and LP treatment in the same environment at the same time. Then, we calculated the relative root length through the following formula:

RRL (relative RL) $=\frac{\text{RL under LP treatment}}{\text{RL under NP treatment}}$

That is, the LP treatment factors we have already considered in the formula. We have modified the method to make it clear, described as "*To well reflect the LP tolerance response, we used the ratio of measured values for each trait for one line under LP relative to NP as an LP-related trait, for example, RRL (relative RL) = RL under LP treatment/RL under NP treatment.*". (Lines 651-654).

4. Figure 8 contains some speculative elements - Organic acids were not quantified, so they should not be present. What is meant by the 'Root-specific Growth factor'? Not all the genes to the left of the root tip were quantified in the manuscript, so these should be refined and only mention those quantified. There are also no data presented on P reutilization being driven by GmGDPD2, so this should be removed.

Response: We thank the reviewer for this important point. As soon as we received the review comments, we immediately designed experiments to quantify the changes in organic acids (oxalic acid and malic acid) (**Fig. S14, new Figure**) and gene expressions for some genes such as *GmPT7* and *DELLAs* (**Fig. S16H-K, new Figure**). The organic acids and selected genes were differently exudated or expressed between OE-*GmGDPD2* and KO-*GmGDPD2.* To avoid the confusion, we therefore updated the **Fig. 8** (**Fig. 7** in the revised manuscript) with new validated result and removed some untested results. An additional description on this point has also been added to the text as *"We observed that overexpression of GmGDPD2 enhanced the exudation of two organic acids (Fig. S14). For example, OE-GmGDPD2 roots exudated 8.99%-128.00% more oxalic acid and 1.79%-89.30% more malic acid than WT1 under NP or LP condition, ...*" (Lines 303-308, 345-346).

5. The use of the term 'Myb73-GDPD2-GA2ox1 module' in the title implies these genes are linked in some way, such as an operon or all physically interact as a tri-mer. However, the evidence to demonstrate the physical interaction of all three at the same

time is not presented, so this should be revised.

Response: We agree with it and thank you for the great suggestion. We change the title to "A transcriptional regulatory module, *Myb73-GDPD2-GA2ox1*, confers phosphate deficiency tolerance in soybean", which should well reflect the relationships among the genes.

6. Overall, the manuscript would benefit from editing for English language.

Response: Thank you for your suggestion. We have improved the English language of the manuscript by an English editing agent with an English native speaker to ensure that points are clearly stated.

Minor comments.

L33 - replace 'controls' with 'associated with'

Response: Thank you. We have replaced 'controls' with 'associated with'.

L59 - plants actively take up phosphorus, so replace 'absorb' with 'taken up'

General point - plants take up phosphate not phosphorus and fertilizers are always phosphate based. So careful use of the abbreviation of phosphorus to P is needed when referring to the uptake of phosphate and phosphate based fertilizers.

Response: Thank you. We have replaced 'absorb' with 'taken up' (Line 58) and check it through the text. We also have reworded the abbreviations of 'phosphorus' to 'P' and 'phosphate' to 'Pi' throughout the whole manuscript.

L61-64 - costs of fertilizers are country specific, so this needs to be acknowledged and a reference for these data provided.

Response: Thank you. We have modified it as "*It is estimated that nearly 1/3 of global cropland exhibits P deficiency, which requires P fertilization to improve crop yield (Bates, 1997; Lynch, 2011).*". (Lines 56-57)

L80-84 - one of the most significant impacts of phosphate deficiency on soybean productivity is the restriction it places on the development and functioning of the nitrogen fixing symbiosis with rhizobium - this should be acknowledged here.

Response: Thank you. We have added the corresponding description as "*Pi deficiency is more problematic than other nutrient deficiencies in constraining soybean yield production (Gowin, 1997; Abdelrahman et al., 2018). It may cause an increase in the root-to-shoot growth ratio and severe flower and pod abscission rates…*" (Lines 101-106).

L104 - the authors cite one study utilizing GWAS for identifying adaptations to low phosphate availability, but there are many others that should be acknowledged here - Wang et al 2022a, b, Du et al 2020, Yang et al 2020, Lu et a; 2018, plus probably others I didn't find with a simple search.

Response: Thank you. We have added the corresponding description and reference as "*Soybean cultivars differ greatly in LP responsiveness (Lu et al., 2018; Wang et al., 2019; Yang et al., 2020b; Wang et al., 2022b), and multiple QTL have been identified using a variety of LP-associated traits, such as root-related traits, biomass, organic acid content, Pi content and efficiency,...*" (Lines 109-114).

L108 - replace 'controls' with 'associated with'

Response: Thank you. It has been revised as you suggested.

L158 - replace 'controlling' with 'associated with'

Response: Thank you. It has been revised as you suggested.

L172 - replace 'after' with 'in'

Response: Thank you. It has been revised as you suggested.

L183 - Arabidopsis GDPDL2 has previously been shown to be responsive to low phosphate availability - this should be acknowledged - see Cheng et al., (2011) The Plant Journal (2011) 66, 781-795.

Response: Thank you. We have added this reference and other references reporting that the GDPD gene is related to low phosphate availability. We have added the corresponding description and reference as "*In Arabidopsis, Cheng et al. (2011) characterized GDPD family genes and revealed that the expression of most members could be induced by Pi starvation and that mutation in AtGDPD1 decreased Pi content in Pi-starved conditions. Si…*". (Lines 520-524)

L213 - replace 'absorption' with 'up take' - absorption implies a passive process.

Response: Thank you. It has been revised as you suggested.

L254 - mentioned 'in the field' but no field trials are described - do you mean in the growth chamber?

Response: Thank you. It should be "greenhouse", and we have modified it.

L282 - revise to read 'GmGDPD2 may be negatively regulated in planta by

GmMyb73'

Response: Thank you. It has been revised as you suggested.

L326 - where you say 'transgenic plant' do you mean OE lines or both OE and KO lines?

Response: Thank you. 'transgenic plant' in this line means overexpressing lines, and we have revised it to make it clear (Lines 408-409).

L341 - Revise to read 'Given the potential for physical in planta interaction between...'

Response: Thank you. It has been revised as you suggested.

L345 - replace 'severer' with 'greater'.

Response: Thank you. It has been revised as you suggested.

L353 - several papers have show a role for GA in plant adaptations to low phosphate availabilities, so to say this is 'rare' is incorrect.

Response: We are very grateful to the reviewer for the reminder. We have revised it to "*GA is an essential hormone for root growth and development (Ubeda-Tomás et al., 2008) and plant adaptation to low Pi availability (Jiang et al., 2007; Zhang et al., 2019b; Gualano et al., 2021).*" (Lines 446-448).

L367 – GAs

Response: This has been corrected. Thank you so much.

L372 and 375 - delete 'greatly'

Response: Thank you, corrected.

L381 and 382 - delete 'much'

Response: Thank you, corrected.

L410 - refers to root hairs, but these were not quantified, so this is speculative and should be removed.

Response: Thank you, corrected.

L418 - is this a new pathway? - both the GDPD2 (Cheng et al., 2011) and GA2ox1 (Zhang et al., (2019) Plant Sci 283, 177-188) have been identified in previous studies as associated with tolerance to low P availability in other plants.

Response: Thank you. We regard it as a new pathway because (1) *GmGDPD2* underlies a major QTL for multiple low-P-associated traits, as revealed in both GWAS and linkage mapping, demonstrating its importance in LP responsiveness in soybean and likely in other plant species; (2) *GDPD2* and *GA2ox1* were characterized in previously single studies, and their relationship has not characterized. Instead, our study connects both protein by Y2H and further reveals an upstream regulator Myb73, which is new Low-P tolerance regulator. Importantly, we link the three components to a regulatory module that have yet been reported previously. Based on these considerations, we think it is a new pathway.

L462 - likely downstream or regulated by an independent pathway?

Response: Thank you. We speculate that these genes may act downstream of *GmGDPD2*, but how they influence these genes remains to be investigated.

Prof. Dan Zhang
Henan Agricultural University
College of Agronomy
Zhengzhou
China

Dear Dr. Zhang:

We have received reviews of your manuscript entitled "A transcriptional regulatory module, Myb73-GDPD2-GA2ox1, confers phosphate deficiency tolerance in soybean." Thank you for submitting your best work to The Plant Cell. The editorial board agrees that the work you describe is substantive, falls within the scope of the journal, and may become acceptable for publication, pending revision and potential re-review.

Generally, and as noted by Reviewer #4 below, your revisions look good and the manuscript is acceptable to move forward. However, we noticed that your BiFC experiment is still lacking appropriate negative controls. We point to this commentary for guidance in our previous decision letter: https://academic.oup.com/plcell/article/28/5/1002/6098458
Please see Figure 1 and the text under "Essential Controls". In particular, there is a sentence that says "Inappropriate controls for BiFC experiments that, unfortunately, are frequently seen in the literature include, for example, combination of one of the interaction partners with an empty vector (expressing the unfused FP fragment) or the expression of only one of the fusion proteins (Horstman et al., 2014; Figures 1G to 1N)." In your BiFC experiment in Figure 4D, I believe that it is missing the appropriate negative controls. Unfortunately, this means you will need to repeat this experiment and it will require additional constructs. I am sorry for the added delay and work that this will necessitate, but these controls are important, and we try to be consistent in our publication requirements, although there is surely a paper or two that we have published when we missed these issues.

Please contact us if there are ambiguous comments or if you wish to discuss the revision.

Given the nature of the comments, we are offering you 60 days from when we have issued this decision to complete the revision. If a revision is not returned within this time frame, and if you have not been granted an extension, we will withdraw the manuscript, which will leave you free to submit the work elsewhere. If you need an extension, we encourage you to contact us at any point before the 60 days have passed.

When you are ready to submit the revised version, please upload a highlighted copy that indicates all changes made in response to the editor and reviewer recommendations. Include an itemized list of all changes made in response to each of the reviewer's suggestions in the "Response to Reviewers" section; please note that reviewers do not have access to your cover letter, nor was this decision letter shared with them.

Thank you for the privilege of reviewing your work. We look forward to receiving your revised manuscript.

On behalf of the editorial board,

Siobhán Brady, Senior Editor
Blake Meyers, Editor-in-Chief

The Plant Cell

Please note the following:

-The Plant Cell now requires authors to complete and submit an author revisions checklist upon submission of a revised manuscript. The aim of the checklist is to aid authors in preparing a high-quality manuscript, facilitate the review and assessment of revised manuscripts, and help to ensure that journal standards are maintained across the board. If your manuscript is accepted, the completed checklist will be published as supplemental material attached to the article online. Please download a copy of the checklist (pdf fillable form) at this link, for submission with your revised manuscript: https://tpc.msubmit.net/html/Author_Revisions_Checklist.pdf.

-Supplemental materials should be restricted to large datasets and tables, presentation of replicates, and validation of reagents, methods, or genotypes. Any data that are used to support the major claims must be in the main manuscript. Supplemental figure legends must indicate what figure in the main manuscript is supported by the supplemental data presented. Please justify how each of the supplemental figures meet the criteria.

-Sampling methods and nature of "biological replicates" should be described precisely (i.e. different plants, parts of

plants, pooled tissue, independent pools of tissue, sampled at different times, etc.), along with a clear description of and rationale for any statistical analyses conducted. The reader should know exactly what was sampled; what forms the basis of the calculation of any means and statistical parameters reported. This is also necessary to ensure that proper statistical analysis was conducted.

-Want to add this revision deadline to your calendar? Click below!

-- Reviewer comments:

Reviewer #4 (Comments for the Author):

The authors have taken my feedback into account and made significant improvements to the manuscript. After carefully reviewing the revised work, I can confidently say that I have no more comments to add.

Comments for the Author:

Generally, and as noted by Reviewer #4 below, your revisions look good and the manuscript is acceptable to move forward. However, we noticed that your BiFC experiment is still lacking appropriate negative controls. We point to this commentary for guidance in our previous decision letter: https://academic.oup.com/plcell/article/28/5/1002/6098458. Please see Figure 1 and the text under "Essential Controls". In particular, there is a sentence that says "Inappropriate controls for BiFC experiments that, unfortunately, are frequently seen in the literature include, for example, combination of one of the interaction partners with an empty vector (expressing the unfused FP fragment) or the expression of only one of the fusion proteins (Horstman et al., 2014; Figures 1G to 1N)." In your BiFC experiment in Figure 4B, I believe that it is missing the appropriate negative controls. Unfortunately, this means you will need to repeat this experiment and it will require additional constructs. I am sorry for the added delay and work that this will necessitate, but these controls are important, and we try to be consistent in our publication requirements, although there is surely a paper or two that we have published when we missed these issues.

Response: Thank you for pointing this out and this is a great suggestion. Following the suggestion, we selected the closely related proteins (Kudla et al., 2016, Plant cell, Figures 1D, 1E) as the negative control. GmGDPD (Glyma.02G100400.1) is a member of the same protein family with GmGDPD2, and was predicted located in the cellular membrane as GmGDPD2. GmGA2ox8 (Glyma.15G093900.1) is a member of the same protein family with GmGA2ox1, and is predicted to be located in the nucleus and cellular membrane as GmGA2ox1. Our new result showed that GmGDPD2 was interacted with GmGA2ox1, but GmGDPD2 was not interacted with GmGA2ox8, and GmGA2ox1 was not interacted with GmGDPD (Fig. 4B). We also revised the BiFC assay method as "*A. tumefaciens-mediated transient transformation of tobacco leaves was used for BiFC analysis. The CDSs of GmGDPD2 and GmGA2ox1 were PCR-amplified and cloned and inserted into the vectors 35S-SPYCE(M) and 35S-SPYNE173, respectively. We selected two new highly homologous proteins, GmGDPD (Glyma.02G100400.1) and GmGA2ox8 (Glyma. Glyma.15G093900.1), that were from the same protein family of GmGDPD2 and GmGA2ox1, respectively, as negative controls. GmGDPD and GmGA2ox8 showed 75% and 62% identities with GmGDPD and GmGA2ox1, and were predicted with same location as GmGDPD2 and GmGA2ox1 through DeepLoc-2.0 (https://services.healthtech.dtu.dk/services/DeepLoc-2.0/), respectively. The CDSs of GmGDPD and GmGA2ox8 were PCR-amplified, cloned, and inserted into the vectors*

35S-SPYCE(M) and 35S-SPYNE173, respectively. The fusion vectors were defined as GmGDPD2-YFPC, GmGDPD-YFPC, GmGA2ox1-YFPN, and GmGA2ox8-YFPN. Next, the following vector combinations (GmGDPD2-YFPC vs. GmGA2ox1-YFPN; GmGDPD2-YFPC vs. GmGA2ox8-YFPN; GmGDPD-YFPC vs. GmGA2ox1-YFPN; YFPC vs. YFPN) were cotransformed into 4-week-old N. benthamiana leaves. After 3 d, transformed tobacco leaf epidermal cells were imaged on a confocal laser scanning microscope (Zeiss LSM780, Germany). The fluorescence signal of YFP staining was imaged at an excitation wavelength of 488 nm." (line 933-950).

Dear Dr. Dan Zhang:

We are pleased to inform you that your paper entitled "A transcriptional regulatory module, Myb73-GDPD2-GA2ox1, confers phosphate deficiency tolerance in soybean" has been accepted for publication in The Plant Cell, pending a final editorial review by a science editor. At this stage, your manuscript will be evaluated by a science editor with respect to its presentation of scientific content, compliance with journal policies, and presentation for a broad readership. The Plant Cell has contracted with Plant Editors (planteditors.com) to provide this service to our authors, and you will soon receive additional information on this process.

Please note that each author needs to link their ORCID identifier to their account in the system before your manuscript can be published. If any authors do not have an ORCID linked to their account, they will receive a message with a link to complete this task. Please ensure that ALL of your coauthors have completed this task as soon as possible.

ASPB offers an OPEN option that allows authors to have their online articles available for free to all users immediately upon publication. For more information about the ASPB OPEN option, refer to the Final Submission Checklist Form.

The Plant Cell and The Arabidopsis Information Resource (TAIR) are collaborating to collect functional annotation data about Arabidopsis genes from authors. This includes information about the gene's molecular function (e.g., kinase activity, ATP synthetase activity), the biological process/es it is involved in (e.g., endosperm development, threonine biosynthesis), its subcellular location (e.g., nucleus, ER), anatomical or developmental expression pattern (e.g., leaf, ovule, flower stage 10, seedling stage), or its partner in a protein-protein interaction (e.g., AT1G01010 interacts with AT1G01020).

If your paper contains results falling into one or more of these categories for Arabidopsis genes, we request that you now submit these data for inclusion in TAIR by filling in the form provided at the following URL: https://www.arabidopsis.org/doc/submit/functional_annotation/123. If you need further clarification on what types of data can be submitted please contact curator@arabidopsis.org.

Finally, we encourage your submission of artwork for the journal cover. Monthly issues will have an online cover image and selected covers will be used for posters, other promotional items, and "wallpaper" for mobile devices. For more information, refer to Cover Submission in the Instructions for Authors https://tpc.msubmit.net/cgi-bin/main.plex?form_type=display_auth_instructions.

We look forward to seeing your paper published.

On behalf of the editorial board,

Reviewing Editor, Board of Reviewing Editors
Siobhán Brady, Senior Editor
Blake Meyers, Editor-in-Chief

The Plant Cell

=====
IMPORTANT REMINDER: PEER REVIEW REPORTS
=====

If you opted to publish a peer review report along with your article during the original submission process, it will be prepared by the editorial staff and publicly posted with your manuscript, inside the zip file that contains any other supplemental material. As a reminder, the peer review report is a public record of all comments from editors and reviewers, as well as your prior responses, as you received them in the decision letters for each draft of your manuscript. If you agreed to publish this report and have changed your mind, or are not sure if you selected this option, please contact the editorial office as soon as possible before signing the license agreement from our publisher.

=====

----FOR ASPB OFFICE USE ONLY (DO NOT EDIT)----
MSID: 34908
Corresponding Author: Prof. Zhang
